# Fragment-based design of small molecule PCSK9 inhibitors using simulated annealing of chemical potential simulations

**DOI:** 10.1371/journal.pone.0225780

**Published:** 2019-12-05

**Authors:** Frank Guarnieri, John L. Kulp, John L. Kulp, Ian S. Cloudsdale

**Affiliations:** 1 Center for Drug Discovery, Northeastern University, Boston, MA, United States of America; 2 PAKA Pulmonary Pharmaceuticals, Acton, MA, United States of America; 3 Conifer Point Pharmaceuticals, Doylestown, PA, United States of America; 4 Department of Chemistry, Baruch S. Blumberg Institute, Doylestown, PA, United States of America; National Library of Medicine, UNITED STATES

## Abstract

PCSK9 is a protein secreted by the liver that binds to the low-density lipoprotein receptor (LDLR), causing LDLR internalization, decreasing the clearance of circulating LDL particles. Mutations in PCSK9 that strengthen its interactions with LDLR result in familial hypercholesterolemia (FH) and early onset atherosclerosis, while nonsense mutations of PCSK9 result in cardio-protective hypocholesterolemia. These observations led to PCSK9 inhibition for cholesterol lowering becoming a high-interest therapeutic target, with antibody drugs reaching the market. An orally-available small molecule drug is highly desirable, but inhibiting the PCSK9/LDLR protein-protein interaction (PPI) has proven challenging. Alternate approaches to finding good lead candidates are needed. Motivated by the FH mutation data on PCSK9, we found that modeling the PCSK9/LDLR interface revealed extensive electron delocalization between and within the protein partners. Based on this, we hypothesized that compounds assembled from chemical fragments could achieve the affinity required to inhibit the PCSK9/LDLR PPI if they were selected to interact with PCSK9 in a way that, like LDLR, also involves significant fractional charge transfer to form partially covalent bonds. To identify such fragments, Simulated Annealing of Chemical Potential (SACP) fragment simulations were run on multiple PCSK9 structures, using optimized partial charges for the protein. We designed a small molecule, composed of several fragments, predicted to interact at two sites on the PCSK9. This compound inhibits the PPI with 1 μM affinity. Further, we designed two similar small molecules where one allows charge delocalization though a linker and the other doesn’t. The first inhibitor with charge delocalization enhances LDLR surface expression by 60% at 10 nM, two orders of magnitude more potent than the EGF domain of LDLR. The other enhances LDLR expression by only 50% at 1 μM. This supports our conjecture that fragments can have surprisingly outsized efficacy in breaking PPI’s by achieving fractional charge transfer leading to partially covalent bonding.

## Introduction

Efficient removal of LDL particles from the blood stream is an essential process for preventing hypercholesterolemia and its associated atherosclerosis. The current understanding of the importance of a properly functioning LDL uptake system has come from a series of pioneering genetic studies on families prone to heart disease early in life. In 1978 Goldstein and Brown[1] mechanistically identified and described a mutation in the LDLR as a cause of familial hypercholesterolemia (FH). In 1987 Innerarity[2] and co-workers discovered a similar disease phenotype in patients with a mutation in the apolipoprotein gene that codes for the protein component of LDL. This body of work and other human genetic studies[3–20] provides a detailed picture of how arterial plaque deposits lead to heart disease.

**Fig 1:**
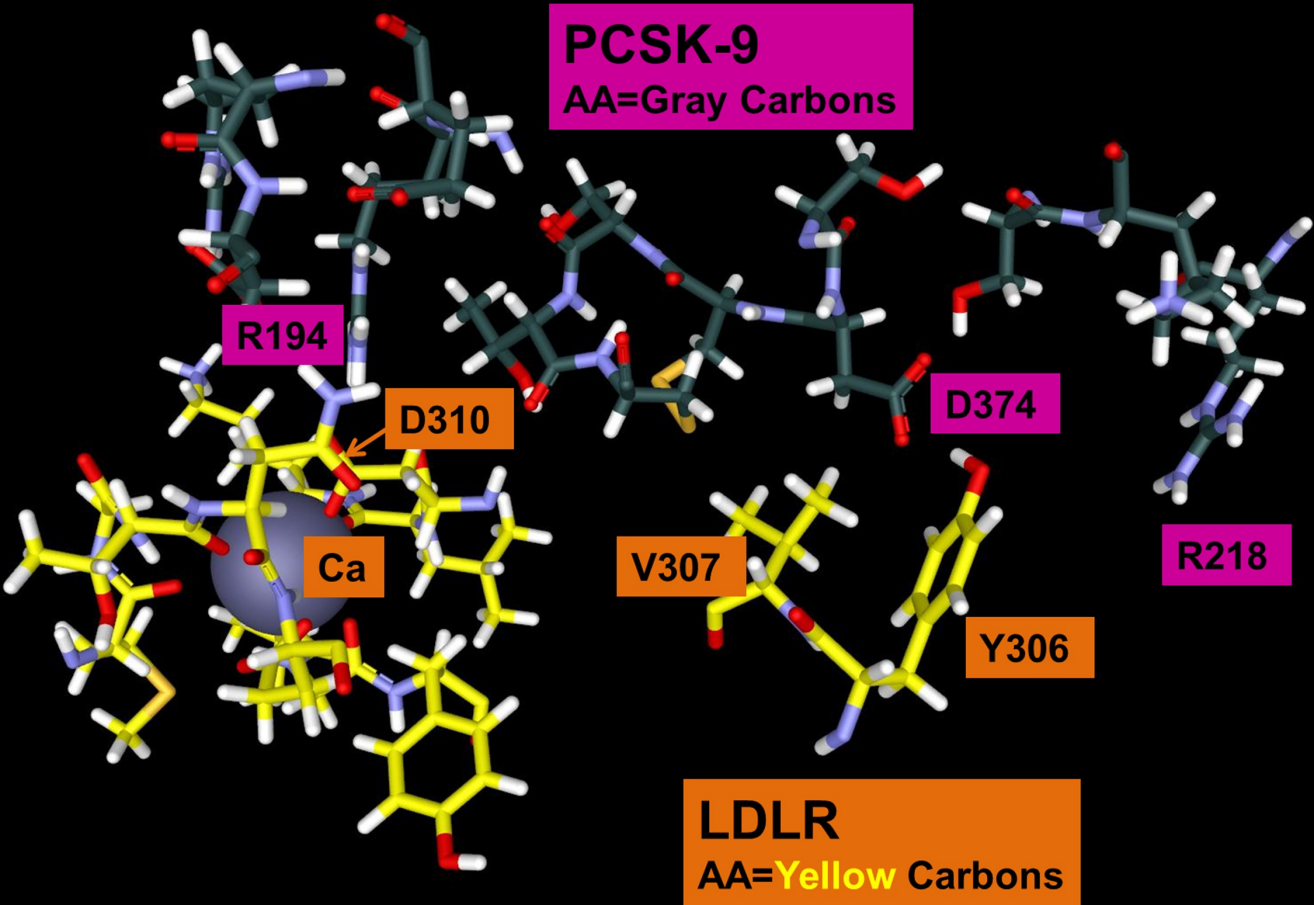
The PCSK9-LDLR interface from the PDB 3GCW with the H306Y FH mutant. The carboxyl group of LDLR D310 chelates the Ca^2+^ ion of LDLR and forms a salt bridge with R194 of PCKS9. R218 has no obvious partner on LDLR, but R218S is an FH mutant and so is included as part of the interface.

**Fig 2:**
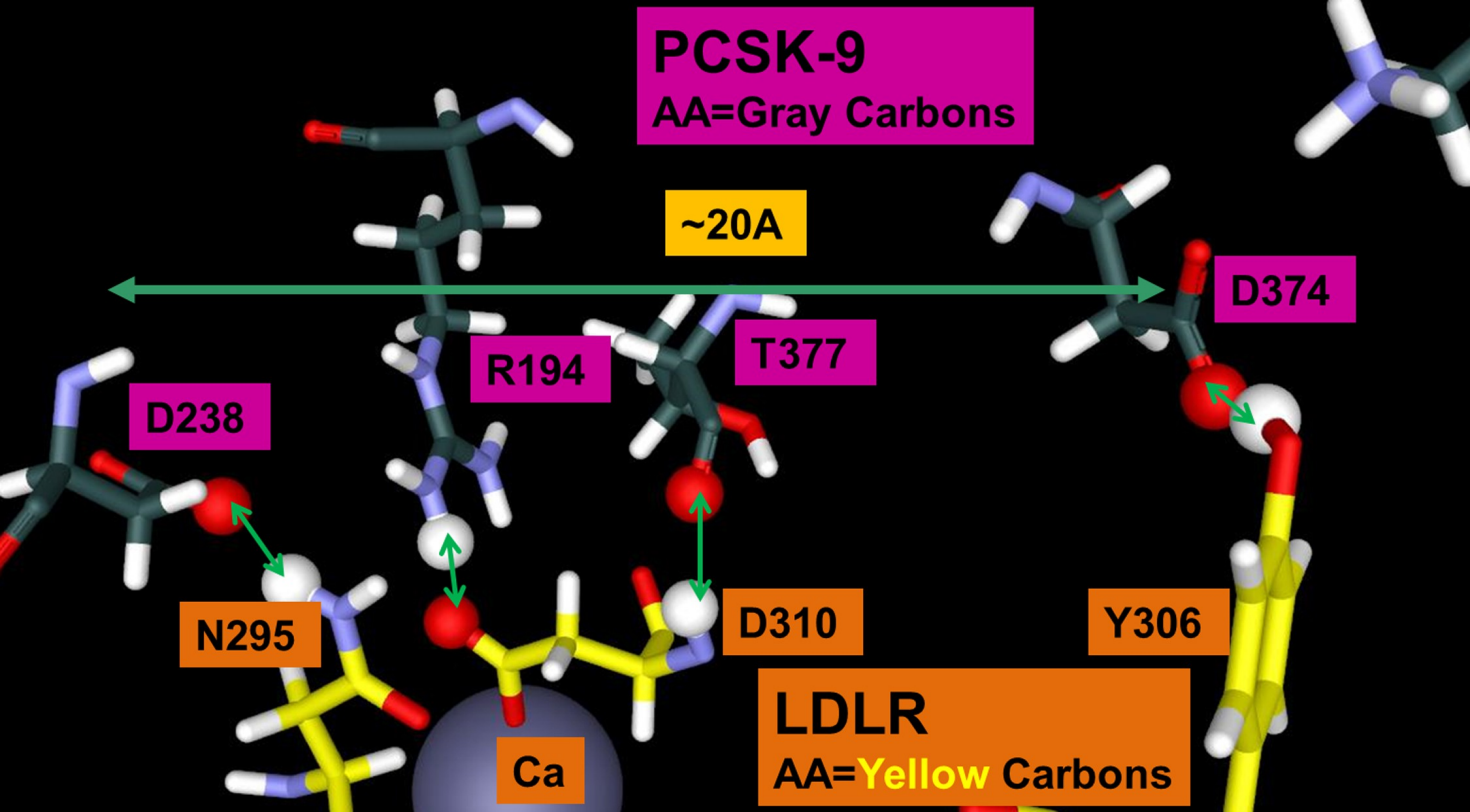
Four key PCSK9 interactions with LDLR. H306Y of LDLR shares its phenolic proton with D374 of PCSK9. LDLR D310 mediates electron sharing between the Ca^2+^ ion of LDLR and R194 of PCSK9 by simultaneously chelating the metal and forming a salt bridge with R194. Further, the backbone of D310 forms a hydrogen bond with the backbone of T377 from PCSK9. LDLR N295 chelates the Ca^2+^ ion and simultaneously forms a hydrogen bond with D238 of PCSK9. The PPI interface spans over 20 Å.

The key to translating basic research into practical drug discovery is target validation. This was achieved for PCSK9[21–27] with the finding that inactivating mutations resulted in individuals with low blood cholesterol, a history of no coronary artery disease, and, most importantly, no deleterious side effects. These longitudinal human studies confirmed the compelling impact of blocking PCSK9. Both Amgen[28–34] and Regeneron[35–44] have successfully brought inhibitory antibodies to the market, with FDA approval occurring in 2015. The early data indicate that these antibodies are a breakthrough in treating hypercholesterolemia and heart disease. It would obviously be highly desirable to have orally-available small molecule inhibitors of the PSCK9/LDLR interaction, because such compounds have the potential to be much more cost effective to produce than protein antibodies. This goal has been elusive due to the large and complex nature of the PCSK9/LDLR protein-protein interaction (PPI) as illustrated in Fig 1. Analysis of this structure indicates that there are 4 key interaction (Fig 2) sites that span a large distance.

Of these four sites, two sites are strongly influenced by the Ca^2+^ ion in the EGF domain of LDLR–the D310-R194 salt bridge and the N295-D238 hydrogen bond. The polarization by the Ca^2+^ ion raises a question of its role in the PCSK9/LDLR binding–is it most of the contribution to binding or only a limited part of it. This is important to an inhibition strategy. The FH mutation data indicates that the D374 region also plays a critical role in the binding through its interaction with residue 306 of LDLR, and R218 plays a key functional role while K222 appears to polarize D374. Lowering the pH can increase the PPI by two orders of magnitude and the H306Y LDLR is a gain of function FH mutation, indicating the importance of the D374 site. This led to a conjecture that the polarization of the two sites is related.

As a point of terminology, we refer to fractional charge transfer as a shifting of the average location of electron density from one molecule to another. In modeling, this shift is manifest in a change in partial charges derived using the RESP program from electrostatic potentials produced in GAMESS simulations of electron wave functions. A strong hydrogen bond may have a fractional charge transfer in the range of 0.1, a salt bridge a range of 0.2, and so-called cooperative hydrogen bonds, as in biotin/streptavidin binding, a range of 0.4. Generally, fractional charge transfers of > 0.2 lead to very strong binding, depending on desolvation penalties. Although charge delocalization can refer to the movement of charge within or between molecules, we generally reserve the term to refer to polarization within molecules or where no net charge is transferred between molecules.

To explore the conjecture, we modeled the interface residues with GAMESS/RESP[45] to assess partial charge changes. It was thought that using these optimized partial charges in modeling the interactions was important and that the standard Amber charges would be inadequate. Further, a detailed analysis of these interactions is an essential prerequisite for designing small molecules that can break this PPI.

## Theory

While it is arguably obvious that fractional charge transfer effects are occurring locally in the vicinity of the LDLR Ca^2+^ ion (calcium ion) that include the chelating groups and the salt bridge between LDLR D310 and PCSK9 R194, there is no *a priori* reason to believe that there are any other prominent effects between LDLR and PCSK9. Studying the structural biology of the PCSK9/LDLR complex, an observation was made that electron delocalization is likely occurring from the LDLR Ca^2+^ ion and chelating groups, through the PSCK9 R194 salt bridge and, less obviously, extending through a chain of PCSK9 residues from R194/T377 to the PSCK9 D374 and back to the LDLR H306. The potential for this delocalization residue chain is also observed in the H306Y FH mutant. Specifically, when PCSK9 binds to LDLR, the ligand-receptor complex is internalized into the cell and inserted into an acidic lysosome for the purposes of locally denaturing the binding site, releasing the PCSK9 ligand, and recycling the receptor back to the plasma membrane. Acid, however, strengthens[46] the PSCK9/LDLR interaction, so the entire complex has to be inserted into the lysosome and destroyed. This change in pH environment is important to the interaction. Thus, after internalization, there is little or no LDLR expressed on the cell surface until the receptor can be remade at the genetic level, resulting in prolonged inability to uptake LDL particles. *In vitro* studies of PCSK9/LDLR[47] confirm that lowering the pH dramatically enhances the PPI. It can be hypothesized that the reason H306Y is an FH mutation is because of the addition of a labile phenolic proton at this crucial interaction site. It is our conjecture that the electron delocalization over this extensive PPI interface is responsible for the high affinity PCSK9/LDLR interaction, which is strongly enhanced by the presence of a proton that also delocalizes over this extensive interface, acting as a super-tautomer.

**Fig 3:**
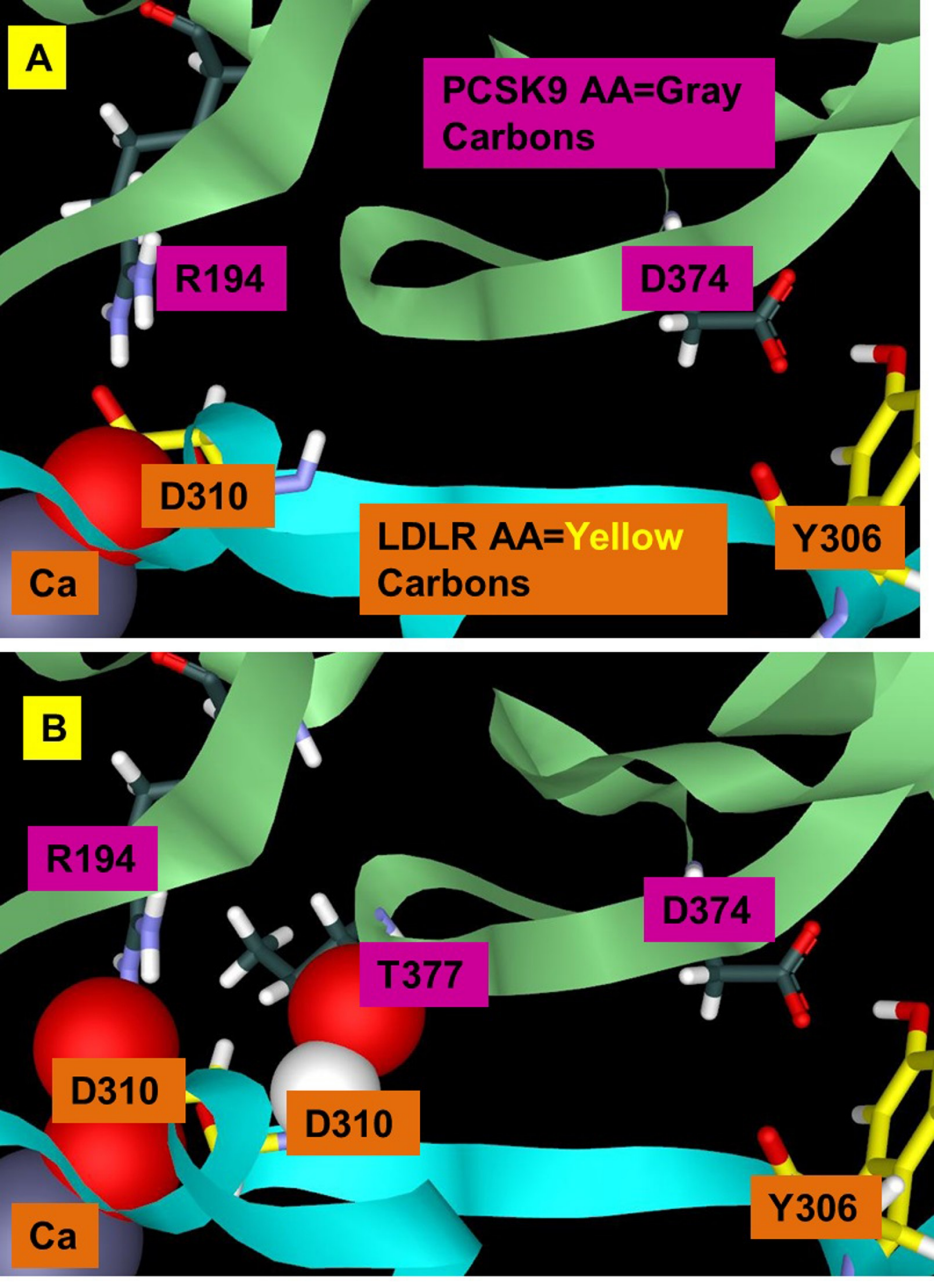
The LDLR D310 interactions with PCKS9. Conversion of one oxygen atom of the carboxyl group (A) from LDLR D310 to VDW representation shows how deeply it penetrates the LDLR Ca^2+^ ion as a chelating group. Converting both oxygens of the D310 carboxyl group (B) to VDW representation shows the continuous overlap from the LDLR Ca^2+^ ion to R194 of PSKC9. It is noteworthy that the D310 backbone hydrogen interpenetrates the ketone backbone of PCSK9 T377 (B).

**Fig 4:**
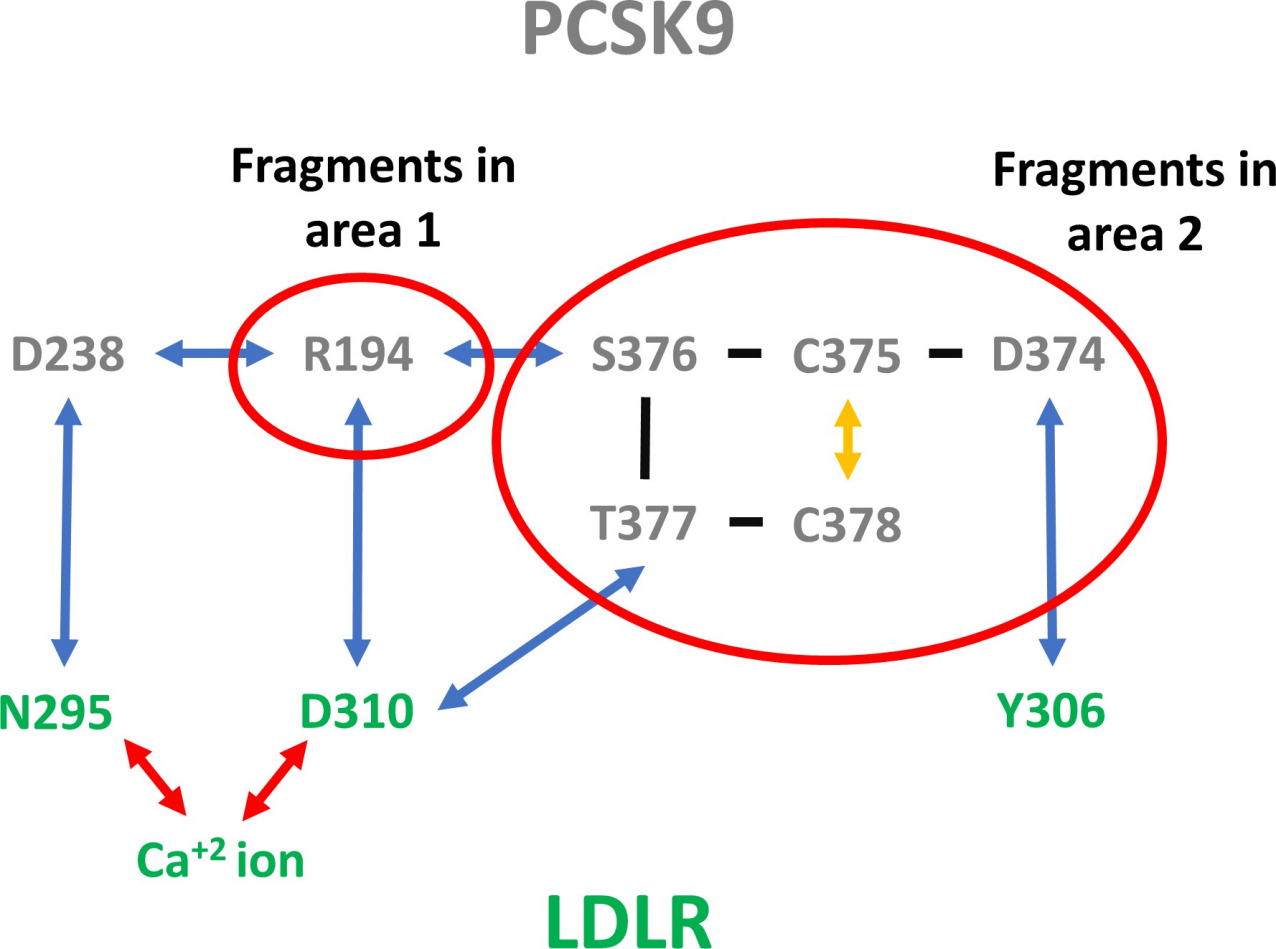
The network of charge delocalization, as observed from VDW overlap. LDLR residues are label with green font. The PCSK9 residues are labeled with grey font. Red arrows specify Ca^+2^ ion-LDLR bonds. Blue arrows illustrate hydrogen bonds. The yellow arrow marks a disulfide bond. Black dashes indicate sequential amino acids. The complete lists of residues were analyzed with a GAMESS/RESP calculation to compute optimized partial charges derived using the RESP algorithm to best fit the electrostatic potentials from electron densities produced by GAMESS (S1 Table). Using SACP simulations, we identified fragments in area 1 and area 2 that could break this network of charge delocalization.

To obtain computational evidence for the extensive electron delocalization, we analyzed the interface by systemically mapping out the van der Waals (VDW) overlap of heavy atoms internal to PCSK9 in order to identify networks in PCKS9 capable of potentially delocalizing electrons, which is illustrated in Figs 3 and 4 and S1. The VDW radii displayed are the R* radius in the Lennard-Jones model, the distance of minimum (favorable) energy.

The most obvious VDW overlaps occur where the D310 side chain of LDLR chelates the Ca^2+^ ion and also forms a salt bridge with R194 of PCSK9. What is less obvious is the fact that the backbone of D310 interpenetrates the T377 backbone of PCSK9. It is very interesting that this cyclic interaction motif of side chain and backbone also occurs with R194 –its backbone has VDW interpenetration with D238 of PCSK9 and this same D238 forms a hydrogen bond with N295 of LDLR and N295 also chelates the Ca ion. Between the Ca interaction site and the D374-Y306 site inside PCSK9, which is separated by ~20 Å, a small Cys-Cys disulfide ring exists and there is a continuous backbone VDW interpenetration from LDLR D310 to PCSK9 T377-C378-C375 that can be connected all the way to D374 and back to LDLR Y306. Ultimately, we identify >500 atoms that form the LDLR-PCSK9 interface inside PCSK9 that can potentially share and delocalize electrons. The complete set of residues, for which custom partial charges were derived using simulations, are shown in Fig 1 and listed in the supplementary materials (S1 Table).

Traditionally, it was difficult to break PPIs that have complex tertiary (3°) structure interactions with a fragment-based drug discovery (FBDD) approach or any small molecule ligand from high throughput screening. Thus, any hope of success targeting PCSK9/LDLR with fragments requires that there is something special and unique about this interaction that can be exploited. Our conjecture can be restated to make it relevant for FBDD–the extensive electron delocalization that occurs between LDLR and PCSK9 means that the interaction can be classified as highly polarized and partially covalent–and thus a very small fragment can have an outsized binding affinity for PCSK9 if it can be specially designed to bind to PCSK9 with a similar partial covalent character. A composite inhibitor can be constructed by linking fragments which interact at the key sites described. Although LDLR uses the calcium ion chelating aspartate D310, a designed inhibitor could use other functional groups to achieve delocalization.

Simulated Annealing of Chemical Potential[48] (SACP) is a sampling method[49] that generates a sequence of fragment-protein Boltzmann distributions[50, 51] as a function of chemical potential and has been successfully used in several protein-ligand[52–55] binding and drug discovery[56–60] studies. The success is predicated on being able to accurately rank fragments based on excess chemical potential. The best ranking is achieved if the protein atoms are modeled with custom partial charges that account for the complex nature of a specific interaction. The analysis of the extensive charge delocalization of the LDLR-PCSK9 interface motivates that context-specific partial charges be used in the simulations, as opposed to averaged Amber residue charges. Amber models assign partial charges to the atoms of an aspartate such as D374 of PCSK9 in a way that the sum of the partial charges on the residue adds up to -1. If the charge is delocalized in the PPI complex, the sum of the charges would become more positive in D374 due to electron delocalization into LDLR Y306. Interestingly, this leads to two predictions about what is expected to be seen in the 500-atom GAMESS/RESP simulations: 1) D374 should have a more positive sum of partial charges in the PCSK9/LDLR complex, and 2) the sum of charges on D374 in the PCSK9-LDLR-(H306Y) mutation should be even more positive, because this complex binds more tightly than the wild type PPI resulting in a greater extent of electron delocalization. There is a third prediction–there should be a dramatic change in the polarization of the T377 amide backbone. This is because this residue directly connects the VDW interpenetration from the Ca^2+^ ion site of LDLR, through D310, to the D374 site of PCSK9 through the residues connected with overlapping atoms. We will show that computed partial charges support these three predictions. The partial charges for PCSK9 used in the SACP FBDD simulations will be taken from the PCSK9-LDLR-(H306Y) GAMESS/RESP calculations. These represent a stronger interaction energy complex than using the wild type PCSK9, and thus should be compatible with fragments with higher binding affinity for PCSK9. This is then verified by doing protein-fragment GAMESS/RESP simulations for the fragment binding configurations produced by SACP simulations. Fundamentally, we want to discover fragments that bind to PCSK9 and share electrons with the protein–partially covalent binding–because we hypothesize that this could generate surprisingly large affinities for small fragments (MW < 200) that generally have no detectable binding at the PPI interface even at 5–10 mM concentration in experimental FBDD.

## Results: Computational GAMESS/RESP calculations on the PCSK9/LDLR interface

**Fig 5:**
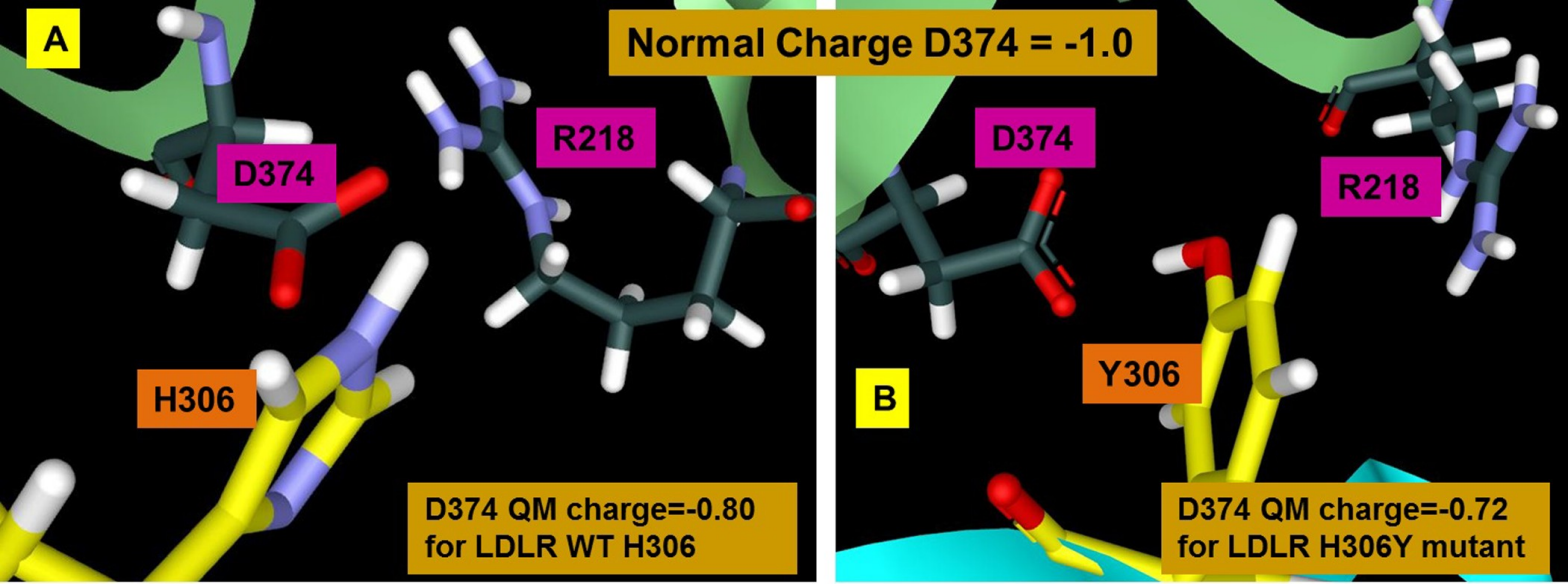
LDLR interaction changes the PCSK9 D374 charge. GAMESS/RESP calculations were run on the complex between PCSK9 and wild type LDLR (PDB ID 3bps) and on PCSK9 complexed with the FH mutant LDLR H306Y (PDB ID 3gcw) with all of the amino acids forming the extended interface shown in Fig 1. Amber parameters assign partial charges to the atoms of an aspartate such that the total charge on the residue is -1. The charge on D374 is reduced by 20% in the wild type (A) complex and by 28% in the FH mutant (B) complex.

**Fig 6:**
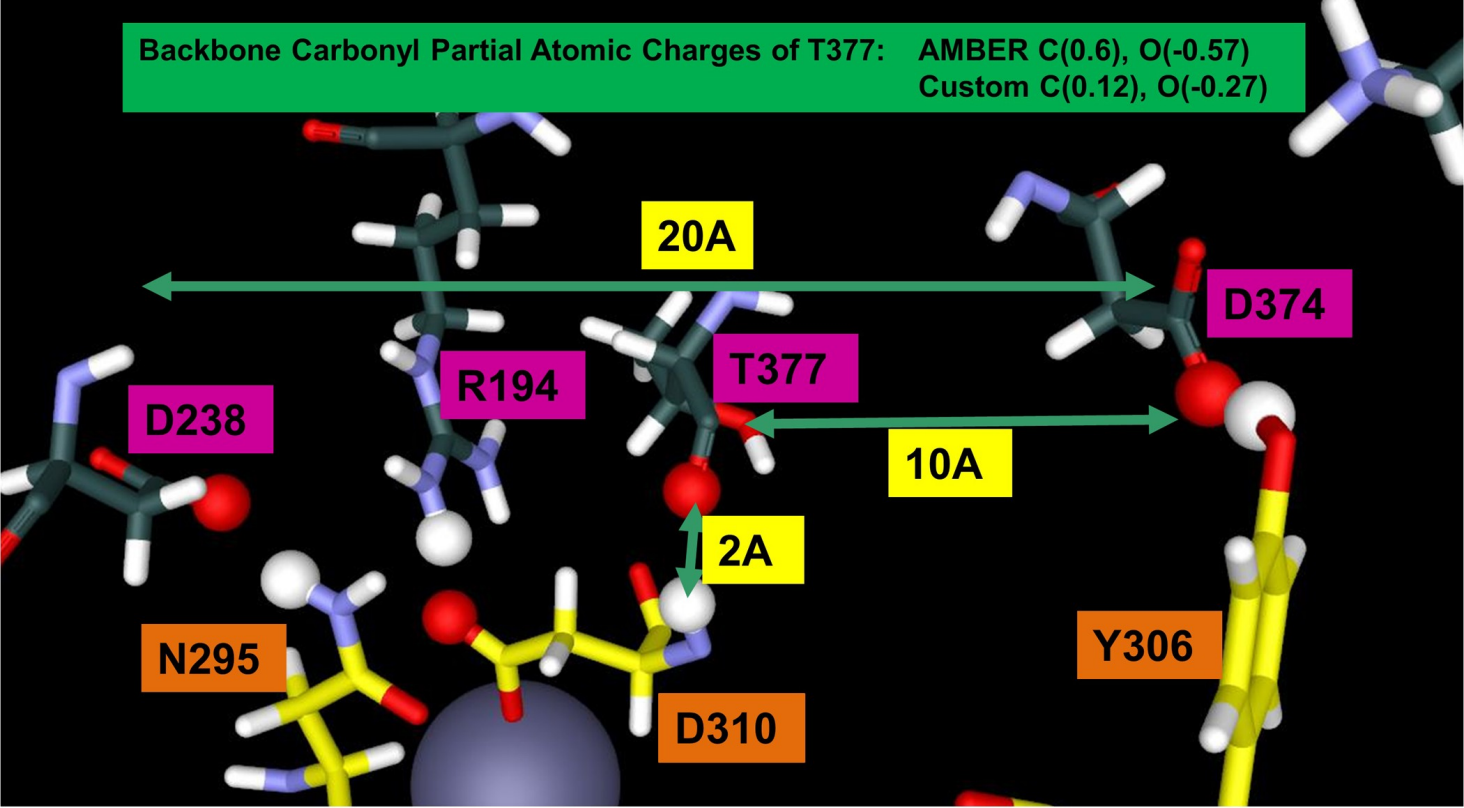
There is a dramatic change in polarization of the PCSK9 T377 backbone induced by LDLR binding. Standard partial charges from the Amber force field of a threonine carbonyl backbone are carbon (+0.60) and oxygen (-0.57). The GAMESS/RESP partial charges for the T377 of PCSK9 when interacting with LDLR-H306Y are carbon (0.12) and oxygen (-0.27), confirming that modeling indicates a profound and pervasive electron delocalization in this PPI.

Custom partial charges were derived for the set of residues shown in Fig 1 for the interface of PCSK9/LDLR wild-type and the PCSK9-LDLR-H306Y FH mutant. Comparing the partial charges from these different calculations reveals how the charges in the protein atoms can change when they are brought together and how these charges can change in the presence of the H306Y FH mutant. The results, Figs 5 and 6, reveal that fractional charge transfer is occurring at this PPI which may be an important component of the high affinity binding between PCSK9 and LDLR. For reference the Amber model charge of -1 on D374 in the wild type PPI is reduced in the GAMESS/RESP calculations by 20%, and by 28% in the FH LDLR-H306Y mutant (S1 Table). The backbone of T377 is dramatically altered from the Amber amide polarization with the partial charge on the carbon of the ketone changing by 80%. Normally PPI’s occur through non-bonded interactions (say, VDW, hydrogen bonding, hydrophobic effects), but when there is significant fractional charge transfer, the interactions may be classified as partially covalent, and thus can be unusually higher in affinity. There is a central aspect of the T377 charge delocalization result that could have a pragmatic bearing on fragment-based inhibitor design. Designing a molecule that directly disrupts PCSK9 binding to the Ca^2+^ ion site and also the Y306 site on LDLR simultaneously requires spanning 20 Å. The results indicate that this may be accomplished by simultaneously blocking the D374 site and polarizing the T377 site on PCSK9, which span only 10 Å, and thus can be achieved with a smaller, lower molecular weight compound. Blocking the Ca^2+^ ion binding directly by steric occlusion is not feasible due to the distances involved. These charge models can vary from real electron behavior due to various factors but act as a guide to bias the compound designs.

### SACP simulations on PCSK9 using custom partial charges

All of the amino acids of PCSK9 at the interface with LDLR-H306Y shown in Fig 1 were assigned partial charges from the GAMESS/RESP calculations of the PPI complex. SACP simulations on this PCSK9 structure were run using 1,217 different small chemical fragments (S2 Table). These include a wide range of very low molecular weight species (< 80 Da), which comprehensively represent all of the elementary organic chemistry functional groups, and somewhat larger fragments that generally are commercially available starting points for chemical synthesis of fragment screening libraries. Also run were higher molecular weight drug-like molecules (100–300 Da). Finally, water molecules are simulated to identify high affinity a water binding sites to take into account in compound design.

Originally, Kirkpatrick[61] introduced simulated annealing of temperature as a way of generating a sequence of Boltzmann distributions at decreasing temperatures in the canonical ensemble as an efficient way of searching for the global minimum of a hyper-dimensional cost function. This was specifically applied to very large scale integrated circuit design. SACP, conceived by Guarnieri[49], generalizes this approach by keeping temperature constant while generating a sequence of Boltzmann distributions at decreasing chemical potentials in the grand canonical ensemble. This is a rigorous way of determining relative fragment-protein binding free energies that uniquely carries out comprehensive sampling. The SACP simulation process has been described and illustrated[51] with multiple examples in a recent paper.

### Cyano-benzimidazole predicted to bind the D374-R218 site

**Fig 7:**
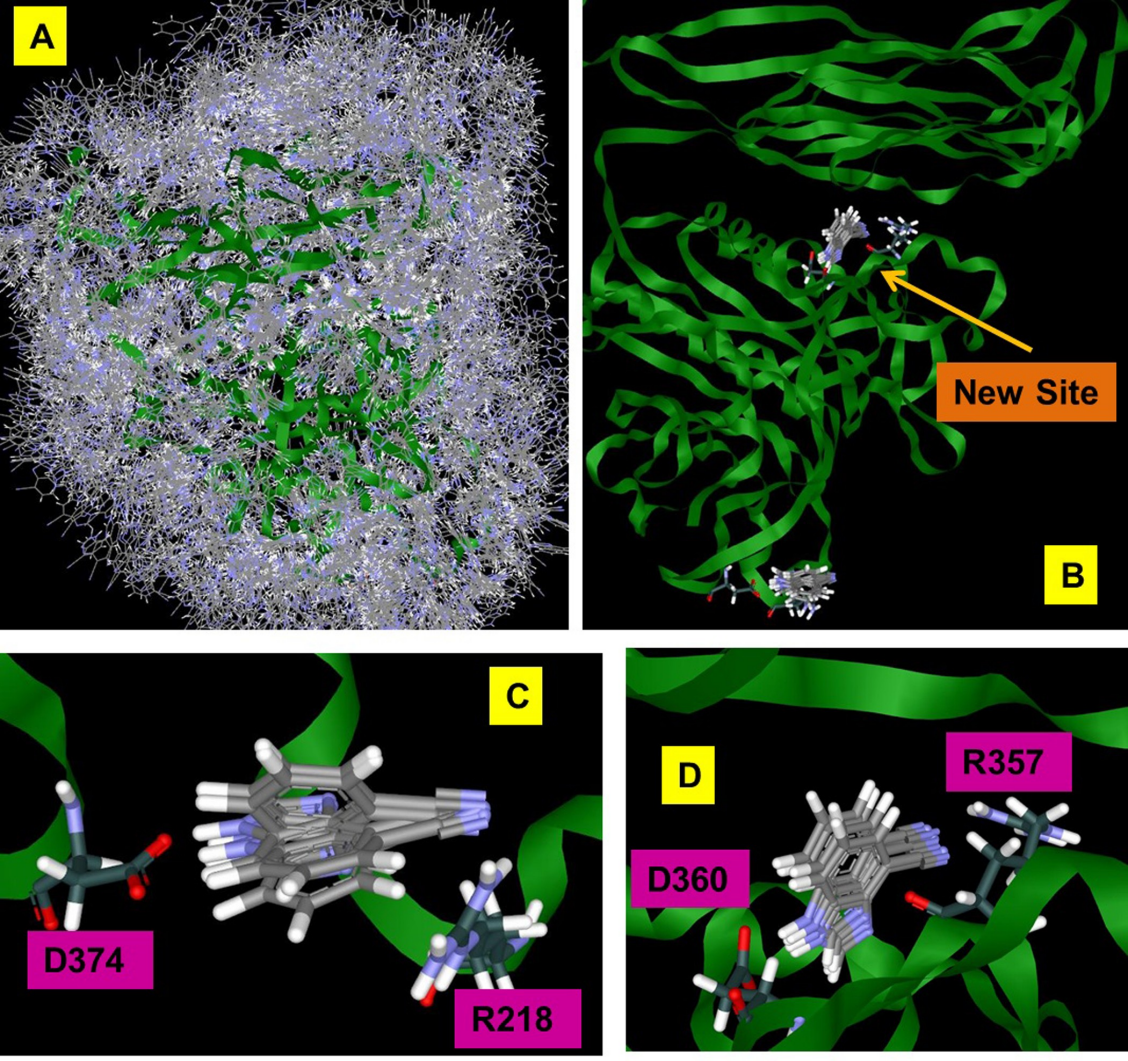
SACP on PCSK9 with cyano-benzimidazole. The high chemical potential (A) and lowest chemical potential (B) simulations identify the known high affinity (C) Y306 LDLR binding site and a (D) new site.

**Fig 8:**
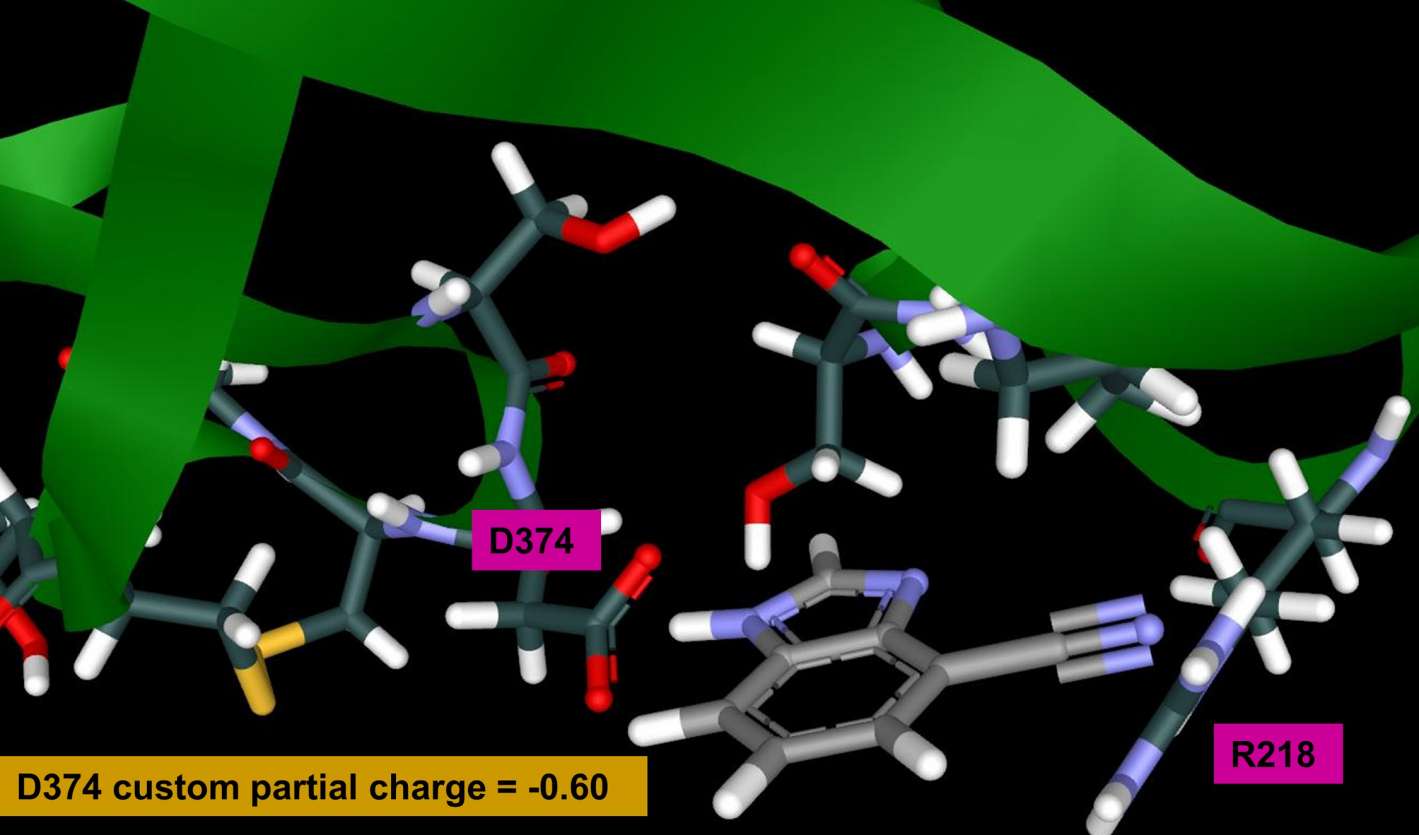
Cyano-benzimidazole is predicted to bind with the highest affinity (lowest chemical potential) at D374 of PCSK9. The GAMESS/RESP calculations on this configuration show that the sum of partial charges on D374 is reduced by 40%.

SACP samples fragment-protein interactions in a completely unbiased fashion over the entire protein surface and all possible cavities. The results for the cyano-benzimidazole were particularly intriguing as shown in Fig 7. Binding with the lowest chemical potential (highest affinity) takes place at two distinct sites, the known PCSK9/LDLR D374-Y306 locus with an imidazole proton interacting with the side chain of D374 and the cyano group interacting with R218, and a second site at the interface of two PCSK9 domains. To our knowledge this second site is not well described in the literature, and we know nothing about it except that SACP indicates that it is an important intramolecular PCSK9 PPI. These two protein-fragment interactions at these two particular locations are the highest affinity binding of all 1,217 fragments at all possible PCSK9 binding sites. Other binding sites arose from the simulations where fragments bind with very low chemical potential, at the serine protease catalytic triad for example. The cyano-benzimidazole, at the D374-R218 and D360-R357 sites, are the highest affinity interactions according to SACP with the custom charges at the D374-R218 site. The cyano-benzimidazole—with standard AMBER parameters—had an excess chemical potential, net of solvation, of -42.93 and with custom partial charges had an excess chemical potential of -45.42. GAMESS/RESP calculations were run with PCSK9 and just this cyano-benzimidazole fragment bound, as predicted by SACP, and the reduction in partial charges of D374 (Fig 8) seen is an astonishing 40% (S3 Table).

### Error tolerant linker strategy & creating custom fragments

How to covalently link[62–66] two distinct fragments that bind in close proximity at adjacent sites on a protein is a fundamentally difficult and mostly unsolved problem in FBDD. Linking is an error-prone task that can easily reduce or eliminate the affinity of the larger di-fragment (fragment pair). It is essential to have a linker strategy that maintains the optimal protein binding positions of both fragments simultaneously, and the linker should not change the electronic structure of the individual fragments. We have developed some strategies that mostly avoid dealing with the linker issue. The first goal in making a larger higher affinity molecule is to create a new custom fragment that, 1) maintains the high affinity binding of cyano-benzimidazole to the D374 site, 2) adds a new functional group that points towards T377, 3) appropriately interacts with the contours of PCSK9 such that no clashes occur, and 4) enhances fractional charge transfer manifest in partial covalent character.

**Fig 9:**
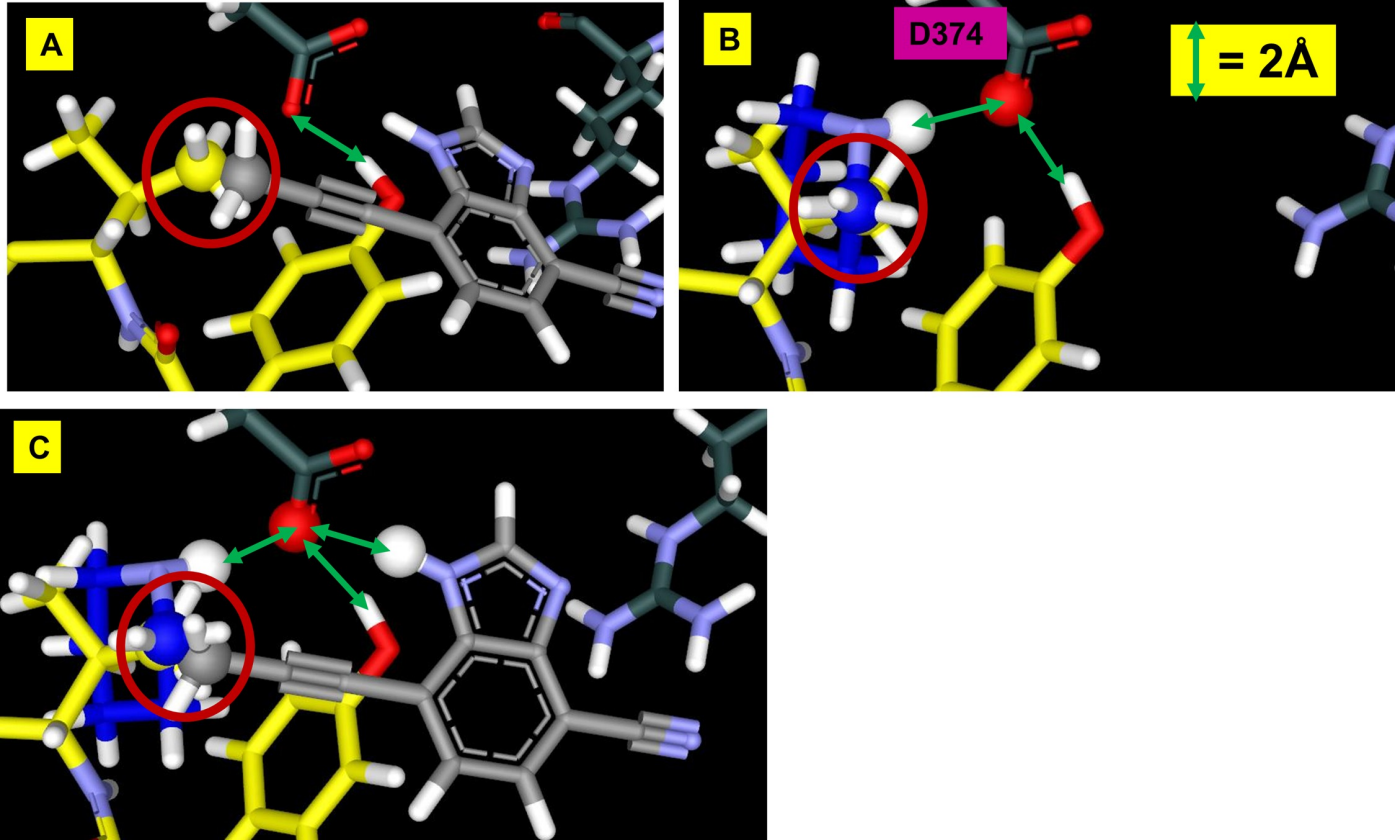
An acetylene linker is added to cyano-benzimidazole. A trial custom fragment was designed (A) and SACP confirmed that it tightly bound in the same mode as the original smaller fragment with the terminal carbon of the acetylene moiety occupying the same space as a terminal carbon of LDLR-Val307 (red ring). The piperidine fragment (B) binds with its carbons overlapping the side chain carbons of LDLR-V307 and pointing its N to form an H-bond with PCSK9-D374 and thus the two fragments (C) merge at LDLR-Val307 eliminating the error-prone linking process.

A particularly powerful aspect of SACP is the ability to test fragments that are customized for a special situation. In the case of PCSK9, analysis of the protein interface suggests that adding an acetylene group to the cyano-benzimidazole might be a di-fragment that satisfies aims 1 and 2 of the goal stated above. We made this fragment and ran SACP simulations on PCSK9 and the binding pose of the di-fragment was as predicted from the individual fragments, even mimicking some of the LDLR interactions. The hydrophobic acetylene functionality partially occupies the same position as the LDLR Y306-V307 locus (Fig 9A). It is important to note that it is very easy to introduce a subtle, unnoticeable clash when creating custom fragments, and if this inadvertently occurs, the fragment will bind in a completely different mode or not at all in the SACP simulations. The design error will be immediately revealed. A careful analysis of the interface indicated that the function of V307 was to provide hydrophobic protection for the D374-Y306 interaction, so a triple bond replacement for the bulky cluster of sp3 carbons was unlikely to cause any inadvertent clashes.

As fragments become larger, the SACP sampling becomes more inefficient with a higher percentage of rejections in the Monte Carlo simulation, especially at lower chemical potentials. Thus, we generally limit custom designs to di-fragments. To create tri-fragment molecules, we prefer to do fragment merging, because it removes much of the guesswork associated with linking. The process is started by examining where the new linker unit, in this case the acetylene, overlaps with the partner protein, in this case LDLR. The reason for this is quite straightforward. Successfully designing a custom fragment means that it mimics some of the key PPI interactions, otherwise it is a failed attempt. The addition of the acetylene linker was successful because the cyano-benzimidazole interaction was maintained and the terminal carbon of the acetylene occupies the same position as a side chain atom of LDLR-V307 as shown in Fig 9A.

To begin the fragment-fragment merging process, fragments from SACP are identified that bind to PCSK9 in a way that one of their carbon atoms merges with a terminal carbon atom of LDLR-V307. The reason for doing this is because the terminal carbon of the acetylene merges into a terminal carbon of this valine, so if a second fragment does the same thing at the same atom, then SACP predicts that these two distinct fragments can be merged into a tri-fragment by merging their carbons at this position. Another filtering criterion is that the subset of fragments that merge must have the proper bond angle. When this is combined with the D374 pincer condition (Fig 9C), only a few fragments pass all three criteria. Piperidine successfully satisfies all of the criteria as shown in Fig 9B and 9C. Interestingly, azepane, the 7-membered ring with 6 carbons and one nitrogen does not bind at this key site according to SACP, demonstrating the vital difference that even one atom can make.

**Fig 10:**
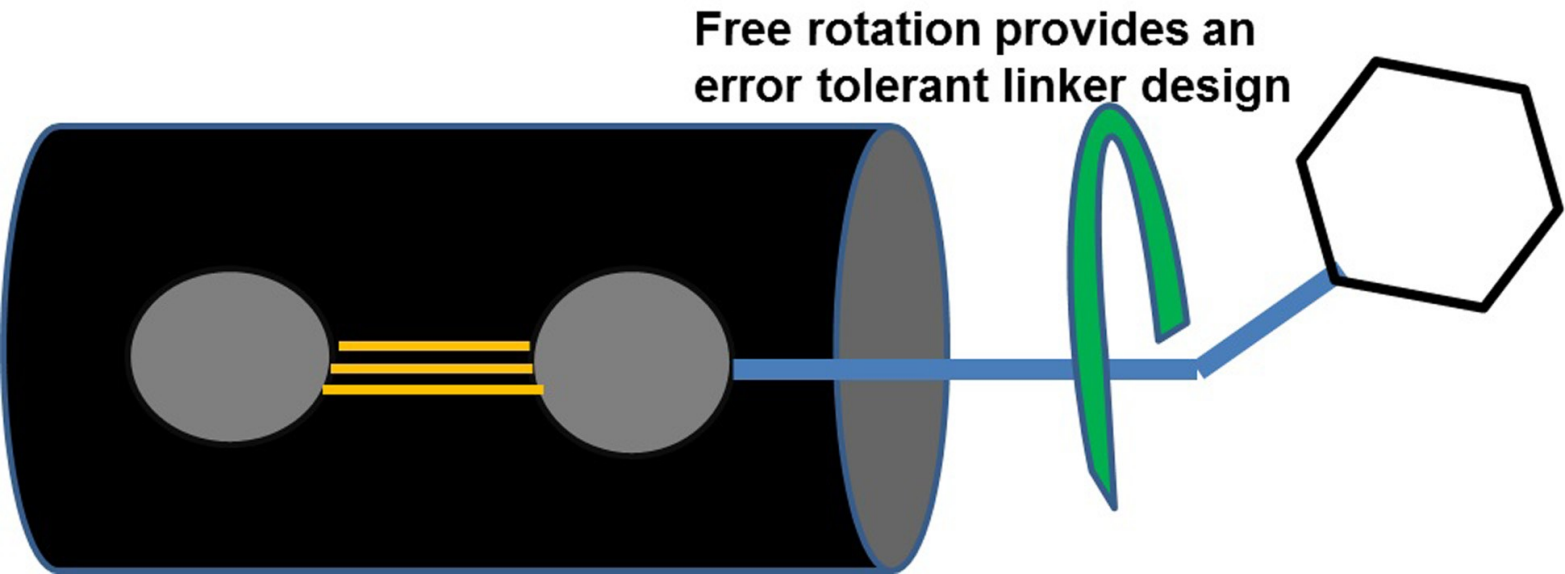
Direct single bond linkage to a triple bond enables penalty-free complete 360° dihedral rotation. A group terminally attached to an acetylene linker can freely align to match the contours of PCSK9 in this region and simultaneously present polar hydrogen to PCSK9 D374 to pincer this key residue.

Subtle changes can occur when linking or merging fragments, so whenever possible an element of flexibility is introduced. This minimizes the energy penalty for a dihedral rotation needed to accommodate hidden features of the protein’s surface. The added flexibility is kept to a minimum in order to prevent entropy penalties that can occur with overly flexible molecules. We systematically analyzed the fragment patterns to see if merging can be accomplished by directly linking a single bond to the triple bond. The reason for this is that the π-system of a triple bond has cylindrical orbital symmetry (Fig 10), which means that a functional group is free to spin into any orientation over the entire 360° dihedral circle. So, a fragment properly equipped with a polar proton linked to acetylene can adopt any orientation that makes it most compatible with the PCSK9 surface at this point, and also interacts with D374 completing its half of the pincer.

### No binding affinity at T377-R194-D238: The vast flat PPI surface

**Fig 11:**
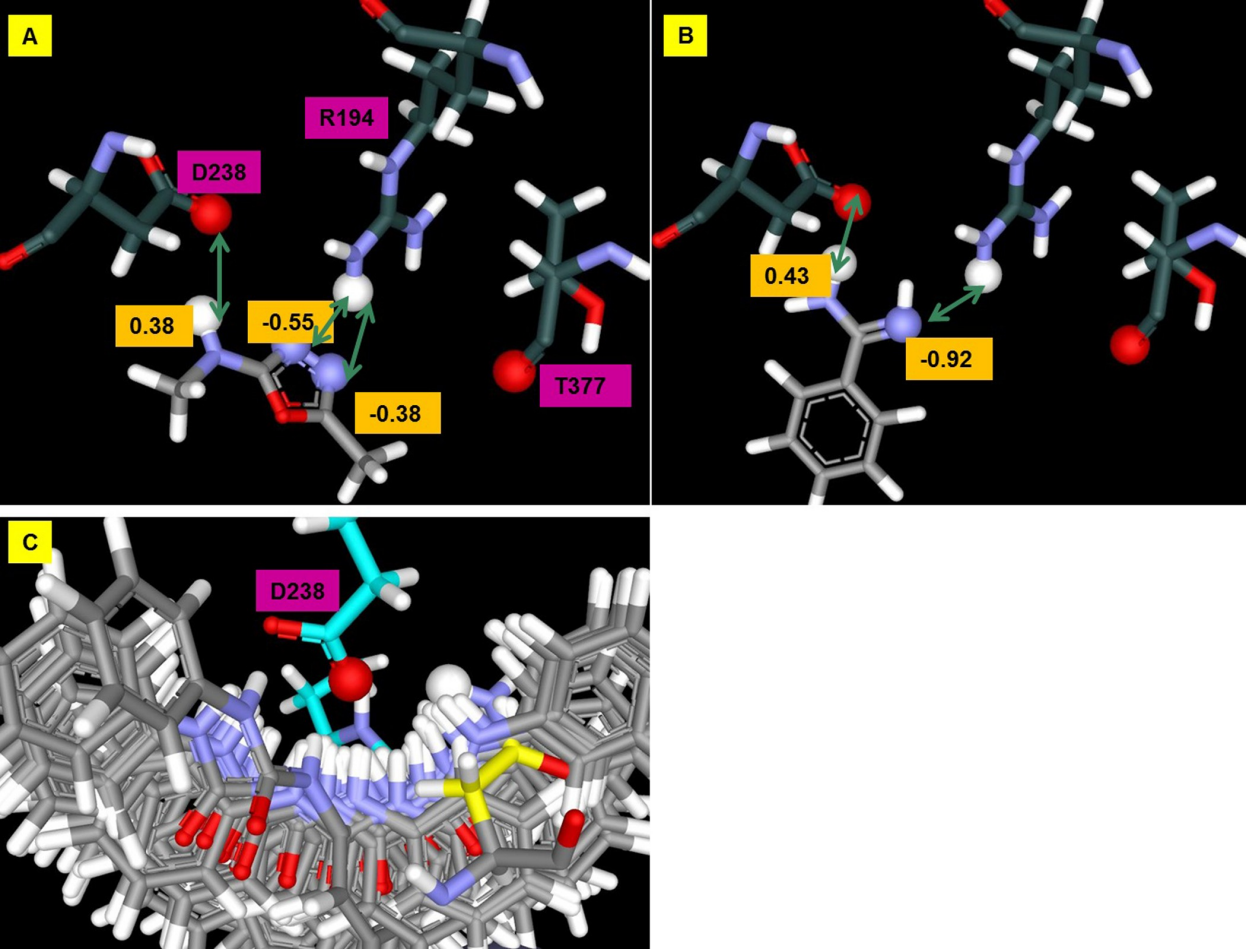
Much of the PCSK9 surface that interacts with LDLR is flat and featureless. Low affinity nondescript binding occurs with fragments like (A) dimethyl-oxadiazole and (B) benzamidine based on polar interactions (partial atomic charges shown in orange boxes) with R194-D238. The site is so highly exposed (C) that N,N’-diphenyl-urea can approach D238 from all directions.

SACP indicates that fragments bind with poor affinity at the T377-R194-D238 locus. There are no significant interactions at the T377 backbone site and only polar interactions (Fig 11A and 11B) at the D238-R194 locus that are not even sufficient to overcome the desolvation[67] penalty. The fact that this region is a vast featureless flat space can be seen in the Boltzmann distribution of N,N’-diphenyl-urea (Fig 11C) around D238 at weak chemical potential, which is uniformly distributed and thus all angles of approach are equally probable. It is very interesting that results of SACP at the T377-R194-D238 locus confirm well-established PPI findings–an extensive 3° structural contact surface is probably impossible to target for drug discovery, because it is too large and too featureless to support binding even by high molecular weight organic molecules. Therefore, the SACP simulations on PCSK9 indicate the high degree of difficulty in carrying out FBDD on this protein and thus any chance of achieving success requires tapping into special features of the PCSK9/LDLR interface, such as its partial covalent interaction character. Such considerations must be integrated into the design strategy.

## Results: Experimental

### Breaking the PCSK9/LDLR PPI with small molecules

**Fig 12:**
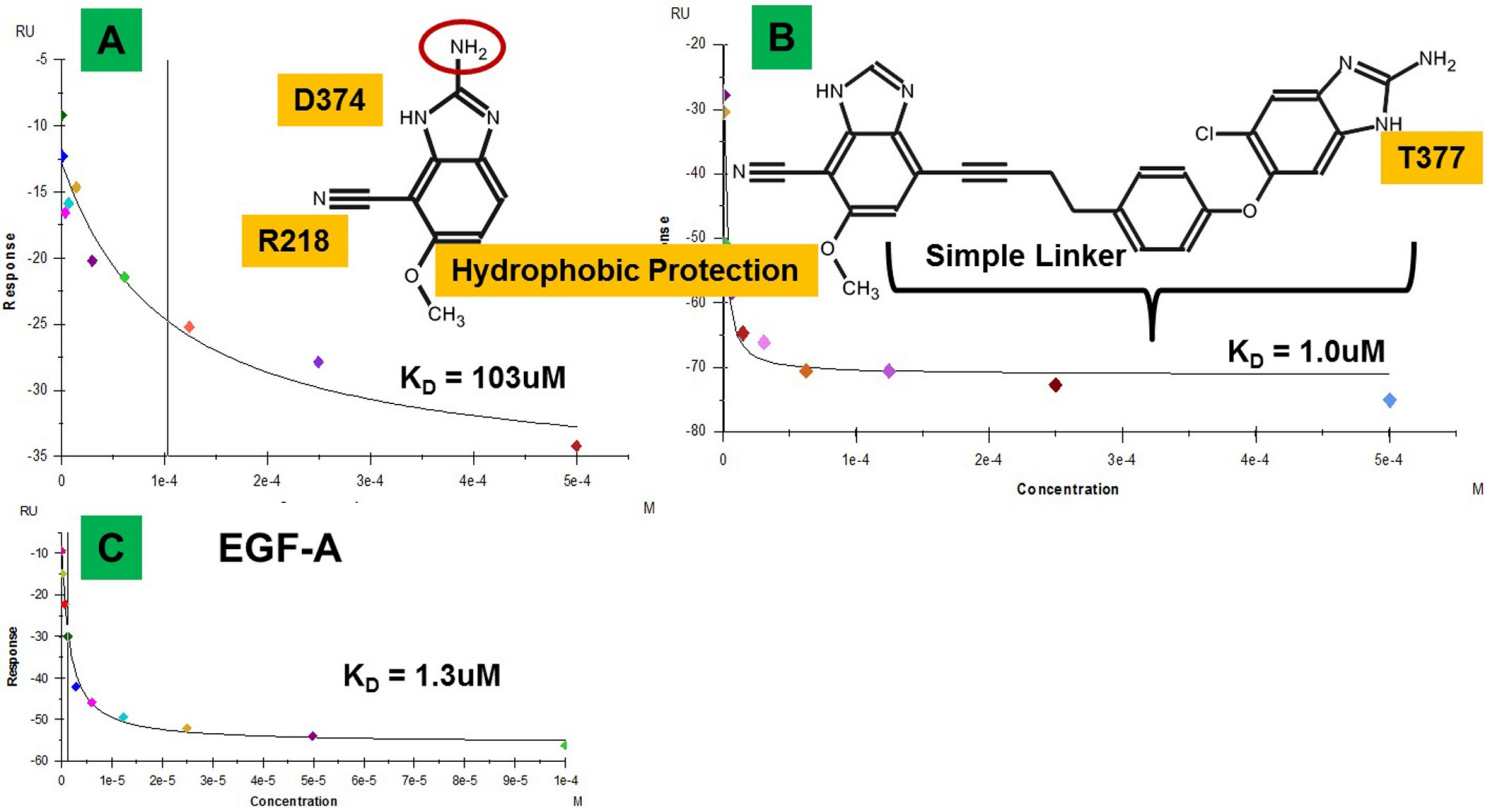
Surface plasmon resonance assay. The cyano-benzimidazole derivative (A) disrupts the PCSK9/LDLR PPI in SPR competition assays. There is no precedent, to our knowledge, for such a small fragment (MW ~ 200) breaking the PPI of a tertiary interface. The amine function circled in red is not relevant for the binding but was left on due to synthetic ease and because SACP indicates that there is free space in this location. The methoxy-ether group was added as hydrophobic protection for the polar interaction at R218 with the cyano group. A simple linker designed to not clash with PCSK9 was added to present the benzimidazole proton at T377 backbone to form the second interaction, resulting in a compound (B) that breaks the PPI with the same potency as the EGFa (C) protein domain of LDLR.

GAMESS/RESP calculations indicate that it is in fact possible to attain high affinity binding at the T377-R194-D238 locus with a small molecule by tapping into the intrinsic electron delocalization propensity from this site through the D374-R218 locus of PCSK9 that is seen upon binding to LDLR. To obtain support for this conjecture, it is necessary to show that increasing the potential for fractional charge transfer between PCSK9 and a small molecule found in modeling increases the binding affinity. Thus, we synthesized a cyano-benzimidazole derivative (Fig 12A) targeted to bind at the D374-R218 site and then added a linker with a benzimidazole (Fig 12B) targeted to hit the ketone backbone of T377. The linker was designed using methods described above for the explicit purpose of simply spanning the appropriate distance without causing any clashes with PCSK9. In the initial synthesis of the cyano-benzimidazole, an extraneous amine function was retained for synthetic ease and because SACP indicated that there was freedom at this position, so it should not interfere with binding. The methoxy group was added as hydrophobicity to help protect the strongly polar interaction at D374-R218 from solvation.

### PCSK9/LDLR competition binding with surface plasmon resonance (SPR)

PSCK9 has a range of interaction sites distributed throughout the protein, so a simple binding assay may not be relevant if the goal is to disrupt the PCSK9/LDLR PPI. TheEGFa domain of LDLR binds directly to PCSK9 (Figs 1 and 2) with 1.3μM affinity (Fig 12C) and is the standard used in competition assays.

This SPR assay protocol was developed at Merck[68] and they graciously agreed to test our compounds, which show that the cyano-benzimidazole disrupts the PPI at 103 μM and the compound that hits both sites is equipotent to EGFa. To summarize these results, a small fragment (MW ~ 200) binding at one localized site breaks an extensive complicated 3° PPI that spans over 20 Å and a small molecule that hits two sites has potency equivalent to the protein domain from its LDLR partner. We take this as possible support for fractional charge transfer and partial covalent binding, because, to our knowledge, there are no other examples in the literature for a fragment and a low molecular weight compound to have such potency against 3° PPI. Therefore, we must have tapped into something unique at the PCSK9/LDLR interface.

### Functional cell assays

**Fig 13:**
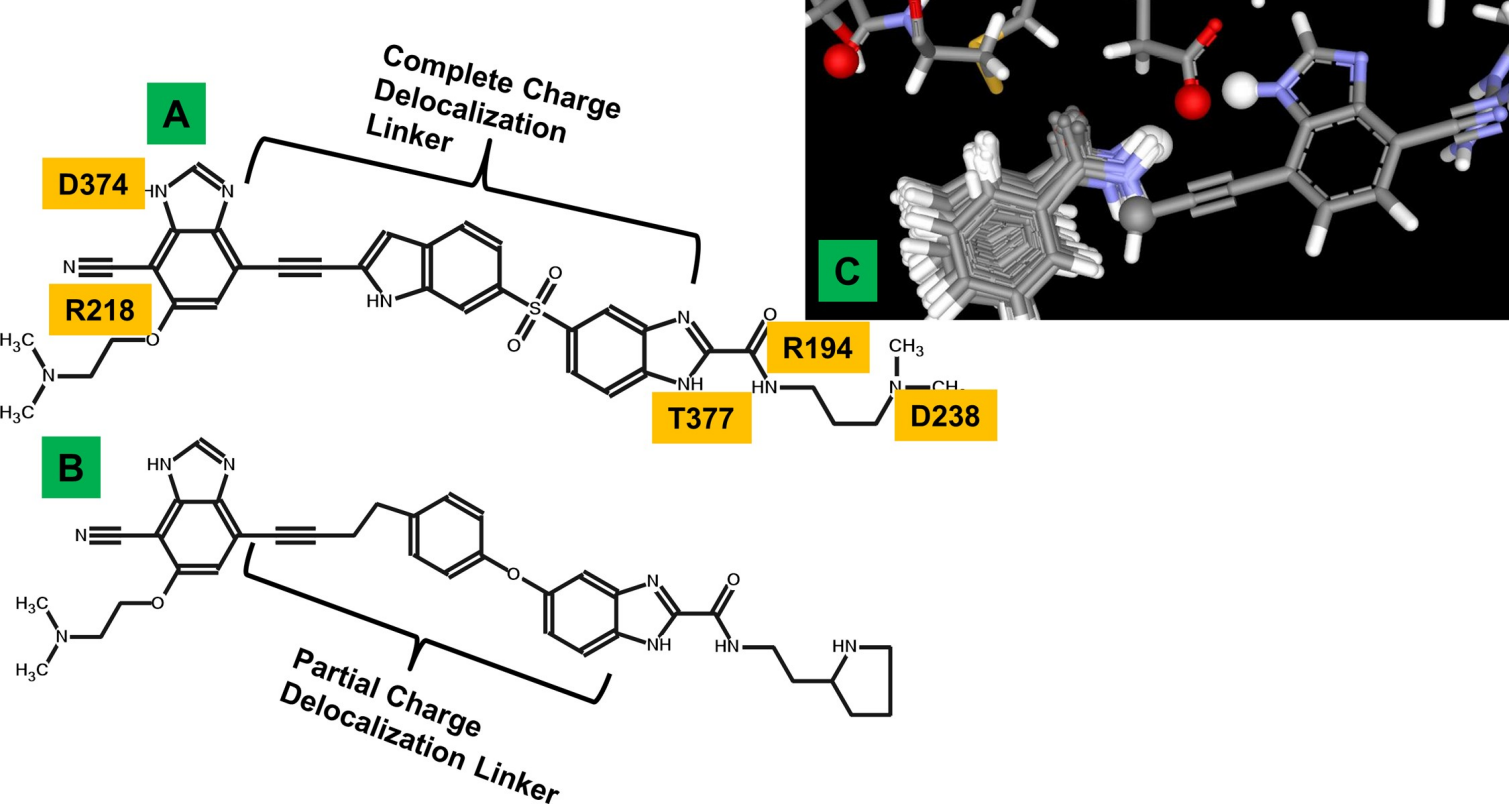
Complete vs. partial charge delocalization linkers. The molecule with a complete delocalization linker (A) was designed based on the benzaldehyde (C) and indole merging with acetylene predicted by SACP to also pincer D374. This linker strategy is based on connecting sites on the protein in a way that enables charge delocalization from D374 to R194. The partial delocalization linker (B) is designed to make the same interactions with PCSK as (A), so any difference in function provides evidence supporting our end-to-end charge delocalization conjecture.

**Fig 14:**
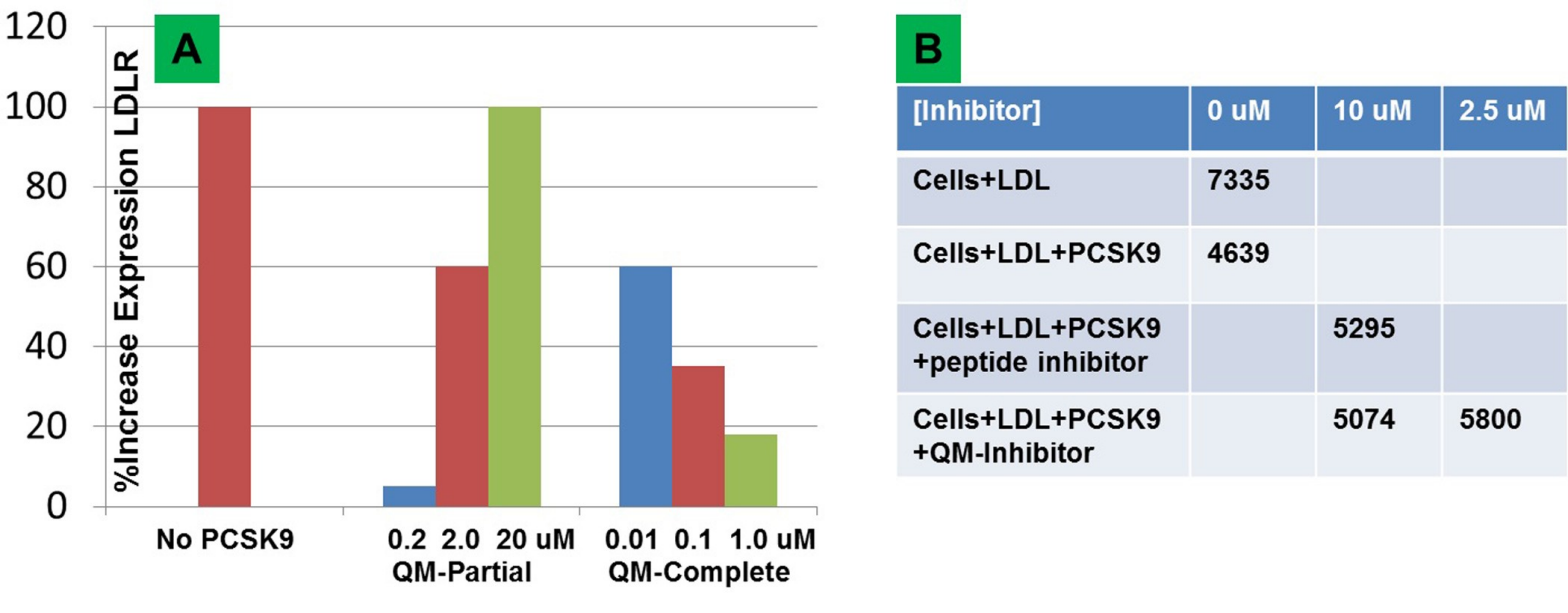
PCSK9 cellular assays. Addition of PCSK9 to HepG cells causes LDLR internalization. LDLR cell surface expression (A) is normalized by assigning 0% and 100% to the experiment with added and no PCSK9 respectively. Using this scale, the compound with the partial QM linker has an EC_50_ between 0.2–2.0 μM with a typical dose-response curve. The compound with a delocalization linker has an EC_50_ < 10 nM with an aberrant inverse dose-response curve (observed with anti-cancer agents, see discussion) due to π-π stacking. We tested the compound with a linker that supports delocalization (B) in fluorescently labeled LDL uptake assays with HepG cells. When the compound blocks PCSK9, more LDL particles are internalized inside the cells and the fluorescence remains after a wash. We used a peptide inhibitor known to block PCSK9 with 1 μM affinity as a positive control. The fluorescence of the cells after application of labeled LDL is 7335, but reduced to 4639 with application of PCSK9 and partially restored to 5295 when 10 μM of peptide is added. The complete inhibitor shows inverse dose-response in this assay also, becoming dramatically more potent when lowering the concentration from 10 μM to 2.5 μM. Japan Tobacco obtained almost identical results upon repeating the assays several times and so supplied us with the average values. We repeated our experiments six times and obtained very similar results in all cases.

We next designed two virtually identical compounds that target all four key interaction sites on PCSK9 (Fig 2), with the only difference being one has a linker that supports charge delocalization and the other only partially (Fig 13). Since both compounds make the exact same four interactions with PCSK9, this test aims to see the difference in potency as a function of increasing fractional charge transfer propensity. The sulfone linker (Fig 13A) spans the same distance as the ether linker (Fig 13B) with the added sp3 carbon. Thus the only difference is that the sulfone containing compound has a longer path of electron resonance, whereas the electron delocalization path in the ether containing compound is somewhat disrupted due to the sp3 carbon off the benzene ring. The expectation is that the sulfone compound should be significantly more potent than the ether compound, even though both are designed to make the exact same four contact interactions with PCSK9. These larger compounds are extensions of the smaller compounds discussed above, so we skipped the SPR binding studies and proceeded directly to biologically relevant cellular assays used to test PCSK9 inhibition: 1) cell surface expression of LDLR, and 2) uptake of fluorescently labeled LDL particles (Fig 14).

For the LDLR cell surface expression assays, which were graciously performed by Japan Tobacco, PCSK9 is mixed with the compound and added to HepG cells. On the following day an LDLR antibody is added to assess the level of receptor cell surface expression. The compound with a partial charge delocalization linker (Fig 14A) is fairly potent, enhancing LDLR cell surface expression with 0.2 < EC_50_ < 2.0 μM and a typical dose-response curve. The compound with the complete charge delocalization linker (Fig 14A) is two orders of magnitude more potent with EC_50_ < 10 nM and an inverse dose-response curve. This type of aberrant behavior, the compound becomes more potent as the concentration is decreased, is due to π-π stacking and has been observed and well-described by cancer researchers (see discussion). After getting these results from our collaborators, we conducted HepG LDL uptake studies. The assays use commercially available fluorescently labeled LDL particles, which are taken inside the cell by LDLR, so the fluorescence is protected from a wash, resulting in a fluorescence reading of 7,335. When PCSK9 is added with the LDL particles, the fluorescence is reduced to 4,639 after a wash, reflecting the lower amount of LDL cellular uptake. A PCSK9 inhibitor (peptide 2–8) reported to have an EC_50_ of 1 μM was used as a positive control–when it is added to the mixture of PCSK9-LDL, the fluorescence is increased to 5,295 showing that the peptide partially blocks PCSK9. Adding the compound with the complete charge delocalization linker restores fluorescence back to 5,074 so is seemingly less potent then peptide 2–8, but the fluorescence goes to 5,800 when the concentration is reduced to 2.5 μM. We have confirmed in a different assay that the compound with the charge delocalization linker has an inverse dose-response curve. This type of aberrant behavior is due to the π-π stacking and has been well-described in the cancer literature where many anti-neoplastic agents have extensive resonant planar structure, that are especially targeted at DNA intercalation. We give a brief summary of this in the discussion section. Nevertheless, the complete charge delocalization linker compound is approximately two orders of magnitude more potent than a virtually identical compound with a partial charge delocalization linker in biologically relevant functional assays. There is no way to explain this extreme divergence based on the interactions with PCSK9, because both compounds were designed to make the same number of identical non-polar and polar interactions with the protein. The only real difference between the two compounds is the degree of resonant electron delocalization, so the two order of magnitude difference in potency is most likely due to the difference in charge delocalization propensity between the ligand and protein.

## Discussion

Solubility is a key design concern with relatively planar molecules that contour the convex surface of the PPI interface. Extended π-systems induce compounds to self-aggregate due to non-bonded π-π stacking, leading to poor solubility and aberrant behavior. An important medically relevant example is the anti-cancer DNA intercalating agent doxorubicin, which must be dose-limited, because it causes cardiomyopathy. Golunski[69] and co-workers hypothesized that doxorubicin toxicity could be reduced without loss of efficacy by disrupting its π-π self-recognition with pentoxifylline (S2 Fig)–an aromatic molecule known to breakup π-π -stacking. Hartlieb[70] and colleagues attacked this problem differently—they showed that disruption of the π-stacking of 2,9-diazaperopyrenium by adding a functional group that causes a steric clash with the neighboring molecule, results in increased anti-cancer potency (S2 Fig). These investigators solved the crystal structures of the homo-aggregates and demonstrated that biological activity increased as stacking decreased. A nice example of unexpected π-stacking in protein-ligand binding was shown by Stornaiuolo[71] and co-workers; they did a fragment screening experiment on a soluble acetylcholine binding protein and discovered that large planar fragments aggregate in a triple π-stack (S2 Fig) at the protein active site.

Our most potent PCSK9 inhibitor has extended electron delocalization from the D374-R218 locus all the way to the R194 site, which is approximately 20 Å and thus this molecule, could self-aggregate due to π-π stacking. The simplest way to eliminate π-π stacking is by dilution and this is what our collaborators at Japan Tobacco did in their LDLR cell surface expression experiments, and what we did in our HepG cell LDL uptake work. At the higher concentrations, a large fraction of compound was unavailable to inhibit PCSK9, because it was sequestered in the self-assembled aggregate. Only at low doses below the self-recognition threshold, was the entire dose available to interact with PCSK9. This is exactly analogous to the anti-cancer DNA intercalating agents and π-π stacking is the reason for the inverse dose-response curves seen in both assays. We were fortunate that the compound is very potent, because if it had less affinity for PCSK9, it is likely that the compound would have been deemed inactive.

So while it is gratifying to have successfully designed PCSK9 inhibitors based on a charge delocalization and polarization hypothesis, it could be said that solving this difficult PPI challenge only resulted in being confronted with a new difficult challenge–how do we deal with the self-aggregation? Fortunately, the cancer community has made good progress developing[72–75] formulations specifically designed to address the π-π stacking problem, which are directly applicable to our PCK9 compounds.

## Conclusions

We have applied the SACP fragment simulation method to a multitude of different proteins. The defining characteristics of a binding site are its propensity to bind a wide diversity of fragments with high affinity and simultaneously have low affinity for water so there is no significant desolvation penalty. The large area of PCSK9 that can be called the LDLR Ca^2+^ ion site, does not bind any fragments with high affinity according to SACP, and is strongly hydrated according to solvation calculations. So, by any traditional measures, we would have immediately abandoned any efforts at small molecule design, because SACP clearly indicates that this is not a druggable site. We made a dedicated effort because of the vital importance of PCSK9 in hypercholesterolemia and heart disease. We continued molecular design work and laboratory experiments because the GAMESS/RESP calculations confirmed the special nature of the PCSK9/LDLR PPI, which makes it vulnerable to small molecule attack. The general conclusions from this work are, 1) it is difficult to impossible to break 3° PPI with high affinity using any conventional means of drug discovery such as HTS or FBDD, and 2) if the goal is to break a 3° PPI with a small molecule, then it is incumbent upon the investigators to determine a unique feature of the interaction that can be exploited. Finally, it is important to note that the Burgess[76] group successfully designed 20–40 μM small molecule PCSK9 inhibitors using the innovative computational procedure called Exploring Key Orientations, which creates scaffolds that present three side chains from the LDLR at critical interaction points with PCSK9. While these results are impressive, it confirms the great difficulty in breaking 3° PPI, because even achieving inhibition >20 μM requires careful design of a molecule the size of a tripeptide. Using SACP to discover a fragment with a molecular weight ~202 Da that could break the PCSK9/LDLR at 100 μM and small molecules with nanomolar functional potency was only possible by carefully studying the charge delocalization properties of this PPI.

## Methods

### Computational

#### Selecting the atoms at the PCSK9/LDLR interface

The PCSK9/LDLR complex from PDB 3GCW or PDB 3BPS was loaded into the BioLeap in-house protein modeling package called BFD. Each atom at the protein-protein interface was manually selected and put into a working set. The complete set of atoms that make up the PCSK9/LDLR interface was saved as a PDB file. The goal was to be as comprehensive as possible, including all of the most important interactions, but keeping the total number of heavy atoms to a maximum of 520 in order to ensure that the calculation was feasible in a practical amount of time. In our initial test GAMESS/RESP calculations indicated that ~500 atoms was an upper bound for a practical calculation–one that takes about 2–3 weeks (in 2012). Some of the calculations were somewhat larger, about 520 atoms, which was necessary for inclusion of important interactions.

#### GAMESS/RESP calculations

Residue collections from the designated protein X-ray structure with or without ligands with non-bonded interactions, were selected using in-house BFD fragment-based design tool. This tool fills in adjacent residues (i.e. if residues 101 and 103 are requested, residue 102 will be added), and then caps the ends (ACE added to N-terminals, NMe added to C-terminals). The final residue complement is written to a PDB-format file and submitted to an in-house web service for running the GAMESS program. The result is a grid of electrostatic field values (derived from electron densities) which is passed to the RESP program (part of the Amber antechamber tool set) to fit partial charges at atom centers to the fields. The result is written in a BioLeap .fdb format file which can be used in SACP simulations.

Next, the terminal ACE/NME groups are removed, and the atom charges for those groups are merged into the charges for the adjacent residues so that the total charge doesn’t change. Although this charge merging isn’t perfect, the variance from more complete calculations including adjacent residues is small. If not, the adjacent residues should be included. The ACE CH3, C, and O atom charges are merged with the CA, C, O atom charges of the preceding residue, and HH31, HH32, HH33 charges are merged with the HA atom. The NME CH3, N atom charges are merged with CA, N atom charges of the succeeding residue, and HH31, HH32, HH33 are merged with H. The resulting residues with modified charges are written into a new .fdb format file. An example input file is included in the supporting information.

Here, the quantum mechanical calculations were run using the General Atomic and Molecular Electronic Structure System (GAMESS),[45] which is a general quantum chemistry package. GAMESS was run in 2 passes with different basis sets. The Restricted Hartree Fock wavefunctions and grid-based DFT with B3LYP functionals (BECKE exchange + LYP correlation) were used. This is a hybrid method combining five functionals: Becke + Slater + HF exchange (B3), with LYP + VWN5 correlation. The first pass starts with a Huckel guess (carry out an extended Huckel calculation using a Huzinaga 3 gaussian minimal basis set (MINI), followed by SCF (Self-consistent Field) calculations with the MINI basis. The maximum number of SCF iteration cycles was 200. In the second pass, eigenvectors from converged SCF calculation of the first pass are used to project the MINI orbitals onto the 3-N-G21 basis set (Pople's N-21G split valence basis set). Connolly was set to determine the points at which to compute the electrostatic potential. The complete list of GAMESS input parameters can be found in the supporting information (S3 Fig).

#### Simulated annealing of chemical potential

Comprehensive details on how these simulations are performed were described recently,[51] which illustrates the method in a multiplicity of examples.

### Experimental

#### Surface plasmon resonance

The PCSK9 group at Merck, which developed the SPR protocol for testing small molecule binding to PCSK9 in competition with the EGF domain from LDLR, graciously agreed to perform SPR experiments on our compounds. More complete details of the method were described previously.[77]

#### LDL particle uptake by HepG cells

Monitoring the HepG cell uptake of fluorescently labeled LDL particles is a standard assay used to measure the functional activity of LDLR and the associated inhibition of this receptor by PCSK. The interested reader may obtain complete details from the Genetech[78] paper. An outline of the protocol used in this study is given as follows.

Human hepatocyte-derived HepG2 cells were obtained from ATCC and maintained in Dulbecco modified Eagle medium and F-12 medium (DMEM/F12) (Invitrogen) supplemented with 10% fetal bovine serum, 100 U of penicillin/ml, and 100 μg of streptomycin/ml. HepG2 cells were seed at 30,000 cells/well. Cells were incubated overnight in a 96-well plate. Cells were washed 3X with 200 μl PBS/well. Media was changed to 200 μl RPMI medium with (10%) lipoprotein deficient serum. After 24 hours Pep2-8 or each compound, at various concentrations, were preincubated with 15 μg/mL of PCSK9 in 0.5% DMSO for 30 minutes prior to the addition to the cells. PCSK9 was obtained from Emerald Life Sciences. After 1.5 hours, 10 μg/mL BODIPY-LDL (Invitrogen) was added and the mixture was incubated for 3.5 hours. Cells were washed three times with PBS, and fluorescence was measure. Excitation was set to 495 nm and emission to 512 nm. All experiments were done in triplicate.

#### LDLR HepG cell surface expression

The PCSK9 group at Japan Tobacco graciously agreed to perform the LDLR HepG cell surface expression assay using standards protocols that are described in a paper by the Merck group[79]. An outline of the protocol used in this study is given as follows.

#### Preparation of the test articles

The stock solutions of the test articles were serially diluted with DMSO. These solutions were diluted 250-fold with DMEM containing 0.2% BSA and PCSK9 protein was added to the solutions. These solutions were used as the test article solutions (final concentration of PCSK9 was 10 μg/mL).

#### Preparation of the reference article

The stock solution of the reference article (20 mmol/L) was diluted with DMSO to the concentration of 7.5 mmol/L. This solution was diluted 250-fold with DMEM containing 0.2% BSA and PCSK9 protein was added to the solution. This solution was used as the reference article solution (final concentrations of the reference article and PCSK9 were 30 μmol/L and 10 μg/mL, respectively).

#### Preparation of the vehicle solution and the blank solution

DMSO was diluted 250-fold with DMEM containing 0.2% BSA. The solution mixed with PCSK9 protein (final concentration was 10 μg/mL) was used as the vehicle solution and the solution without PCSK9 protein was used as the blank solution.

#### Cell seeding and treatment of compounds

HepG2 cells were seeded onto Collagen-I coated 48-well culture plate at a density of 1.6×10^5^ cells/well and cultured in DMEM containing 10% FBS (300 μL/well) overnight at 37°C in 5% CO_2_. The medium was removed from each culture well and the cells were washed two times with PBS. Then, the vehicle solution, the blank solution, each test article solution or the reference solution (300 μL/well) was added successively to each well and cultured for 24 h at 37°C in 5% CO_2_.

#### Measurement of the LDLR protein

The medium was removed from each culture well. The cells were washed two times with PBS, then 150 μL of cell lysis buffer was added to each well and incubated on the plate shaker for 2 minutes at room temperature. Then, cell lysate was collected to another 96-well microplate and centrifuged at 4°C for 10 minutes. The supernatants were collected and the concentration of LDLR protein in the supernatants was measured by Human LDLR ELISA kit according to the kit instruction (R&D Systems). Also, the concentration of cellular protein in the supernatants was measured by BCA Protein Assay Reagents (Thermo Scientific). The concentration of LDLR protein was corrected by the concentration of cellular protein in each well. By defining the corrected value of the vehicle as 100% of LDLR protein and the corrected value of the blank as 0%, percent.

#### Chemical synthesis

The synthetic protocols used to create the molecules used in this study are described in the supplementary materials (S4 Fig).

## Supporting information

S1 Table List of standard AMBER charges and custom derived charges for PCSK9-LDLR. (DOCX)

S2 Table List of fragments run on PCSK9. (DOCX)

S3 Table List of standard AMBER charges and custom charges for the CN-benzimidazole fragment bound to PCSK9. (DOCX)

S1 Fig Ball-and-stick representation of the connected path of interpenetrating atoms. (DOCX)

S2 Fig Examples of π-π stacking. (DOCX)

S3 Fig GAMESS input parameters. (DOCX)

S4 Fig Synthetic schemes for fragments and compounds. (DOCX)
