# Supplementary material for: Fragment-based design of small molecule PCSK9 inhibitors using simulated annealing of chemical potential simulations
Source: PLoS One. 2019 Dec 5;14(12):e0225780. doi: 10.1371/journal.pone.0225780 (PMC6894869; doi:10.1371/journal.pone.0225780)
Supplement: S1 Table — (DOCX) [file pone.0225780.s001.docx]

**Supporting Information**

**Designing Small Molecule PCSK9 Inhibitors Guided by Simulated Annealing of Chemical Potential Simulations**

*Frank Guarnieri^1,2^, John L. Kulp Jr.^3^, John L. Kulp III^3,4^, Ian S. Cloudsdale^3^

^1^Center for Drug Discovery, Northeastern University, Boston, MA 02115 USA

^2^PAKA Pulmonary Pharmaceuticals, Acton, MA 01720 USA

^3^Conifer Point Pharmaceuticals, Doylestown, PA 18902 USA

^4^Department of Chemistry, Baruch S. Blumberg Institute, Doylestown, PA 18902 USA

*Corresponding author

Email: [frankguarnieri@yahoo.com](mailto:frankguarnieri@yahoo.com)

**Contents**

1. S1 Table. List of standard AMBER charges and custom derived charges for PCSK9-LDLR
2. S2 Table. List of fragments run on PCSK9
3. S3 Table. List of standard AMBER charges and custom charges for the CN-benzimidazole fragment bound to PCSK9
4. S1 Fig. Ball-and-stick representation of the connected path of interpenetrating atoms.
5. S2 Fig. Examples of π-π stacking.
6. S3 Fig. GAMESS input parameters
7. S4 Fig. Synthetic schemes for fragments and compounds

**S1 Table**. List of standard AMBER charges and custom derived charges for PCSK9-LDLR.

| **Residue/Atoms** | **PCSK9 Chain A only** | **LDL-R Chain E** | **PCSK9 Chain A & LDL-R Chain E** | **PCSK9 Chain A & LDL-R H306Y Chain E** | **Amino Acid Dielectric 1** | **Amino Acid Amber Charge** |
| --- | --- | --- | --- | --- | --- | --- |
| PCSK9 Chain A |  |  |  |  |  |  |
| ARG 194 |  |  |  |  |  |  |
| Total charge | *0.655* |  | *0.447* | 0.416 | *1.000* | *1.000* |
| Side-chain charge | *0.954* |  | *0.854* | 0.793 | *0.983* | *0.928* |
| Backbone charge | *-0.299* |  | *-0.408* | -0.377 | *0.017* | *0.072* |
| N | *-0.279* |  | *-0.231* | -0.243 | *-0.359* | *-0.348* |
| CA | *-0.085* |  | *-0.043* | -0.078 | *0.119* | *-0.264* |
| C | *0.321* |  | *0.218* | 0.125 | *0.652* | *0.734* |
| O | *-0.468* |  | *-0.467* | -0.375 | *-0.535* | *-0.589* |
| CB | *0.056* |  | *0.073* | 0.006 | *-0.230* | *-0.001* |
| CG | *0.005* |  | *-0.028* | -0.084 | *-0.252* | *0.039* |
| CD | *-0.153* |  | *-0.355* | -0.241 | *0.514* | *0.049* |
| NE | *-0.385* |  | *-0.030* | -0.132 | *-0.741* | *-0.529* |
| CZ | *0.589* |  | *0.060* | 0.011 | *0.749* | *0.808* |
| NH1 | *-0.746* |  | *-0.424* | -0.311 | *-0.876* | *-0.863* |
| NH2 | *-0.746* |  | *-0.424* | -0.311 | *-0.876* | *-0.863* |
| H | *0.127* |  | *0.073* | 0.117 | *0.259* | *0.275* |
| HA | *0.103* |  | *0.097* | 0.122 | *-0.052* | *0.156* |
| HB2 | *0.000* |  | *0.007* | 0.031 | *0.104* | *0.033* |
| HB3 | *0.000* |  | *0.007* | 0.031 | *0.104* | *0.033* |
| HG2 | *0.047* |  | *0.052* | 0.058 | *0.112* | *0.029* |
| HG3 | *0.047* |  | *0.052* | 0.058 | *0.112* | *0.029* |
| HD2 | *0.075* |  | *0.137* | 0.133 | *0.006* | *0.069* |
| HD3 | *0.075* |  | *0.137* | 0.133 | *0.006* | *0.069* |
| HE | *0.387* |  | *0.294* | 0.326 | *0.385* | *0.346* |
| HH11 | *0.421* |  | *0.310* | 0.260 | *0.450* | *0.448* |
| HH12 | *0.421* |  | *0.310* | 0.260 | *0.450* | *0.448* |
| HH21 | *0.421* |  | *0.310* | 0.260 | *0.450* | *0.448* |
| HH22 | *0.421* |  | *0.310* | 0.260 | *0.450* | *0.448* |
|  |  |  |  |  |  |  |
| GLU 195 |  |  |  |  |  |  |
| Total charge | *-0.422* |  | *-0.465* | -0.360 | *-1.000* | *-1.000* |
| Side-chain charge | *-0.618* |  | *-0.643* | -0.536 | *-0.888* | *-0.732* |
| Backbone charge | *0.197* |  | *0.178* | 0.176 | *-0.112* | *-0.268* |
| N | *-0.083* |  | *-0.010* | 0.003 | *-0.610* | *-0.516* |
| CA | *-0.071* |  | *-0.013* | 0.019 | *-0.075* | *0.040* |
| C | *0.428* |  | *0.382* | 0.285 | *0.624* | *0.537* |
| O | *-0.336* |  | *-0.362* | -0.284 | *-0.587* | *-0.582* |
| CB | *-0.314* |  | *-0.203* | -0.218 | *0.029* | *0.056* |
| CG | *-0.089* |  | *-0.190* | -0.246 | *-0.428* | *0.014* |
| CD | *0.560* |  | *0.494* | 0.585 | *0.913* | *0.805* |
| OE1 | *-0.556* |  | *-0.571* | -0.569 | *-0.793* | *-0.819* |
| OE2 | *-0.556* |  | *-0.571* | -0.569 | *-0.793* | *-0.819* |
| H | *0.188* |  | *0.166* | 0.173 | *0.460* | *0.294* |
| HA | *0.081* |  | *0.067* | 0.106 | *0.055* | *0.111* |
| HB2 | *0.132* |  | *0.102* | 0.114 | *0.008* | *-0.017* |
| HB3 | *0.132* |  | *0.102* | 0.114 | *0.008* | *-0.017* |
| HG2 | *0.031* |  | *0.070* | 0.063 | *0.095* | *-0.043* |
| HG3 | *0.031* |  | *0.070* | 0.063 | *0.095* | *-0.043* |
|  |  |  |  |  |  |  |
| SER 221 |  |  |  |  |  |  |
| Total charge | *-0.024* |  | *-0.077* | -0.042 | *0.000* | *0.000* |
| Side-chain charge | *0.254* |  | *0.266* | 0.247 | *-0.036* | *0.114* |
| Backbone charge | *-0.278* |  | *-0.343* | -0.289 | *0.036* | *-0.114* |
| N | *-0.511* |  | *-0.426* | -0.484 | *-0.429* | *-0.416* |
| CA | *0.044* |  | *0.039* | 0.027 | *-0.154* | *-0.025* |
| C | *0.331* |  | *0.143* | 0.232 | *0.614* | *0.597* |
| O | *-0.424* |  | *-0.354* | -0.364 | *-0.489* | *-0.568* |
| CB | *0.189* |  | *0.070* | 0.097 | *0.285* | *0.212* |
| OG | *-0.634* |  | *-0.634* | -0.556 | *-0.725* | *-0.655* |
| H | *0.327* |  | *0.293* | 0.327 | *0.340* | *0.272* |
| HA | *0.153* |  | *0.163* | 0.251 | *0.099* | *0.084* |
| HB2 | *0.046* |  | *0.091* | -0.002 | *0.002* | *0.035* |
| HB3 | *0.046* |  | *0.091* | -0.002 | *0.002* | *0.035* |
| HG | *0.411* |  | *0.447* | 0.432 | *0.453* | *0.427* |
|  |  |  |  |  |  |  |
| LYS 222 |  |  |  |  |  |  |
| Total charge | *0.989* |  | *1.074* | 1.059 | *1.000* | *1.000* |
| Side-chain charge | *1.005* |  | *1.017* | 0.944 | *0.902* | *0.928* |
| Backbone charge | *-0.016* |  | *0.057* | 0.115 | *0.098* | *0.072* |
| N | *-0.342* |  | *-0.133* | -0.151 | *-0.375* | *-0.348* |
| CA | *-0.050* |  | *-0.029* | -0.053 | *-0.286* | *-0.240* |
| C | *0.558* |  | *0.480* | 0.542 | *0.655* | *0.734* |
| O | *-0.488* |  | *-0.473* | -0.470 | *-0.525* | *-0.589* |
| CB | *-0.276* |  | *-0.240* | 0.041 | *-0.082* | *-0.009* |
| CG | *-0.031* |  | *0.012* | 0.051 | *0.375* | *0.019* |
| CD | *-0.126* |  | *-0.052* | -0.026 | *0.143* | *-0.048* |
| CE | *0.131* |  | *0.083* | -0.033 | *-0.039* | *-0.014* |
| NZ | *-0.549* |  | *-0.427* | -0.279 | *-0.364* | *-0.385* |
| H | *0.256* |  | *0.183* | 0.194 | *0.343* | *0.275* |
| HA | *0.134* |  | *0.106* | 0.072 | *0.166* | *0.143* |
| HB2 | *0.099* |  | *0.084* | -0.010 | *0.044* | *0.036* |
| HB3 | *0.099* |  | *0.084* | -0.010 | *0.044* | *0.036* |
| HG2 | *0.082* |  | *0.059* | -0.032 | *-0.130* | *0.010* |
| HG3 | *0.082* |  | *0.059* | -0.032 | *-0.130* | *0.010* |
| HD2 | *0.061* |  | *0.039* | 0.035 | *-0.019* | *0.062* |
| HD3 | *0.061* |  | *0.039* | 0.035 | *-0.019* | *0.062* |
| HE2 | *0.077* |  | *0.084* | 0.113 | *0.106* | *0.114* |
| HE3 | *0.077* |  | *0.084* | 0.113 | *0.106* | *0.114* |
| HZ1 | *0.378* |  | *0.345* | 0.320 | *0.329* | *0.340* |
| HZ2 | *0.378* |  | *0.345* | 0.320 | *0.329* | *0.340* |
| HZ3 | *0.378* |  | *0.345* | 0.320 | *0.329* | *0.340* |
|  |  |  |  |  |  |  |
| ARG 237 |  |  |  |  |  |  |
| Total charge | *0.488* |  | *0.442* | 0.353 | *1.000* | *1.000* |
| Side-chain charge | *1.001* |  | *0.986* | 0.936 | *0.983* | *0.928* |
| Backbone charge | *-0.513* |  | *-0.545* | -0.583 | *0.017* | *0.072* |
| N | *-0.182* |  | *-0.172* | -0.061 | *-0.359* | *-0.348* |
| CA | *0.032* |  | *-0.001* | 0.006 | *0.119* | *-0.264* |
| C | *0.339* |  | *0.229* | 0.251 | *0.652* | *0.734* |
| O | *-0.517* |  | *-0.517* | -0.487 | *-0.535* | *-0.589* |
| CB | *0.069* |  | *0.028* | 0.021 | *-0.230* | *-0.001* |
| CG | *-0.073* |  | *-0.004* | -0.034 | *-0.252* | *0.039* |
| CD | *-0.109* |  | *-0.217* | -0.101 | *0.514* | *0.049* |
| NE | *-0.263* |  | *-0.098* | -0.101 | *-0.741* | *-0.529* |
| CZ | *0.422* |  | *0.098* | 0.315 | *0.749* | *0.808* |
| NH1 | *-0.629* |  | *-0.502* | -0.579 | *-0.876* | *-0.863* |
| NH2 | *-0.629* |  | *-0.502* | -0.579 | *-0.876* | *-0.863* |
| H | *-0.152* |  | *-0.085* | -0.286 | *0.259* | *0.275* |
| HA | *0.045* |  | *0.059* | 0.064 | *-0.052* | *0.156* |
| HB2 | *0.051* |  | *0.056* | 0.043 | *0.104* | *0.033* |
| HB3 | *0.051* |  | *0.056* | 0.043 | *0.104* | *0.033* |
| HG2 | *0.054* |  | *0.042* | 0.049 | *0.112* | *0.029* |
| HG3 | *0.054* |  | *0.042* | 0.049 | *0.112* | *0.029* |
| HD2 | *0.086* |  | *0.113* | 0.070 | *0.006* | *0.069* |
| HD3 | *0.086* |  | *0.113* | 0.070 | *0.006* | *0.069* |
| HE | *0.318* |  | *0.319* | 0.210 | *0.385* | *0.346* |
| HH11 | *0.358* |  | *0.345* | 0.348 | *0.450* | *0.448* |
| HH12 | *0.358* |  | *0.345* | 0.348 | *0.450* | *0.448* |
| HH21 | *0.358* |  | *0.345* | 0.348 | *0.450* | *0.448* |
| HH22 | *0.358* |  | *0.345* | 0.348 | *0.450* | *0.448* |
|  |  |  |  |  |  |  |
| ASP 238 |  |  |  |  |  |  |
| Total charge | *-0.865* |  | *-0.627* | -0.656 | *-1.000* | *-1.000* |
| Side-chain charge | *-0.756* |  | *-0.716* | -0.637 | *-0.691* | *-0.732* |
| Backbone charge | *-0.109* |  | *0.089* | -0.019 | *-0.309* | *-0.268* |
| N | *-0.001* |  | *0.106* | 0.023 | *-0.461* | *-0.516* |
| CA | *-0.001* |  | *0.046* | 0.046 | *0.029* | *0.038* |
| C | *0.443* |  | *0.435* | 0.314 | *0.453* | *0.537* |
| O | *-0.529* |  | *-0.540* | -0.491 | *-0.509* | *-0.582* |
| CB | *-0.185* |  | *-0.104* | -0.052 | *-0.116* | *-0.030* |
| CG | *0.658* |  | *0.557* | 0.467 | *0.743* | *0.799* |
| OD1 | *-0.704* |  | *-0.678* | -0.596 | *-0.706* | *-0.801* |
| OD2 | *-0.704* |  | *-0.678* | -0.596 | *-0.706* | *-0.801* |
| H | *-0.021* |  | *0.090* | 0.136 | *0.207* | *0.294* |
| HA | *0.092* |  | *0.071* | 0.075 | *0.081* | *0.088* |
| HB2 | *0.044* |  | *0.034* | 0.009 | *-0.008* | *-0.012* |
| HB3 | *0.044* |  | *0.034* | 0.009 | *-0.008* | *-0.012* |
|  |  |  |  |  |  |  |
| SER 373 |  |  |  |  |  |  |
| Total charge | *-0.147* |  | *-0.235* | -0.255 | *0.000* | *0.000* |
| Side-chain charge | *0.062* |  | *0.054* | 0.085 | *-0.036* | *0.114* |
| Backbone charge | *-0.209* |  | *-0.290* | -0.340 | *0.036* | *-0.114* |
| N | *-0.461* |  | *-0.419* | -0.402 | *-0.429* | *-0.416* |
| CA | *-0.042* |  | *-0.029* | -0.057 | *-0.154* | *-0.025* |
| C | *0.406* |  | *0.250* | 0.172 | *0.614* | *0.597* |
| O | *-0.487* |  | *-0.456* | -0.406 | *-0.489* | *-0.568* |
| CB | *0.115* |  | *-0.004* | 0.057 | *0.285* | *0.212* |
| OG | *-0.612* |  | *-0.611* | -0.578 | *-0.725* | *-0.655* |
| H | *0.333* |  | *0.336* | 0.296 | *0.340* | *0.272* |
| HA | *0.104* |  | *0.125* | 0.131 | *0.099* | *0.084* |
| HB2 | *0.041* |  | *0.079* | 0.067 | *0.002* | *0.035* |
| HB3 | *0.041* |  | *0.079* | 0.067 | *0.002* | *0.035* |
| HG | *0.416* |  | *0.415* | 0.398 | *0.453* | *0.427* |
|  |  |  |  |  |  |  |
| ASP 374 |  |  |  |  |  |  |
| Total charge | *-0.851* |  | *-0.799* | -0.720 | *-1.000* | *-1.000* |
| Side-chain charge | *-0.675* |  | *-0.608* | -0.585 | *-0.691* | *-0.732* |
| Backbone charge | *-0.177* |  | *-0.191* | -0.134 | *-0.309* | *-0.268* |
| N | *-0.283* |  | *-0.099* | -0.118 | *-0.461* | *-0.516* |
| CA | *0.032* |  | *-0.010* | 0.021 | *0.029* | *0.038* |
| C | *0.310* |  | *0.164* | 0.212 | *0.453* | *0.537* |
| O | *-0.480* |  | *-0.476* | -0.465 | *-0.509* | *-0.582* |
| CB | *-0.373* |  | *-0.067* | -0.044 | *-0.116* | *-0.030* |
| CG | *0.708* |  | *0.598* | 0.537 | *0.743* | *0.799* |
| OD1 | *-0.692* |  | *-0.699* | -0.616 | *-0.706* | *-0.801* |
| OD2 | *-0.692* |  | *-0.699* | -0.616 | *-0.706* | *-0.801* |
| H | *0.277* |  | *0.221* | 0.237 | *0.207* | *0.294* |
| HA | *0.127* |  | *0.130* | 0.074 | *0.081* | *0.088* |
| HB2 | *0.107* |  | *0.070* | 0.029 | *-0.008* | *-0.012* |
| HB3 | *0.107* |  | *0.070* | 0.029 | *-0.008* | *-0.012* |
|  |  |  |  |  |  |  |
| CYS 375 |  |  |  |  |  |  |
| Total charge | *0.067* |  | *0.094* | 0.084 | *0.000* | *0.000* |
| Side-chain charge | *-0.009* |  | *0.080* | -0.006 | *0.080* | *0.114* |
| Backbone charge | *0.076* |  | *0.014* | 0.091 | *-0.080* | *-0.114* |
| N | *-0.109* |  | *0.035* | -0.011 | *-0.463* | *-0.416* |
| CA | *-0.055* |  | *0.021* | -0.010 | *0.030* | *0.043* |
| C | *0.374* |  | *0.190* | 0.164 | *0.577* | *0.597* |
| O | *-0.323* |  | *-0.261* | -0.250 | *-0.510* | *-0.568* |
| CB | *-0.252* |  | *-0.275* | -0.257 | *-0.084* | *-0.079* |
| SG | *-0.148* |  | *-0.105* | -0.172 | *-0.138* | *-0.108* |
| H | *0.135* |  | *0.050* | 0.189 | *0.316* | *0.272* |
| HA | *0.098* |  | *0.096* | 0.086 | *0.068* | *0.077* |
| HB2 | *0.173* |  | *0.171* | 0.173 | *0.102* | *0.091* |
| HB3 | *0.173* |  | *0.171* | 0.173 | *0.102* | *0.091* |
|  |  |  |  |  |  |  |
| SER 376 |  |  |  |  |  |  |
| Total charge | *-0.014* |  | *-0.017* | 0.037 | *0.000* | *0.000* |
| Side-chain charge | *0.147* |  | *0.142* | 0.130 | *-0.036* | *0.114* |
| Backbone charge | *-0.161* |  | *-0.158* | -0.093 | *0.036* | *-0.114* |
| N | *-0.458* |  | *-0.282* | -0.248 | *-0.429* | *-0.416* |
| CA | *-0.025* |  | *0.008* | -0.037 | *-0.154* | *-0.025* |
| C | *0.331* |  | *0.192* | 0.213 | *0.614* | *0.597* |
| O | *-0.409* |  | *-0.381* | -0.364 | *-0.489* | *-0.568* |
| CB | *0.069* |  | *0.048* | -0.001 | *0.285* | *0.212* |
| OG | *-0.574* |  | *-0.575* | -0.511 | *-0.725* | *-0.655* |
| H | *0.375* |  | *0.313* | 0.306 | *0.340* | *0.272* |
| HA | *0.143* |  | *0.121* | 0.134 | *0.099* | *0.084* |
| HB2 | *0.058* |  | *0.059* | 0.074 | *0.002* | *0.035* |
| HB3 | *0.058* |  | *0.059* | 0.074 | *0.002* | *0.035* |
| HG | *0.419* |  | *0.421* | 0.397 | *0.453* | *0.427* |
|  |  |  |  |  |  |  |
| THR 377 |  |  |  |  |  |  |
| Total charge | *0.089* |  | *0.136* | 0.153 | *0.000* | *0.000* |
| Side-chain charge | *0.047* |  | *0.116* | 0.089 | *-0.357* | *0.114* |
| Backbone charge | *0.042* |  | *0.020* | 0.064 | *0.357* | *-0.114* |
| N | *-0.065* |  | *0.015* | 0.026 | *0.023* | *-0.416* |
| CA | *0.023* |  | *0.082* | 0.038 | *-0.786* | *-0.039* |
| C | *0.421* |  | *0.141* | 0.117 | *0.846* | *0.597* |
| O | *-0.498* |  | *-0.324* | -0.265 | *-0.633* | *-0.568* |
| CB | *0.208* |  | *0.163* | 0.051 | *0.526* | *0.365* |
| OG1 | *-0.627* |  | *-0.634* | -0.593 | *-0.687* | *-0.676* |
| CG2 | *-0.147* |  | *-0.225* | -0.088 | *-0.521* | *-0.244* |
| H | *0.185* |  | *0.187* | 0.186 | *0.122* | *0.272* |
| HA | *0.032* |  | *0.054* | 0.084 | *0.224* | *0.101* |
| HB | *0.008* |  | *0.046* | 0.093 | *0.053* | *0.004* |
| HG1 | *0.414* |  | *0.416* | 0.405 | *0.445* | *0.410* |
| HG21 | *0.045* |  | *0.072* | 0.033 | *0.129* | *0.064* |
| HG22 | *0.045* |  | *0.072* | 0.033 | *0.129* | *0.064* |
| HG23 | *0.045* |  | *0.072* | 0.033 | *0.129* | *0.064* |
|  |  |  |  |  |  |  |
| CYS 378 |  |  |  |  |  |  |
| Total charge | *0.035* |  | *0.100* | 0.051 | *0.000* | *0.000* |
| Side-chain charge | *0.060* |  | *0.075* | 0.031 | *0.080* | *0.114* |
| Backbone charge | *-0.025* |  | *0.025* | 0.020 | *-0.080* | *-0.114* |
| N | *-0.195* |  | *0.027* | 0.067 | *-0.463* | *-0.416* |
| CA | *-0.014* |  | *0.025* | 0.015 | *0.030* | *0.043* |
| C | *0.504* |  | *0.353* | 0.352 | *0.577* | *0.597* |
| O | *-0.460* |  | *-0.421* | -0.390 | *-0.510* | *-0.568* |
| CB | *0.100* |  | *-0.046* | -0.076 | *-0.084* | *-0.079* |
| SG | *-0.160* |  | *-0.171* | -0.167 | *-0.138* | *-0.108* |
| H | *0.126* |  | *0.065* | -0.009 | *0.316* | *0.272* |
| HA | *0.045* |  | *0.062* | 0.074 | *0.068* | *0.077* |
| HB2 | *0.045* |  | *0.102* | 0.093 | *0.102* | *0.091* |
| HB3 | *0.045* |  | *0.102* | 0.093 | *0.102* | *0.091* |
|  |  |  |  |  |  |  |
|  |  |  |  |  |  |  |
| LDL-R Chain E |  |  |  |  |  |  |
| Calcium ion |  |  |  |  |  |  |
| Total charge |  | *0.941* | *0.753* | 0.993 | *2.000* | *2.000* |
| CA |  | *0.941* | *0.753* | 0.993 | *2.000* | *2.000* |
|  |  |  |  |  |  |  |
| GLY 293 |  |  |  |  |  |  |
| Total charge |  | *-0.055* | *-0.055* | 0.034 | *0.000* | *0.000* |
| Side-chain charge |  | *0.151* | *0.136* | 0.164 | *0.113* | *0.114* |
| Backbone charge |  | *-0.205* | *-0.191* | -0.130 | *-0.113* | *-0.114* |
| N |  | *-0.367* | *-0.300* | -0.321 | *-0.503* | *-0.416* |
| CA |  | *-0.021* | *-0.024* | 0.059 | *0.112* | *-0.025* |
| C |  | *0.367* | *0.331* | 0.337 | *0.587* | *0.597* |
| O |  | *-0.545* | *-0.536* | -0.457 | *-0.578* | *-0.568* |
| H |  | *0.340* | *0.314* | 0.310 | *0.380* | *0.272* |
| HA2 |  | *0.086* | *0.080* | 0.052 | *0.000* | *0.070* |
| HA3 |  | *0.086* | *0.080* | 0.052 | *0.000* | *0.070* |
|  |  |  |  |  |  |  |
| THR 294 |  |  |  |  |  |  |
| Total charge |  | *0.093* | *0.138* | 0.025 | *0.000* | *0.000* |
| Side-chain charge |  | *0.192* | *0.189* | 0.094 | *-0.357* | *0.114* |
| Backbone charge |  | *-0.099* | *-0.052* | -0.068 | *0.357* | *-0.114* |
| N |  | *-0.046* | *-0.010* | -0.169 | *0.023* | *-0.416* |
| CA |  | *0.053* | *0.018* | -0.174 | *-0.786* | *-0.039* |
| C |  | *-0.016* | *-0.063* | 0.011 | *0.846* | *0.597* |
| O |  | *-0.160* | *-0.124* | -0.102 | *-0.633* | *-0.568* |
| CB |  | *0.190* | *0.071* | 0.191 | *0.526* | *0.365* |
| OG1 |  | *-0.547* | *-0.523* | 0.134 | *-0.687* | *-0.676* |
| CG2 |  | *-0.393* | *-0.260* | 0.134 | *-0.521* | *-0.244* |
| H |  | *0.124* | *0.146* |  | *0.122* | *0.272* |
| HA |  | *0.111* | *0.140* |  | *0.224* | *0.101* |
| HB |  | *0.045* | *0.087* |  | *0.053* | *0.004* |
| HG1 |  | *0.409* | *0.426* |  | *0.445* | *0.410* |
| HG21 |  | *0.108* | *0.077* |  | *0.129* | *0.064* |
| HG22 |  | *0.108* | *0.077* |  | *0.129* | *0.064* |
| HG23 |  | *0.108* | *0.077* |  | *0.129* | *0.064* |
|  |  |  |  |  |  |  |
| ASN 295 |  |  |  |  |  |  |
| Total charge |  | *0.034* | *0.020* | 0.076 | *0.000* | *0.000* |
| Side-chain charge |  | *0.183* | *0.185* | 0.170 | *0.239* | *0.114* |
| Backbone charge |  | *-0.150* | *-0.165* | -0.094 | *-0.239* | *-0.114* |
| N |  | *-0.129* | *-0.122* | -0.104 | *-0.615* | *-0.416* |
| CA |  | *0.023* | *-0.019* | -0.022 | *0.277* | *0.014* |
| C |  | *0.208* | *0.137* | 0.155 | *0.441* | *0.597* |
| O |  | *-0.470* | *-0.437* | -0.414 | *-0.434* | *-0.568* |
| CB |  | *-0.101* | *0.067* | 0.039 | *-0.230* | *-0.204* |
| CG |  | *0.553* | *0.345* | 0.249 | *0.542* | *0.713* |
| OD1 |  | *-0.455* | *-0.439* | -0.328 | *-0.531* | *-0.593* |
| ND2 |  | *-0.855* | *-0.580* | -0.501 | *-0.934* | *-0.919* |
| H |  | *0.241* | *0.257* | 0.269 | *0.369* | *0.272* |
| HA |  | *0.096* | *0.083* | 0.062 | *0.033* | *0.105* |
| HB2 |  | *0.059* | *0.041* | 0.037 | *0.105* | *0.080* |
| HB3 |  | *0.059* | *0.041* | 0.037 | *0.105* | *0.080* |
| HD21 |  | *0.403* | *0.324* | 0.299 | *0.437* | *0.420* |
| HD22 |  | *0.403* | *0.324* | 0.299 | *0.437* | *0.420* |
|  |  |  |  |  |  |  |
| GLU 296 |  |  |  |  |  |  |
| Total charge |  | *-0.582* | *-0.526* | -0.620 | *-1.000* | *-1.000* |
| Side-chain charge |  | *-0.669* | *-0.645* | -0.628 | *-0.888* | *-0.732* |
| Backbone charge |  | *0.087* | *0.119* | 0.009 | *-0.112* | *-0.268* |
| N |  | *-0.029* | *0.028* | -0.043 | *-0.610* | *-0.516* |
| CA |  | *-0.154* | *-0.086* | -0.052 | *-0.075* | *0.040* |
| C |  | *0.432* | *0.393* | 0.360 | *0.624* | *0.537* |
| O |  | *-0.497* | *-0.490* | -0.466 | *-0.587* | *-0.582* |
| CB |  | *-0.015* | *-0.013* | -0.056 | *0.029* | *0.056* |
| CG |  | *-0.127* | *-0.030* | -0.031 | *-0.428* | *0.014* |
| CD |  | *0.600* | *0.435* | 0.444 | *0.913* | *0.805* |
| OE1 |  | *-0.651* | *-0.592* | -0.586 | *-0.793* | *-0.819* |
| OE2 |  | *-0.651* | *-0.592* | -0.586 | *-0.793* | *-0.819* |
| H |  | *0.181* | *0.187* | 0.158 | *0.460* | *0.294* |
| HA |  | *0.149* | *0.120* | 0.117 | *0.055* | *0.111* |
| HB2 |  | *0.045* | *0.025* | 0.039 | *0.008* | *-0.017* |
| HB3 |  | *0.045* | *0.025* | 0.039 | *0.008* | *-0.017* |
| HG2 |  | *0.045* | *0.032* | 0.022 | *0.095* | *-0.043* |
| HG3 |  | *0.045* | *0.032* | 0.022 | *0.095* | *-0.043* |
|  |  |  |  |  |  |  |
| HIS/TYR 306 |  | HIS 306 | HIS 306 | TYR 306 | HIS 306 | HIS 306 |
| Total charge |  | *-0.099* | *-0.112* | -0.311 | *0.000* | *0.000* |
| Side-chain charge |  | *0.324* | *0.188* | 0.011 | *0.000* | *0.114* |
| Backbone charge |  | *-0.422* | *-0.300* | -0.322 | *0.000* | *-0.114* |
| N |  | *-0.487* | *-0.412* | -0.362 | *-0.529* | *-0.416* |
| CA |  | *0.388* | *0.116* | 0.058 | *0.016* | *-0.058* |
| C |  | *0.284* | *0.365* | 0.255 | *0.719* | *0.597* |
| O |  | *-0.505* | *-0.509* | -0.462 | *-0.550* | *-0.568* |
| CB |  | *-0.450* | *-0.082* | -0.074 | *-0.777* | *-0.007* |
| CG |  | *0.495* | *0.275* | -0.021 | *0.538* | *0.187* |
| ND1/CD1 |  | *-0.505* | *-0.471* | -0.212 | *-0.550* | *-0.543* |
| CD2 |  | *-0.387* | *-0.360* | -0.212 | *-0.393* | *-0.221* |
| CE1 |  | *0.011* | *-0.041* | -0.176 | *0.109* | *0.164* |
| NE2/CE2 |  | *-0.122* | *-0.061* | -0.176 | *-0.154* | *-0.280* |
| /CZ |  |  |  | 0.248 |  |  |
| /OH |  |  |  | -0.575 |  |  |
| H |  | *0.284* | *0.256* | 0.247 | *0.361* | *0.272* |
| HA |  | *0.052* | *0.101* | 0.120 | *0.114* | *0.136* |
| HB2 |  | *0.103* | *0.028* | 0.033 | *0.233* | *0.037* |
| HB3 |  | *0.103* | *0.028* | 0.033 | *0.233* | *0.037* |
| HD1 |  |  |  | 0.150 |  |  |
| HD2 |  | *0.167* | *0.175* | 0.132 | *0.192* | *0.186* |
| HE1 |  | *0.154* | *0.157* | 0.132 | *0.120* | *0.143* |
| HE2 |  | *0.314* | *0.321* | 0.398 | *0.321* | *0.334* |
|  |  |  |  |  |  |  |
| VAL 307 |  |  |  |  |  |  |
| Total charge |  | *0.100* | *0.051* | 0.169 | *0.000* | *0.000* |
| Side-chain charge |  | *0.170* | *0.127* | 0.118 | *-0.372* | *0.114* |
| Backbone charge |  | *-0.071* | *-0.075* | 0.051 | *0.372* | *-0.114* |
| N |  | *-0.234* | *-0.257* | -0.033 | *-0.167* | *-0.416* |
| CA |  | *-0.012* | *-0.019* | 0.006 | *-0.920* | *-0.087* |
| C |  | *0.435* | *0.397* | 0.292 | *0.772* | *0.597* |
| O |  | *-0.497* | *-0.475* | -0.415 | *-0.512* | *-0.568* |
| CB |  | *0.422* | *0.074* | 0.024 | *0.533* | *0.298* |
| CG1 |  | *-0.394* | *0.023* | -0.021 | *-0.489* | *-0.319* |
| CG2 |  | *-0.394* | *0.023* | -0.021 | *-0.489* | *-0.319* |
| H |  | *0.225* | *0.260* | 0.207 | *0.279* | *0.272* |
| HA |  | *0.092* | *0.096* | 0.085 | *0.323* | *0.097* |
| HB |  | *-0.047* | *0.023* | 0.036 | *0.013* | *-0.030* |
| HG11 |  | *0.084* | *-0.016* | 0.002 | *0.109* | *0.079* |
| HG12 |  | *0.084* | *-0.016* | 0.002 | *0.109* | *0.079* |
| HG13 |  | *0.084* | *-0.016* | 0.002 | *0.109* | *0.079* |
| HG21 |  | *0.084* | *-0.016* | 0.002 | *0.109* | *0.079* |
| HG22 |  | *0.084* | *-0.016* | 0.002 | *0.109* | *0.079* |
| HG23 |  | *0.084* | *-0.016* | 0.002 | *0.109* | *0.079* |
|  |  |  |  |  |  |  |
| ASP 310 |  |  |  |  |  |  |
| Total charge |  | *-0.901* | *-0.893* | -0.907 | *-1.000* | *-1.000* |
| Side-chain charge |  | *-0.502* | *-0.521* | -0.586 | *-0.691* | *-0.732* |
| Backbone charge |  | *-0.399* | *-0.373* | -0.321 | *-0.309* | *-0.268* |
| N |  | *-0.565* | *-0.203* | -0.102 | *-0.461* | *-0.516* |
| CA |  | *0.143* | *0.020* | 0.018 | *0.029* | *0.038* |
| C |  | *0.308* | *0.149* | 0.119 | *0.453* | *0.537* |
| O |  | *-0.475* | *-0.396* | -0.378 | *-0.509* | *-0.582* |
| CB |  | *-0.104* | *-0.023* | -0.005 | *-0.116* | *-0.030* |
| CG |  | *0.541* | *0.475* | 0.357 | *0.743* | *0.799* |
| OD1 |  | *-0.603* | *-0.524* | -0.484 | *-0.706* | *-0.801* |
| OD2 |  | *-0.603* | *-0.524* | -0.484 | *-0.706* | *-0.801* |
| H |  | *0.333* | *0.077* | 0.041 | *0.207* | *0.294* |
| HA |  | *0.052* | *0.036* | 0.000 | *0.081* | *0.088* |
| HB2 |  | *0.036* | *0.010* | 0.006 | *-0.008* | *-0.012* |
| HB3 |  | *0.036* | *0.010* | 0.006 | *-0.008* | *-0.012* |
|  |  |  |  |  |  |  |
| LEU 311 |  |  |  |  |  |  |
| Total charge |  | *0.218* | *0.231* | 0.211 | *0.000* | *0.000* |
| Side-chain charge |  | *0.015* | *0.102* | 0.127 | *0.063* | *0.114* |
| Backbone charge |  | *0.203* | *0.129* | 0.083 | *-0.063* | *-0.114* |
| N |  | *-0.067* | *-0.065* | -0.041 | *-0.491* | *-0.416* |
| CA |  | *-0.236* | *-0.105* | -0.095 | *-0.147* | *-0.052* |
| C |  | *0.271* | *0.113* | 0.057 | *0.742* | *0.597* |
| O |  | *-0.165* | *-0.085* | -0.016 | *-0.612* | *-0.568* |
| H |  | *0.164* | *0.166* | 0.084 | *0.298* | *0.272* |
| HA |  | *0.125* | *0.103* | 0.111 | *0.151* | *0.092* |
|  |  |  |  |  |  |  |
| ILE 313 |  |  |  |  |  |  |
| Total charge |  | *0.059* | *0.075* | 0.051 | *0.000* | *0.000* |
| Side-chain charge |  | *0.091* | *0.130* | 0.114 | *0.073* | *0.114* |
| Backbone charge |  | *-0.031* | *-0.055* | -0.062 | *-0.073* | *-0.114* |
| N |  | *-0.276* | *-0.284* | -0.280 | *-0.452* | *-0.416* |
| CA |  | *-0.097* | *-0.033* | -0.022 | *-0.045* | *-0.060* |
| C |  | *0.501* | *0.474* | 0.415 | *0.694* | *0.597* |
| O |  | *-0.521* | *-0.508* | -0.464 | *-0.606* | *-0.568* |
| H |  | *0.265* | *0.262* | 0.268 | *0.291* | *0.272* |
| HA |  | *0.094* | *0.082* | 0.068 | *0.013* | *0.087* |
|  |  |  |  |  |  |  |
| GLY 314 |  |  |  |  |  |  |
| Total charge |  | *0.001* | *-0.070* | 0.000 | *0.000* | *0.000* |
| Side-chain charge |  | *0.139* | *0.198* | 0.150 | *0.113* | *0.114* |
| Backbone charge |  | *-0.138* | *-0.268* | -0.150 | *-0.113* | *-0.114* |
| N |  | *-0.420* | *-0.413* | -0.339 | *-0.503* | *-0.416* |
| CA |  | *-0.128* | *-0.076* | -0.096 | *0.112* | *-0.025* |
| C |  | *0.316* | *0.119* | 0.183 | *0.587* | *0.597* |
| O |  | *-0.341* | *-0.238* | -0.239 | *-0.578* | *-0.568* |
| H |  | *0.307* | *0.264* | 0.246 | *0.380* | *0.272* |
| HA2 |  | *0.134* | *0.137* | 0.123 | *0.000* | *0.070* |
| HA3 |  | *0.134* | *0.137* | 0.123 | *0.000* | *0.070* |
|  |  |  |  |  |  |  |
| TYR 315 |  |  |  |  |  |  |
| Total charge |  | *0.204* | *0.286* | 0.192 | *0.000* | *0.000* |
| Side-chain charge |  | *0.120* | *0.170* | 0.107 | *0.035* | *0.114* |
| Backbone charge |  | *0.083* | *0.116* | 0.085 | *-0.035* | *-0.114* |
| N |  | *-0.187* | *-0.053* | -0.152 | *-0.443* | *-0.416* |
| CA |  | *-0.099* | *-0.059* | -0.151 | *-0.133* | *-0.001* |
| C |  | *0.541* | *0.437* | 0.450 | *0.615* | *0.597* |
| O |  | *-0.488* | *-0.461* | -0.439 | *-0.543* | *-0.568* |
| H |  | *0.218* | *0.193* | 0.225 | *0.336* | *0.272* |
| HA |  | *0.109* | *0.114* | 0.129 | *0.115* | *0.088* |
|  |  |  |  |  |  |  |
