# Supplementary material for: Fragment-based design of small molecule PCSK9 inhibitors using simulated annealing of chemical potential simulations
Source: PLoS One. 2019 Dec 5;14(12):e0225780. doi: 10.1371/journal.pone.0225780 (PMC6894869; doi:10.1371/journal.pone.0225780)
Supplement: S2 Table — (DOCX) [file pone.0225780.s002.docx]

**Supporting Information**

**Designing Small Molecule PCSK9 Inhibitors Guided by Simulated Annealing of Chemical Potential Simulations**

*Frank Guarnieri^1,2^, John L. Kulp Jr.^3^, John L. Kulp III^3,4^, Ian S. Cloudsdale^3^

^1^Center for Drug Discovery, Northeastern University, Boston, MA 02115 USA

^2^PAKA Pulmonary Pharmaceuticals, Acton, MA 01720 USA

^3^Conifer Point Pharmaceuticals, Doylestown, PA 18902 USA

^4^Department of Chemistry, Baruch S. Blumberg Institute, Doylestown, PA 18902 USA

*Corresponding author

Email: [frankguarnieri@yahoo.com](mailto:frankguarnieri@yahoo.com)

**Contents**

1. S1 Table. List of standard AMBER charges and custom derived charges for PCSK9-LDLR
2. S2 Table. List of fragments run on PCSK9
3. S3 Table. List of standard AMBER charges and custom charges for the CN-benzimidazole fragment bound to PCSK9
4. S1 Fig. Ball-and-stick representation of the connected path of interpenetrating atoms.
5. S2 Fig. Examples of π-π stacking.
6. S3 Fig. GAMESS input parameters
7. S4 Fig. Synthetic schemes for fragments and compounds

**S2 Table.** List of fragments run on PCSK9. 1,228 fragments were simulated on PCSK9 using SACP. Our internal naming convention was to put largest moiety first, followed by modifications off the main ring. _Prot means positively charged. _Zwit mean zwitter ionic. _minus means negatively charged. _A, _B, and tautor refers to isomers.

| 2-azabicyclo-310-hexane-1-carboxylate_Zwit | furanone-dihydro_A | propionitrile-2-NAc-2-Me_glb_B |
| --- | --- | --- |
| O=C(O)C12C(C1)CC[NH2+]2 | O=C1CCCO1 | N#CC(C)(C)NC(=O)C |
|  |  |  |
| 8-azoniabicyclo-321-octane-3-NH2-8-Me_Prot_A | furanone-dihydro_B | propionitrile-2-NAc-2-Me_med |
| C1C(N)CC([NH+]2C)CCC12 | O=C1CCCO1 | N#CC(C)(C)NC(=O)C |
|  |  |  |
| 8-azoniabicyclo-321-octane-3-NH2-8-Me_Prot_B | furo-34c-pyridine-34-dione | propionitrile-2-NAc_glb_A |
| C1C([NH3+])CC(N2C)CCC12 | O=C1OCc(c12)cc[nH]c2=O | N#CC(C)NC(=O)C |
|  |  |  |
| acetaldehyde | glutarimide | propionitrile-2-NAc_glb_B |
| O=CC | O=C1CCCC(=O)N1 | N#CC(C)NC(=O)C |
|  |  |  |
| acetaldehyde_oxime-OMe | glycine_zwiterion | propionitrile-2-NAc_high_A |
| C/C=N/OC | O=C(O)C[NH3+] | N#CC(C)NC(=O)C |
|  |  |  |
| acetamide | glycol-diOMe_med_A | propionitrile-2-NAc_high_B |
| NC(=O)C | COCCOC | N#CC(C)NC(=O)C |
|  |  |  |
| acetamide-N-3-pyridinyl-6-CF3 |  | propionitrile-2-NAc_med_A |
| CC(=O)Nc(cn1)ccc1C(F)(F)F | glycol-diOMe_med_B | N#CC(C)NC(=O)C |
|  | COCCOC |  |
| acetamide-N-5-pyrimidinyl-2-CF3 |  | propionitrile-2-NAc_med_B |
| CC(=O)Nc(cn1)cnc1C(F)(F)F | glycol_med_A | N#CC(C)NC(=O)C |
|  | OCCO |  |
| acetamide-N-diMe |  | propionitrile-2-Ph_R |
| CC(=O)N(C)C | glycol_med_B | N#CC(C)c1ccccc1 |
|  | OCCO |  |
| acetamide-N-Et_glb_A |  | propionitrile-2-Ph_S |
| CC(=O)NCC | glycolamide-N-Me-anti | N#CC(C)c1ccccc1 |
|  | OCC(=O)NC |  |
| acetamide-N-Et_glb_B |  | propyne |
| CC(=O)NCC | guanidine | CC#C |
|  | NC(=N)N |  |
| acetamide-N-Et_glb_C |  | pteridine |
| CC(=O)NCC | guanidine-1-Me-1-piperazinyl-N-Me_Prot_A | n1ccnc(c12)ncnc2 |
|  | NC(=[NH2+])N(C)N(CC1)CCN1C |  |
| acetamide-N-iPr_glb_A |  | purine |
| CC(=O)NC(C)C | guanidine-1-Me-1-piperazinyl-N-Me_Prot_B | n1c[nH]c(c12)ncnc2 |
|  | NC(=[NH2+])N(C)N(CC1)CCN1C |  |
| acetamide-N-iPr_glb_B |  | purine-2-Me |
| CC(=O)NC(C)C | guanidine-1-Me-2-CN | n1cn(C)c(c12)ncnc2 |
|  | N#C/N=C(N)\NC |  |
| acetamide-N-Me-N-Et_glb_A |  | purine-7H-8-Et-2-CF3 |
| CC(=O)N(C)CC | guanidine-1-piperazinyl-N-Me_Prot_A | c1nc(C(F)(F)F)nc(c12)nc([nH]2)CC |
|  | NC(=[NH2+])NN(CC1)CCN1C |  |
| acetamide-N-Me-N-Et_glb_B |  | pyran-2-one |
| CC(=O)N(C)CC | guanidine-1-piperazinyl-N-Me_Prot_B | O=c1cccco1 |
|  | NC(=[NH2+])NN(CC1)CCN1C |  |
| acetamide-N-Me-N-Et_high_A |  | pyran-2-one-4-OH-56-diMe |
| CC(=O)N(C)CC | guanidine-13-diMe_anti | Cc1c(C)oc(=O)cc1O |
|  | CNC(=N)NC |  |
| acetamide-N-Me-N-Et_high_B |  | pyran-4-one |
| CC(=O)N(C)CC | guanidine-13-diMe_anti_Prot | O=c1ccocc1 |
|  | CNC(=[NH2+])NC |  |
| acetamide-N-Me-N-iPr_glb |  | pyrazine |
| CC(=O)N(C)C(C)C | guanidine-13-diMe_syn | c1cnccn1 |
|  | CNC(=N)NC |  |
| acetamide-N-Me-N-iPr_med_A |  | pyrazol-3-one-2-Pr-5-Me |
| CC(=O)N(C)C(C)C | guanidine-13-diMe_syn_Prot | CCCn([nH]1)c(=O)cc1C |
|  | CNC(=[NH2+])NC |  |
| acetamide-N-Me-N-iPr_med_B |  | pyrazol-3-one-2-Pr-5-Me_B |
| CC(=O)N(C)C(C)C | guanidine-123-triMe_Prot | CCCn([nH]1)c(=O)cc1C |
|  | CNC(NC)=[NH+]C |  |
| acetamide-N-Me_Anti |  | pyrazole |
| CC(=O)NC | guanidine-1133-tetraMe_Prot_A | c1cn[nH]c1 |
|  | CN(C)C(=[NH2+])N(C)C |  |
| acetamide-N-Me_Anti_B |  | pyrazole-1H-3-CN |
| CC(=O)NC | guanidine-1133-tetraMe_Prot_B | N#Cc1cc[nH]n1 |
|  | CN(C)C(=[NH2+])N(C)C |  |
| acetamide-N-Me_Syn |  | pyrazole-1H-3-Et_A |
| CC(=O)NC | guanidine-N-2-pyridyl_A_Prot | CCc1cc[nH]n1 |
|  | NC(=[NH2+])Nc1ccccn1 |  |
| acetamide-N-OH |  | pyrazole-1H-3-Et_B |
| CC(=O)NO | guanidine-N-2-pyridyl_B_Prot | CCc1cc[nH]n1 |
|  | NC(=[NH2+])Nc1ccccn1 |  |
| acetamide-N-p-toluyl |  | pyrazole-1H-3-iPr_A |
| CC(=O)Nc(cc1)ccc1C | guanidine-N-Me | CC(C)c1cc[nH]n1 |
|  | [H]\N=C(N)\NC |  |
| acetamide-N-Ph |  | pyrazole-1H-3-iPr_B |
| CC(=O)Nc1ccccc1 | guanidine-N-Me_Prot | CC(C)c1cc[nH]n1 |
|  | NC(=[NH2+])NC |  |
| acetamidine-N-OH_Z |  | pyrazole-1H-3-Me |
| C/C(N)=N/O | guanidine-N-Ph_Prot | Cc1cc[nH]n1 |
|  | NC(=[NH2+])Nc1ccccc1 |  |
| acetamidine_E |  | pyrazole-1H-3-NHMe_glb |
| C/C(=N\[H])N | guanidine-NN'-diMe-N''-CN | CNc1cc[nH]n1 |
|  | N#CN=C(NC)NC |  |
| acetamidine_Prot |  | pyrazole-1H-3-NHMe_high |
| NC(C)=[NH2+] | guanidine-NN'-diMe-N''-NO2 | CNc1cc[nH]n1 |
|  | CNC(=N[N+]([O-])=O)NC |  |
| acetamidine_Z |  |  |
| C/C(=N/[H])N | hexafluoropropane-2-OH | pyrazole-1H-3-NMe2 |
|  | FC(F)(F)C(C(F)(F)F)O | CN(C)c1cc[nH]n1 |
| acetate |  |  |
| O=C(C)O | hexahydropyrazino-21c-14-oxazin-4-one-3-Me_Prot_A | pyrazole-1H-3-OMe_glb_A |
|  | C1OC(C)C(=O)N(C12)CC[NH2+]C2 | COc1cc[nH]n1 |
| acetic_acid |  |  |
| O=C(C)O | hexahydropyrazino-21c-14-oxazin-4-one-3-Me_Prot_B | pyrazole-1H-3-OMe_glb_B |
|  | C1OC(C)C(=O)N(C12)CC[NH2+]C2 | COc1cc[nH]n1 |
|  |  |  |
| acetic_acid_A | hydantoin | pyrazole-1H-3-OMe_high |
| O=C(C)O | O=C1CNC(=O)N1 | COc1cc[nH]n1 |
|  |  |  |
| acetone | hydantoin-1-Me | pyrazole-1H-4-OH_A |
| CC(=O)C | N1C(=O)N(C)CC1=O | Oc1cn[nH]c1 |
|  |  |  |
| acetone_oxime | hydantoin-3-Et_A | pyrazole-1H-4-OH_B |
| CC(C)=NO | CCN1C(=O)NCC1=O | Oc1cn[nH]c1 |
|  |  |  |
| acetone_oxime-O-Me | hydantoin-3-Et_B | pyrazole-2H-3-CN |
| CC(C)=NOC | CCN1C(=O)NCC1=O | N#Cc1ccn[nH]1 |
|  |  |  |
| acetonitrile | hydantoin-3-Me | pyrazole-2H-3-NHMe_glb |
| N#CC | CN1C(=O)NCC1=O | CNc1ccn[nH]1 |
|  |  |  |
| acetonitrile-2-Me | hydantoin-13-diMe | pyrazole-2H-3-NHMe_med |
| N#CC(C)C | CN1C(=O)N(C)CC1=O | CNc1ccn[nH]1 |
|  |  |  |
| acetonitrile-N-Me_glb_A |  | pyrazole-2H-3-NMe2 |
| N#CCNC | i-butane | CN(C)c1ccn[nH]1 |
|  | CC(C)C |  |
| acetonitrile-N-Me_glb_B |  | pyrazole-2H-3-OMe_glb_A |
| N#CCNC | i-propylamine | COc1ccn[nH]1 |
|  | CC(C)N |  |
| acetonitrile-N-Me_med_A |  | pyrazole-2H-3-OMe_glb_B |
| N#CCNC | imidazo-12a-pyrimidine-57-diNH2 | COc1ccn[nH]1 |
|  | n1ccn(c12)c(N)cc(n2)N |  |
| acetonitrile-N-Me_med_B |  | pyrazole-2H-3-OMe_med |
| N#CCNC | imidazo-12a-tetrahydropyrimidine_Prot_A | COc1ccn[nH]1 |
|  | N1C=CN(C12)CCCN2 |  |
| acetonitrile-NAc_glb |  | pyrazole-4-Me |
| N#CCNC(=O)C | imidazo-15a-pyrazin-3-one-octahydro-2-Me-Prot_S | Cc1cn[nH]c1 |
|  | CN(C1)C(=O)N(C12)CC[NH2+]C2 |  |
| acetonitrile-NAc_med_A |  | pyrazole-14-diMe-3-OH |
| N#CCNC(=O)C | imidazo-15a-tetrahydropyrazine-3-NH2-7-Me_Prot_A | Cc1c(O)nn(C)c1 |
|  | CN(C1)CCN(C1=2)C(N)NC2 |  |
| acetonitrile-NAc_med_B |  | pyrazole-135-triMe |
| N#CCNC(=O)C | imidazo-45c-pyridine-1H | Cc1cc(C)n(n1)C |
|  | n1c[nH]c(c12)ccnc2 |  |
| acetophenone |  | pyrazole-N-Me |
| CC(=O)c1ccccc1 | imidazo-45c-pyridine-2-Et-1H_B | Cn1cccn1 |
|  | CCc(n1)[nH]c(c12)ccnc2 |  |
| acetophenone-2-OH |  | pyrazolone-1-Me |
| CC(=O)c1c(O)cccc1 | imidazo-45c-pyridine-2-Et-3H_A | Cc1cc(=O)[nH][nH]1 |
|  | CCc(n1)[nH]c(c12)cncc2 |  |
| acetophenone-3-OH |  | pyrazolone-15-diMe |
| CC(=O)c1cc(O)ccc1 | imidazo-45c-pyridine-3H | Cc1cc(=O)[nH]n1C |
|  | n1c[nH]c(c12)cncc2 |  |
| acrylonitrile-3NH2-3-piperazine-4Me_A |  | pyridazine |
| N#C/C=C(\N)N1CCN(C)CC1 | imidazo-45c-pyridine-3H-2-Et-6-CF3_A | c1ccnnc1 |
|  | FC(F)(F)c(c1)ncc(c12)[nH]c(n2)CC |  |
| acrylonitrile-3NH2-3-piperazine-4Me_B |  | pyridazine-3-Ac_B |
| N#C/C=C(\N)N1CCN(C)CC1 | imidazo-51c-dihydrooxazine-3-NH2_Prot_A | CC(=O)c1cccnn1 |
|  | C1NC(N)N(C=12)CCOC2 |  |
| adamantane |  | pyridazine-4-NH2 |
| C12CC3CC(C1)CC(C2)C3 | imidazo-d-cyclopentane-2-NH2_Prot | Nc1ccnnc1 |
|  | NC(N1)NC(=C12)CCC2 |  |
| adamantane-1-amidinium |  | pyridin-2-one |
| NC(=[NH2+])C12CC3CC(C1)CC(C2)C3 | imidazole | O=c1cccc[nH]1 |
|  | c1c[nH]cn1 |  |
| adamantane-1-Me |  | pyridin-2-one-36-diMe |
| CC12CC3CC(C1)CC(C2)C3 | imidazole-1H-4-Me | Cc1c(=O)[nH]c(C)cc1 |
|  | Cc1c[nH]cn1 |  |
| adamantane-1-NH2-35-diF_Prot |  | pyridin-2-one-56-dihydro_A |
| [NH3+]C12CC3(F)CC(F)(C1)CC(C2)C3 | imidazole-2-CH2CN_A | O=C1C=CCCN1 |
|  | N#CCc1ncc[nH]1 |  |
| adamantane-1-NH2-44-diF_Prot |  | pyridin-2-one-56-dihydro_B |
| [NH3+]C12CC3C(F)(F)C(C1)CC(C2)C3 | imidazole-2-CH2OH_glb_A | O=C1C=CCCN1 |
|  | OCc1ncc[nH]1 |  |
| adamantane-1-NH2_Prot |  | pyridin-2-one-346-triMe |
| [NH3+]C12CC3CC(C1)CC(C2)C3 | imidazole-2-CH2OH_glb_B | Cc1c(C)cc(C)[nH]c1=O |
|  | OCc1ncc[nH]1 |  |
| adamantane-2-NH2_Prot |  | pyridin-2-one-N-Me |
| C12C([NH3+])C3CC(C1)CC(C2)C3 | imidazole-2-CH2OH_med_A | Cn1ccccc1=O |
|  | OCc1ncc[nH]1 |  |
| adenine |  | pyridin-2-one-N-Me-3-CN |
| n1c[nH]c(c12)ncnc2N | imidazole-2-CH2OH_med_B | N#Cc1c(=O)n(C)ccc1 |
|  | OCc1ncc[nH]1 |  |
| adenine-N-Me |  | pyridin-2-one-N-Me-3-CN-6-OH-4-Me |
| n1cn(C)c(c12)ncnc2N | imidazole-2-CH2OMe_A | N#Cc1c(C)cc(O)n(C)c1=O |
|  | COCc1ncc[nH]1 |  |
| aniline |  | pyridin-2-one-N-Me-5-Me |
| Nc1ccccc1 | imidazole-2-CH2OMe_B | Cc1ccc(=O)n(C)c1 |
|  | COCc1ncc[nH]1 |  |
| aniline-2-Me |  | pyridin-3-Me-4-one |
| Cc1c(N)cccc1 | imidazole-2-CHMeCN_glb_A | CC1=CNC=CC1O |
|  | N#CC(C)c1ncc[nH]1 |  |
| aniline-3-Cl |  | pyridin-4-one |
| Nc1cc(Cl)ccc1 | imidazole-2-CHMeCN_glb_B | OC1=CCNC=C1 |
|  | N#CC(C)c1ncc[nH]1 |  |
| aniline-3-SMe |  |  |
| Nc1cc(SC)ccc1 | imidazole-2-CHMeCN_glb_C | pyridin-4-one-2-NH2-6-Me |
|  | N#CC(C)c1ncc[nH]1 | CC1=CC(O)C=C(N)N1 |
| aniline-N-diMe |  |  |
| CN(C)c1ccccc1 | imidazole-2-CHMeCN_glb_D | pyridine |
|  | N#CC(C)c1ncc[nH]1 | c1ccncc1 |
| aniline-N-Me |  |  |
| CNc1ccccc1 | imidazole-2-CHMeOH_glb_A | pyridine-2-CF3 |
|  | CC(O)c1ncc[nH]1 | FC(F)(F)c1ccccn1 |
|  |  |  |
| aniline-N-Me-3-CF3_pcsk9 | imidazole-2-CHMeOH_glb_B | pyridine-2-Cl |
| FC(F)(F)c1cc(NC)ccc1 | CC(O)c1ncc[nH]1 | Clc1ccccn1 |
|  |  |  |
| aniline-N-Me_A | imidazole-2-CHMeOMe_glb_A | pyridine-2-CN |
| CNc1ccccc1 | COC(C)c1ncc[nH]1 | N#Cc1ccccn1 |
|  |  |  |
| anisidine-m-syn | imidazole-2-CHMeOMe_glb_B | pyridine-2-CN-3-Cl-4-Me |
| Nc1cc(OC)ccc1 | COC(C)c1ncc[nH]1 | N#Cc1c(Cl)c(C)ccn1 |
|  |  |  |
| anisole | imidazole-2-CHMeOMe_glb_C | pyridine-2-CN-3-Cl-45-diMe |
| COc1ccccc1 | COC(C)c1ncc[nH]1 | N#Cc1c(Cl)c(C)c(C)cn1 |
|  |  |  |
| anisole-2-Cl | imidazole-2-CHMeOMe_glb_D | pyridine-2-Et_A |
| COc1c(Cl)cccc1 | COC(C)c1ncc[nH]1 | CCc1ccccn1 |
|  |  |  |
| anisole-4-Me | imidazole-2-CMe2CN_glb | pyridine-2-Et_B |
| Cc1ccc(cc1)OC | N#CC(C)(C)c1ncc[nH]1 | CCc1ccccn1 |
|  |  |  |
| anisole_48deg_A |  | pyridine-2-iPr_A |
| COc1ccccc1 | imidazole-2-CMe2CN_med_A | CC(C)c1ccccn1 |
|  | N#CC(C)(C)c1ncc[nH]1 |  |
| azepane |  | pyridine-2-iPr_B |
| C1CCCNCC1 | imidazole-2-CMe2CN_med_B | CC(C)c1ccccn1 |
|  | N#CC(C)(C)c1ncc[nH]1 |  |
| azepin-2-one-N-Me_A |  | pyridine-2-Me |
| CN1CC=CCCC1=O | imidazole-2-CMe2OH_glb | Cc1ccccn1 |
|  | CC(C)(O)c1ncc[nH]1 |  |
| azepin-2-one-N-Me_B |  | pyridine-2-NH2 |
| CN1CC=CCCC1=O | imidazole-2-CMe2OH_med_A | Nc1ccccn1 |
|  | CC(C)(O)c1ncc[nH]1 |  |
| azetidine |  | pyridine-2-NH2-3-CN |
| C1CNC1 | imidazole-2-CMe2OH_med_B | N#Cc1c(N)nccc1 |
|  | CC(C)(O)c1ncc[nH]1 |  |
| azetidine-1-Me |  | pyridine-2-NH2-4-Et-6-OMe_44_rot_Prot |
| CN1CCC1 | imidazole-2-CMe2OMe_glb | C1=C(N)NC(CC)C=C1OC |
|  | COC(C)(C)c1ncc[nH]1 |  |
| b-carboline |  | pyridine-2-NH2-4-Et-6-OMe_Prot |
| c1cncc(c1c23)[nH]c2cccc3 | imidazole-2-CMe2OMe_med_A | CCC1=CC(OC)C=C(N)N1 |
|  | COC(C)(C)c1ncc[nH]1 |  |
| benzaldehyde-2-NHMe |  | pyridine-2-NH2-4-Me-6-Et_Prot_A |
| O=Cc1c(NC)cccc1 | imidazole-2-CMe2OMe_med_B | CCC1=CC(C)C=C(N)N1 |
|  | COC(C)(C)c1ncc[nH]1 |  |
| benzamide |  | pyridine-2-NH2-4-NHMe-6-OMe_Prot |
| NC(=O)c1ccccc1 | imidazole-2-CN | CNC1=CC(OC)NC(=C1)N |
|  | N#Cc1ncc[nH]1 |  |
| benzamide-3-NH2-5-CN |  | pyridine-2-NH2-4-OMe-6-Et_90_rot_Prot |
| N#Cc1c(C(=O)N)cc(N)cc1 | imidazole-2-CN-4-Me | CCC1=CC(OC)=CC(N)N1 |
|  | N#Cc1nc(C)c[nH]1 |  |
| benzamide-3-NH2_syn |  | pyridine-2-NH2-4-OMe-6-Et_Prot |
| NC(=O)c1cc(N)ccc1 | imidazole-2-CN-5-Me | CCC1=CC(OC)=CC(N)N1 |
|  | N#Cc1ncc(C)[nH]1 |  |
| benzamide-3-NHAc-5-CN |  | pyridine-2-NH2-4-OMe-6-Me_90_rot_Prot |
| N#Cc1c(C(=O)N)cc(cc1)NC(=O)C | imidazole-2-Et_A | C1=C(N)NC(C)C=C1OC |
|  | CCc1ncc[nH]1 |  |
| benzamide-3-NHMe-5-CN |  | pyridine-2-NH2-4-OMe-6-Me_Prot |
| N#Cc1c(C(=O)N)cc(cc1)NC | imidazole-2-Et_B | C1=C(N)NC(C)C=C1OC |
|  | CCc1ncc[nH]1 |  |
| benzamide-N-Me |  | pyridine-2-NH2-4-OMe-6-Me_Prot_B |
| CNC(=O)c1ccccc1 | imidazole-2-iPr_A | CC1=CC(OC)=CC(N)N1 |
|  | CC(C)c1ncc[nH]1 |  |
| benzamide-N-Me-3-amidino_20deg_Prot |  | pyridine-2-NH2-4-OMe_90_rot_Prot |
| CNC(=O)c1cc(C(N)N)ccc1 | imidazole-2-iPr_B | N1C=CC(OC)C=C1N |
|  | CC(C)c1ncc[nH]1 |  |
| benzamide-N-Me-3-amidino_Prot |  | pyridine-2-NH2-4-OMe_Prot_A |
| CNC(=O)c1cc(C(N)N)ccc1 | imidazole-2-Me | COC1=CCNC(=C1)N |
|  | Cc1ncc[nH]1 |  |
| benzamide-N-Ph |  | pyridine-2-NH2-4-OMe_Prot_B |
| c1ccccc1C(=O)Nc2ccccc2 | imidazole-2-Me_Prot | COC1=CCNC(=C1)N |
|  | CC1NC=CN1 |  |
| benzamidine |  | pyridine-2-NH2-6-CN |
| N=C(N)c1ccccc1 | imidazole-2-NH2 | N#Cc1cccc(n1)N |
|  | Nc1ncc[nH]1 |  |
| benzamidine_Prot |  | pyridine-2-NH2-6-Et-4Me_Prot |
| NC(N)c1ccccc1 | imidazole-2-NH2-4-Ac_Prot | CCC1=CC(C)=CC(N)N1 |
|  | CC(=O)C1=CNC(N)N1 |  |
| benzene |  | pyridine-2-NH2-6-Et_Prot |
| c1ccccc1 | imidazole-2-NH2-4-Me_Prot | CCC1=CCC=C(N)N1 |
|  | CC1=CNC(N)N1 |  |
| benzene-1-CF3-4-Me |  | pyridine-2-NH2-46-diMe_Prot |
| FC(F)(F)c1ccc(C)cc1 | imidazole-2-NH2-45-diMe_Prot | CC1=CC(C)NC(=C1)N |
|  | CC1=C(C)NC(N)N1 |  |
| benzene-1-Cl-2-F |  | pyridine-2-NH2-46-diMeO_Prot_A |
| Fc1c(Cl)cccc1 | imidazole-2-NHMe_glb | COC(N1)=CC(OC)C=C1N |
|  | CNc1ncc[nH]1 |  |
| benzene-1-Cl-2-Me |  | pyridine-2-NHMe_glb |
| Cc1c(Cl)cccc1 | imidazole-2-NHMe_med | CNc1ccccn1 |
|  | CNc1ncc[nH]1 |  |
| benzene-1-Et-4-Me |  | pyridine-2-NHMe_high |
| CCc1ccc(C)cc1 | imidazole-2-NMe2 | CNc1ccccn1 |
|  | CN(C)c1ncc[nH]1 |  |
| benzene-1-OMe |  |  |
| COc1ccccc1 | imidazole-2-OMe_high | pyridine-2-NMe2 |
|  | COc1ncc[nH]1 | CN(C)c1ccccn1 |
| benzene-12-diCl |  |  |
| Clc1c(Cl)cccc1 | imidazole-2-SMe | pyridine-2-OMe |
|  | CSc1ncc[nH]1 | COc1ccccn1 |
| benzene-13-diCl |  |  |
| Clc1cc(Cl)ccc1 | imidazole-4-Me_Prot | pyridine-3-CF3 |
|  | CC1=CNCN1 | FC(F)(F)c1cccnc1 |
|  |  |  |
| benzene-124-triOH_A | imidazole-4-SMe | pyridine-3-Cl |
| Oc1c(O)ccc(c1)O | CSc1c[nH]cn1 | Clc1cccnc1 |
|  |  |  |
| benzene-124-triOH_B | imidazole-5-SMe | pyridine-3-CN |
| Oc1c(O)ccc(c1)O | CSc1cnc[nH]1 | N#Cc1cccnc1 |
|  |  |  |
| benzene-CF3 | imidazole-45-dihydro | pyridine-3-CONH2-1-Me-14-dihydro |
| FC(F)(F)c1ccccc1 | C1=NCCN1 | NC(=O)C1=CN(C)C=CC1 |
|  |  |  |
| benzene-Cl | imidazole-45-diMe | pyridine-3-Et_A |
| Clc1ccccc1 | Cc1c(C)[nH]cn1 | CCc1cccnc1 |
|  |  |  |
| benzene-Et | imidazole-N-Me | pyridine-3-Et_B |
| CCc1ccccc1 | Cn1ccnc1 | CCc1cccnc1 |
|  |  |  |
| benzene-F | imidazole-N-Me-2-NH2_Prot | pyridine-3-iPr_A |
| Fc1ccccc1 | NC1N(C)C=CN1 | CC(C)c1cccnc1 |
|  |  |  |
| benzene-iPr |  | pyridine-3-iPr_B |
| CC(C)c1ccccc1 | imidazole-N-Me_Prot | CC(C)c1cccnc1 |
|  | CN1C=CNC1 |  |
| benzene-NHAc_cis |  | pyridine-3-Me |
| CC(=O)Nc1ccccc1 | imidazole_Prot | Cc1cccnc1 |
|  | C1=CNCN1 |  |
| benzene-NHAc_trans |  | pyridine-3-NH2 |
| CC(=O)Nc1ccccc1 | imidazolidin-2-one-N-Me-N-Et_A | Nc1cccnc1 |
|  | CCN(C1=O)CCN1C |  |
| benzene-O-cyclopentyl_A |  | pyridine-3-NHAc |
| C1CCCC1Oc2ccccc2 | imidazolidin-2-one-N-Me-N-iPr | CC(=O)Nc1cccnc1 |
|  | CC(C)N(C1=O)CCN1C |  |
| benzene-OCF3 |  | pyridine-3-NHMe_glb |
| FC(F)(F)Oc1ccccc1 | imidazolidin-2-one-NN-diMe | CNc1cccnc1 |
|  | O=C1N(C)CCN1C |  |
| benzene-OEt |  | pyridine-3-NHMe_high |
| CCOc1ccccc1 | imidazolidine-2-NH-1-Me_Prot | CNc1cccnc1 |
|  | [NH2+]=C1N(C)CCN1 |  |
| benzimidazole |  | pyridine-3-NMe2 |
| n1c[nH]c(c12)cccc2 | imidazolidine-2-NH-13-diMe_Prot | CN(C)c1cccnc1 |
|  | [NH2+]=C1N(C)CCN1C |  |
| benzimidazole-2-CF3 |  | pyridine-3-OH_A |
| FC(F)(F)c(n1)[nH]c(c12)cccc2 | imidazolidinone-N-Me | Oc1cccnc1 |
|  | O=C1N(C)CCN1 |  |
| benzimidazole-2-CF3-5-Et_A |  | pyridine-3-OH_B |
| FC(F)(F)c(n1)[nH]c(c12)ccc(c2)CC | indane | Oc1cccnc1 |
|  | C1CCc(c12)cccc2 |  |
| benzimidazole-2-CN |  | pyridine-3-OMe_A |
| N#Cc(n1)[nH]c(c12)cccc2 | indane-2-NH2-2-Me_Prot_A | COc1cccnc1 |
|  | CC(C1)([NH3+])Cc(c12)cccc2 |  |
| benzimidazole-2-CN-7-Me |  | pyridine-3-OMe_B |
| N#Cc([nH]1)nc(c12)cccc2C | indane-2-NH2-2-Me_Prot_B | COc1cccnc1 |
|  | CC(C1)([NH3+])Cc(c12)cccc2 |  |
| benzimidazole-2-CO2_anion |  | pyridine-3-Ph_B |
| O=C(O)c(n1)[nH]c(c12)cccc2 | indane-2-NH2_Prot | c1ccccc1-c2cccnc2 |
|  | C1C([NH3+])Cc(c12)cccc2 |  |
| benzimidazole-2-Et-4-Ac |  | pyridine-4-Cl |
| CC(=O)c1cccc(c12)[nH]c(n2)CC | indane-2-NHMe_Prot_A | Clc1ccncc1 |
|  | C[NH2+]C(C1)Cc(c12)cccc2 |  |
| benzimidazole-2-Et-4-CF3 |  | pyridine-4-Me |
| FC(F)(F)c1cccc(c12)[nH]c(n2)CC | indane-2-NHMe_Prot_B | Cc1ccncc1 |
|  | C[NH2+]C(C1)Cc(c12)cccc2 |  |
| benzimidazole-2-Et-4-CF3-5-iPr |  | pyridine-4-NH2 |
| FC(F)(F)c1c(C(C)C)ccc(c12)[nH]c(n2)CC | indane-2-OH-1-CN-1R2S_A | Nc1ccncc1 |
|  | N#CC1C(O)Cc(c12)cccc2 |  |
| benzimidazole-2-Et-4-CN |  | pyridine-4-NH2_Prot |
| N#Cc1cccc(c12)[nH]c(n2)CC | indazole | NC1=CCNC=C1 |
|  | c1n[nH]c(c12)cccc2 |  |
| benzimidazole-2-Et-4-CN-5-Ac |  | pyridine-12a-imidazo |
| N#Cc1c(C(=O)C)ccc(c12)[nH]c(n2)CC | indazole-3-CF3 | n1ccn(c12)cccc2 |
|  | FC(F)(F)c1n[nH]c(c12)cccc2 |  |
| benzimidazole-2-Et-4-CN-5-CH2OSO2NH2_A |  | pyridine-24-diMe |
| NS(=O)(=O)OCc(c1C#N)ccc(c12)[nH]c(n2)CC | indazole-3-NH2 | Cc1cc(C)ncc1 |
|  | Nc1n[nH]c(c12)cccc2 |  |
| benzimidazole-2-Et-4-CN-5-CH2OSO2NH2_B |  | pyridine-24-diNH2-6-OMe_Prot |
| NS(=O)(=O)OCc(c1C#N)ccc(c12)[nH]c(n2)CC | indazole-3-NH2_Prot | NC1=CC(OC)NC(=C1)N |
|  | NC1NNc(c12)cccc2 |  |
| benzimidazole-2-Et-4-CN-5-Et_Ar |  | pyridine_Prot |
| CCOc(c1C#N)ccc(c12)[nH]c(n2)CC | indazole-3-OH | C1=CCNC=C1 |
|  | Oc1n[nH]c(c12)cccc2 |  |
| benzimidazole-2-Et-4-CN-5-OMe |  | pyridinone-4-OH-N-Me |
| N#Cc1c(OC)ccc(c12)[nH]c(n2)CC | indazole-7-OH | c1cn(C)c(=O)cc1O |
|  | c1[nH]nc(c12)c(O)ccc2 |  |
| benzimidazole-2-Et-4-CN-5-OMe_Ar |  | pyrido-12a-pyrimidin-3-ol-dihydro-8-Me_Prot |
| N#Cc1c(OC)ccc(c12)[nH]c(n2)CC | indazole-N-Me | CC(C1)C=CN(C=12)CC(O)CN2 |
|  | c1nn(C)c(c12)cccc2 |  |
| benzimidazole-2-Et-4-CN-5-OMe_Ar_B |  | pyrido-23b-pyrazine-8-one-34-dihydro-6-NH2 |
| N#Cc1c(OC)ccc(c12)[nH]c(n2)CC | indene | N1=CCNC(=C12)NC(N)C=C2O |
|  | C1=CCc(c12)cccc2 |  |
| benzimidazole-2-Et-4-CN-5-OSO2Me_A |  | pyrido-23b-pyrazine-8-one-34-dihydro-6-NH2-2-Me |
| CS(=O)(=O)Oc(c1C#N)ccc(c12)[nH]c(n2)CC | indole | CC(=N1)CNC(C1=2)NC(N)=CC2O |
|  | c1c[nH]c(c12)cccc2 |  |
| benzimidazole-2-Et-4-CN-5-OSO2Me_B |  |  |
| CS(=O)(=O)Oc(c1C#N)ccc(c12)[nH]c(n2)CC | indole-2-CN | pyrido-23d-pyrimidin-2-one |
|  | N#Cc(c1)[nH]c(c12)cccc2 | c1nc(=O)[nH]c(c12)nccc2 |
| benzimidazole-2-Et-4-CN-5-OSO2NH2 |  |  |
| N#Cc1c(OS(=O)(=O)N)ccc(c12)[nH]c(n2)CC | indole-2-CN-7-Me | pyrido-34b-pyrazine-34-dihydro-2-one-1-Me |
|  | N#Cc(c1)[nH]c(c12)c(C)ccc2 | N1CC(=O)N(C)C(C1=2)C=CNC2 |
| benzimidazole-2-Et-5-CF3_A |  |  |
| FC(F)(F)c(c1)ccc(c12)[nH]c(n2)CC | indole-2-Et_A | pyrido-34c-125-thiadiazine-34-dihydro-22-dioxide_Prot |
|  | CCc(c1)[nH]c(c12)cccc2 | N1S(=O)(=O)CNC(C1=2)C=CNC2 |
|  |  |  |
| benzimidazole-2-Et-5-Cl_A | indole-2-Et_B | pyrido-34d-pyrimidin-4-one-tetrahydro-2-NH2-17-diMe_A |
| CCc(n1)[nH]c(c12)ccc(Cl)c2 | CCc(c1)[nH]c(c12)cccc2 | OC1=NC(N)N(C)C(=C12)CN(C)CC2 |
|  |  |  |
| benzimidazole-2-Et-6-Cl_A | indole-2-Me | pyrido-34d-pyrimidin-4-one-tetrahydro-2-NH2-17-diMe_B |
| CCc(n1)[nH]c(c12)cc(Cl)cc2 | Cc(c1)[nH]c(c12)cccc2 | OC1=NC(N)N(C)C(=C12)CN(C)CC2 |
|  |  |  |
| benzimidazole-2-Et-56-diCl_A | indole-2-NHMe-3-CN | pyrido-43b-indole-4-NH2 |
| CCc(n1)[nH]c(c12)cc(Cl)c(Cl)c2 | N#Cc1c(NC)[nH]c(c12)cccc2 | c1cccc(c1c23)[nH]c2c(N)cnc3 |
|  |  |  |
| benzimidazole-2-Et_A | indole-2-NHMe-3-CN-5-OH | pyrido-43c-125-thiadiazine-34-dihydro-22-dioxide_Prot |
| CCc(n1)[nH]c(c12)cccc2 | N#Cc1c(NC)[nH]c(c12)ccc(c2)O | N1S(=O)(=O)CNC(C1=2)=CNCC2 |
|  |  |  |
| benzimidazole-2-Et_B | indole-2-NHMe-3-NO2 | pyrido-43d-pyrimidin-4-one-1-Me |
| CCc(n1)[nH]c(c12)cccc2 | [O-][N+](=O)c1c(NC)[nH]c(c12)cccc2 | OC1N=CN(C)c(c12)ccnc2 |
|  |  |  |
| benzimidazole-2-Me | indole-2-NHMe-3-NO2-5-OH | pyrido-43d-pyrimidin-4-one-2-NH2-6-Me--5678-tetrahydro_A |
| Cc(n1)[nH]c(c12)cccc2 | [O-][N+](=O)c1c(NC)[nH]c(c12)ccc(c2)O | CN(C1)CCC(C1=2)NC(N)=NC2O |
|  |  |  |
| benzimidazole-2-Me-4-CN |  | pyrido-43d-pyrimidin-4-one-2-NH2-6-Me--5678-tetrahydro_B |
| N#Cc1cccc(c12)[nH]c(n2)C | indole-3-amino | CN(C1)CCC(=C12)NC(N)=NC2O |
|  | Nc1c[nH]c(c12)cccc2 |  |
| benzimidazole-2-Me-5-Et_A |  | pyridone-1-Me |
| CCc(c1)ccc(c12)[nH]c(n2)C | indole-3-CF3-6-NHEt | Cn1ccccc1=O |
|  | FC(F)(F)c1c[nH]c(c12)cc(cc2)NCC |  |
| benzimidazole-2-NH2 |  | pyridone-4-Me |
| Nc(n1)[nH]c(c12)cccc2 | indole-3-CN | Cc1cc(=O)[nH]cc1 |
|  | N#Cc1c[nH]c(c12)cccc2 |  |
| benzimidazole-2-NH2-4-CN |  | pyridone-14-diMe |
| N#Cc1cccc(c12)[nH]c(n2)N | indole-3-CN-2-Me | Cc1cc(=O)n(C)cc1 |
|  | N#Cc1c(C)[nH]c(c12)cccc2 |  |
| benzimidazole-2-NH2-4-CN-5-1-MeOEt-7Me |  | pyrimid-2-one-4-NH2 |
| COC(C)c(c1C#N)cc(C)c(c12)[nH]c(n2)N | indole-3-CN-2-Me-7-NH2 | Nc1cc[nH]c(n1)=O |
|  | N#Cc1c(C)[nH]c(c12)c(N)ccc2 |  |
| benzimidazole-2-NH2-4-CN-5-Ac-7Me |  | pyrimid-2-one-456-triMe |
| N#Cc1c(C(=O)C)cc(C)c(c12)[nH]c(n2)N | indole-3-CN-2-Me-7-NHMe | Cc1c(C)nc(=O)[nH]c1C |
|  | N#Cc1c(C)[nH]c(c12)c(NC)ccc2 |  |
| benzimidazole-2-NH2-4-CN-5-CF3-7-Me |  | pyrimidin-1-one-46-diMe |
| N#Cc1c(C(F)(F)F)cc(C)c(c12)[nH]c(n2)N | indole-3-CN-7-Me | Cc1cc(C)[nH]c(n1)=O |
|  | N#Cc1c[nH]c(c12)c(C)ccc2 |  |
| benzimidazole-2-NH2-4-CN-5-iPr |  | pyrimidin-2-one |
| N#Cc1c(C(C)C)ccc(c12)[nH]c(n2)N | indole-3-Me | O=c1nccc[nH]1 |
|  | Cc1c[nH]c(c12)cccc2 |  |
| benzimidazole-2-NH2-4-CN-5-iPr-7-Me |  | pyrimidin-2-one-46-diMe |
| N#Cc1c(C(C)C)cc(C)c(c12)[nH]c(n2)N | indole-5-CN | Cc1cc(C)[nH]c(n1)=O |
|  | N#Cc(c1)ccc(c12)[nH]cc2 |  |
| benzimidazole-2-NH2-4-CN-5-MeOMe-7Me |  | pyrimidin-2-thione-4-NH2 |
| COCc(c1C#N)cc(C)c(c12)[nH]c(n2)N | indole-5-CN-2-Et_A | Nc1cc[nH]c(n1)=S |
|  | N#Cc(c1)ccc(c12)[nH]c(c2)CC |  |
| benzimidazole-2-NH2-4-CN-5-OMe |  | pyrimidin-2-thione-4-NH2-1-Me |
| N#Cc1c(OC)ccc(c12)[nH]c(n2)N | indole-5-CN-2-Et_B | n1c(=S)n(C)ccc1N |
|  | N#Cc(c1)ccc(c12)[nH]c(c2)CC |  |
| benzimidazole-2-NH2-4-CN-5-OMe-7-CCEt |  | pyrimidin-4-one-1-Me |
| CCC#Cc(c(c12)[nH]c(n2)N)cc(c1C#N)OC | indole-5-CN-6-CH2OMe | N1=CN(C)C=CC1O |
|  | COCc(c(C#N)c1)cc(c12)[nH]cc2 |  |
| benzimidazole-2-NH2-4-CN-5-OMe-7-Me |  | pyrimidin-4-one-2-NH2-1-Me |
| N#Cc1c(OC)cc(C)c(c12)[nH]c(n2)N | indole-5-CN-6-Et_A | CN1C=CC(O)N=C1N |
|  | N#Cc(c1)c(CC)cc(c12)[nH]cc2 |  |
| benzimidazole-2-NH2-4-CN-5-OMe-7-Me_90deg |  | pyrimidin-4-one-2-NH2-6-Me-1H |
| N#Cc1c(OC)cc(C)c(c12)[nH]c(n2)N | indole-5-CN-6-OMe | CC1=CC(O)N=C(N)N1 |
|  | N#Cc(c1)c(OC)cc(c12)[nH]cc2 |  |
| benzimidazole-2-NH2-4-CN-5-OMe-7-propyne |  | pyrimidin-4-one-15-diMe |
| Nc(n1)[nH]c(c12)c(C#CC)cc(c2C#N)OC | indole-5-CN-6-OMe-7-Me_A | CC1=CN(C)CN=C1O |
|  | N#Cc(c1)c(OC)c(C)c(c12)[nH]cc2 |  |
| benzimidazole-2-NH2-4-CN-5-OMe_90deg |  | pyrimidindione-6-Me |
| N#Cc1c(OC)ccc(c12)[nH]c(n2)N | indole-12-diMe | Cc1cc(=O)[nH]c(=O)[nH]1 |
|  | Cc(c1)n(C)c(c12)cccc2 |  |
| benzimidazole-2-NH2-4-CN-5-OMe_90deg_tautomer |  | pyrimidine |
| N#Cc1c(OC)ccc(c12)nc(N)[nH]2 | indole-35-diCN-6-Me | c1cncnc1 |
|  | N#Cc1c[nH]c(c12)cc(C)c(C#N)c2 |  |
| benzimidazole-2-NH2-4-Me_Prot |  | pyrimidine-2-CF3 |
| Cc1cccc(c12)NC(N)N2 | indole-N-Me | FC(F)(F)c1ncccn1 |
|  | c1cn(C)c(c12)cccc2 |  |
| benzimidazole-2-NH2-5-Cl |  | pyrimidine-2-CN |
| Nc(n1)[nH]c(c12)ccc(Cl)c2 | indolin-2-one-5-CN | N#Cc1ncccn1 |
|  | N#Cc(c1)ccc(c12)NC(=O)C2 |  |
| benzimidazole-2-NH2-6-OH-7-Me_Prot |  | pyrimidine-2-Et_A |
| Cc1c(O)ccc(c12)NC(N)N2 | indolin-2-one-5-Et_A | CCc1ncccn1 |
|  | CCc(c1)ccc(c12)NC(=O)C2 |  |
| benzimidazole-2-NH2-45-diCl |  | pyrimidine-2-Et_B |
| Nc(n1)[nH]c(c12)ccc(Cl)c2Cl | indolin-2-one-35-diCN_A | CCc1ncccn1 |
|  | N#CC1C(=O)Nc(c12)ccc(C#N)c2 |  |
| benzimidazole-2-NH2_Prot |  | pyrimidine-2-iPr |
| NC(N1)Nc(c12)cccc2 | indoline | CC(C)c1ncccn1 |
|  | C1CNc(c12)cccc2 |  |
| benzimidazole-4-CN |  | pyrimidine-2-Me |
| N#Cc1cccc(c12)[nH]cn2 | indolinone | Cc1ncccn1 |
|  | C1C(=O)Nc(c12)cccc2 |  |
| benzimidazole-4-CN-2-CH2NHMe |  | pyrimidine-2-Me-4-OMe |
| N#Cc1cccc(c12)nc([nH]2)CNC | indolinone-3-CH2-CN2 | Cc1nc(ccn1)OC |
|  | N#CC(C#N)=C1C(=O)Nc(c12)cccc2 |  |
| benzimidazole-4-CN-2-CH2OH-5-iPr |  |  |
| N#Cc1c(C(C)C)ccc(c12)[nH]c(n2)CO | indolinone-3-NH2-3-Me_Prot_A | pyrimidine-2-NH2 |
|  | CC1([NH3+])C(=O)Nc(c12)cccc2 | Nc1ncccn1 |
| benzimidazole-4-CN-2-CH2OH-5-iPr-7-Me |  |  |
| N#Cc1c(C(C)C)cc(C)c(c12)[nH]c(n2)CO | indolinone-3-NH2-3-Me_Prot_B | pyrimidine-2-NH2-4-OMe |
|  | CC1([NH3+])C(=O)Nc(c12)cccc2 | COc1ccnc(n1)N |
| benzimidazole-4-CN-2-Et-5-CF3-7Me |  |  |
| N#Cc1c(C(F)(F)F)cc(C)c(c12)[nH]c(n2)CC | indolinone-3-NH2_Prot_A | pyrimidine-2-NHMe |
|  | [NH3+]C1C(=O)Nc(c12)cccc2 | CNc1ncccn1 |
|  |  |  |
| benzimidazole-4-CN-2-Et-5-iPr | indolinone-3-NH2_Prot_B | pyrimidine-2-OMe |
| N#Cc1c(C(C)C)ccc(c12)[nH]c(n2)CC | [NH3+]C1C(=O)Nc(c12)cccc2 | COc1ncccn1 |
|  |  |  |
| benzimidazole-4-CN-2-Et-5-iPr-7-CCMe | indolinone-6-CN | pyrimidine-2-OMe-4-NH2 |
| N#Cc1c(C(C)C)cc(C#CCC)c(c12)[nH]c(n2)CC | c1cc(C#N)cc(c12)NC(=O)C2 | COc(n1)nccc1N |
|  |  |  |
| benzimidazole-4-CN-2-Et-5-Me-7-CCMe | indolinone-6-Me | pyrimidine-2-SMe |
| N#Cc1c(C)cc(C#CCC)c(c12)[nH]c(n2)CC | c1cc(C)cc(c12)NC(=O)C2 | CSc1ncccn1 |
|  |  |  |
| benzimidazole-4-CN-2-Me-5-iPr-7-CCMe | indolinone-6-OH | pyrimidine-4-Cl |
| CCC#Cc(c(c12)[nH]c(n2)C)c(C(C)C)cc1C#N | C1C(=O)Nc(c12)cc(O)cc2 | Clc1ccncn1 |
|  |  |  |
| benzimidazole-4-CN-2-Me-5-iPr-7-Et | indolizine | pyrimidine-4-Me |
| N#Cc1c(C(C)C)cc(CC)c(c12)[nH]c(n2)C | c1ccn(c12)cccc2 | Cc1ccncn1 |
|  |  |  |
| benzimidazole-4-CN-2-Me-5-OMe-7-CCMe | isobutamide | pyrimidine-4-NH2 |
| Cc(n1)[nH]c(c12)c(C#CC)cc(c2C#N)OC | NC(=O)C(C)C | Nc1ccncn1 |
|  |  |  |
| benzimidazole-4-CN-5-1-MeOEt-2-Et-7-Me |  | pyrimidine-24-diMe |
| COCc(c1C#N)cc(C)c(c12)[nH]c(n2)CC | isoindoline | Cc1ccnc(n1)C |
|  | C1NCc(c12)cccc2 |  |
| benzimidazole-4-CN-5-1-MeOEt-27-diMe |  | pyrimidine-24-diNH2 |
| COC(C)c(c1C#N)cc(C)c(c12)[nH]c(n2)C | isoindoline-N-Me | Nc1ccnc(n1)N |
|  | CN(C1)Cc(c12)cccc2 |  |
| benzimidazole-4-CN-5-Ac-2-CH2OH-7-Me |  | pyrimidine-24-diNH2-6-Et_Prot |
| N#Cc1c(C(=O)C)cc(C)c(c12)[nH]c(n2)CO | isoindoline_Prot | CCC1=CC(N)=NC(N)N1 |
|  | C1[NH2+]Cc(c12)cccc2 |  |
| benzimidazole-4-CN-5-Ac-2-Et-7Me |  | pyrimidine-24-diNH2-6-Me_Prot |
| N#Cc1c(C(=O)C)cc(C)c(c12)[nH]c(n2)CC | isoprene | CC1=CC(N)=NC(N)N1 |
|  | CC(C)=CC |  |
| benzimidazole-4-CN-5-Ac-27-diMe |  | pyrimidine-24-diNH2-6-OMe_Prot |
| N#Cc1c(C(=O)C)cc(C)c(c12)[nH]c(n2)C | isoquinolin-1-one-N-Me | COC1=CC(N)N=C(N)N1 |
|  | O=c1n(C)ccc(c12)cccc2 |  |
| benzimidazole-4-CN-5-CF3-27-diMe |  | pyrimidine-24-diNH2-6-SMe_Prot_A |
| N#Cc1c(C(F)(F)F)cc(C)c(c12)[nH]c(n2)C | isoquinolin-3-one | CSC1=CC(N)N=C(N)N1 |
|  | c1cccc(c12)c[nH]c(=O)c2 |  |
| benzimidazole-4-CN-5-Et-7-Me |  | pyrimidine-24-diNH2_Prot |
| N#Cc1c(CC)cc(C)c(c12)[nH]cn2 | isoquinoline | NC1=NC(N)C=CN1 |
|  | c1cncc(c12)cccc2 |  |
| benzimidazole-4-CN-5-Me-7-Et |  | pyrimidine-24-diOMe_AntiA |
| N#Cc1c(C)cc(CC)c(c12)[nH]cn2 | isoquinoline-5-CN-3-Et | COc1ccnc(n1)OC |
|  | N#Cc1cccc(c12)cnc(c2)CC |  |
| benzimidazole-4-CN-5-MeOMe-27-diMe |  | pyrimidine-24-diOMe_AntiB |
| COCc(c1C#N)cc(C)c(c12)[nH]c(n2)C | isoquinoline-decahydro-4-OH_Prot | COc1ccnc(n1)OC |
|  | C1C[NH2+]CC(C12O)CCCC2 |  |
| benzimidazole-4-CN-5-OMe-7-CCEt |  | pyrimidine-24-diOMe_SynA |
| CCC#Cc(c(c12)[nH]cn2)cc(c1C#N)OC | isoquinoline-tetrahydro-6-OH-7-Me_Prot | COc1ccnc(n1)OC |
|  | COc(c(c1)O)cc(c12)C[NH2+]CC2 |  |
| benzimidazole-4-CN-5-OMe-7-CCMe |  | pyrimidine-24-diOMe_SynB |
| n1c[nH]c(c12)c(C#CC)cc(c2C#N)OC | isoquinoline-tetrahydro-N-Me_Prot | COc1ccnc(n1)OC |
|  | C1C[NH+](C)Cc(c12)cccc2 |  |
| benzimidazole-4-CN-5-OMe-7-Me |  | pyrimidine-26-diNH2-4-OMe_Prot |
| N#Cc1c(OC)cc(C)c(c12)[nH]cn2 | isoquinoline-tetrahydro_Prot_A | COC1=CC(N)NC(=N1)N |
|  | C1C[NH2+]Cc(c12)cccc2 |  |
| benzimidazole-4-CN-5-OMe_90degrees |  | pyrimidine-46-diNH2-2-Me_Prot |
| N#Cc1c(OC)ccc(c12)[nH]cn2 | isoquinoline-tetrahydro_Prot_B | NC1=NC(C)NC(=C1)N |
|  | C1C[NH2+]Cc(c12)cccc2 |  |
| benzimidazole-4-CN-5-tBu-2-Et |  | pyrimidine-46-diNH2-2-OMe_Prot |
| N#Cc1c(C(C)(C)C)ccc(c12)[nH]c(n2)CC | isothiazole-5-NH2-3-CONHMe_anti | CNC1=NC(OC)NC(=C1)N |
|  | CNC(=O)c1cc(N)sn1 |  |
| benzimidazole-4-CN-7-CCEt |  | pyrimido-12a-pyrimidine-hexahydro_Prot |
| CCC#Cc(c(c12)[nH]cn2)ccc1C#N | isothiazole-11-dioxide | [NH+]1CCCN(C=12)CCCN2 |
|  | N1=CC=CS1(=O)=O |  |
| benzimidazole-4-CN-7-CCMe |  | pyrrol-2-one-1-Me-4-NHMe |
| n1c[nH]c(c12)c(C#CC)ccc2C#N | isoxazol-3-one-5-NHMe-2-Me | CNC1=CC(=O)N(C)C1 |
|  | CNc1cc(=O)n(o1)C |  |
| benzimidazole-4-CN-7-NH2-2-Et |  | pyrrol-2-one-1-Me-4-NMe2 |
| Nc1ccc(C#N)c(c12)[nH]c(n2)CC | isoxazol-5-one-3-Me | CN(C)C1=CC(=O)N(C)C1 |
|  | Cc1cc(=O)o[nH]1 |  |
| benzimidazole-4-CN-25-diEt-7-CCMe |  | pyrrole |
| N#Cc1c(C(C)C)cc(CC)c(c12)[nH]c(n2)CC | isoxazole | c1cc[nH]c1 |
|  | c1cnoc1 |  |
| benzimidazole-4-CN-25-diMe-7-CCMe |  | pyrrole-1-Me |
| CCC#Cc(c(c12)[nH]c(n2)C)cc(C)c1C#N | isoxazole-3-CH2CN_A | Cn1cccc1 |
|  | N#CCc1ccon1 |  |
| benzimidazole-4-CN-27-diMe-5-iPr |  | pyrrole-2-NHMe-3-CN |
| N#Cc1c(C(C)C)cc(C)c(c12)[nH]c(n2)C | isoxazole-3-CH2OMe_A | N#Cc1c(NC)[nH]cc1 |
|  | COCc1ccon1 |  |
| benzimidazole-4-CN-257-triMe |  | pyrrole-2-NHMe-3-NO2 |
| N#Cc1c(C)cc(C)c(c12)[nH]c(n2)C | isoxazole-3-CH2OMe_B | [O-][N+](=O)c1c(NC)[nH]cc1 |
|  | COCc1ccon1 |  |
| benzimidazole-4-CONH2 |  | pyrrole-3-Ac |
| NC(=O)c1cccc(c12)[nH]cn2 | isoxazole-3-CHMeCN_A | CC(=O)c1cc[nH]c1 |
|  | N#CC(C)c1ccon1 |  |
| benzimidazole-4-CONH2-2-p-phenol |  | pyrrole-3-Cl |
| NC(=O)c1cccc(c12)[nH]c(n2)-c3ccc(O)cc3 | isoxazole-3-CHMeCN_C | Clc1cc[nH]c1 |
|  | N#CC(C)c1ccon1 |  |
| benzimidazole-4-CONH2-2-p-phenol_36-deg |  | pyrrole-3-CN |
| NC(=O)c1cccc(c12)[nH]c(n2)-c3ccc(O)cc3 | isoxazole-3-CHMeCN_D | N#Cc1cc[nH]c1 |
|  | N#CC(C)c1ccon1 |  |
| benzimidazole-4CN-5-OMe-7-CCimidazoleNMe_25deg |  |  |
| n1cn(C)cc1C#Cc(c(c23)[nH]cn3)cc(c2C#N)OC | isoxazole-3-CHMeOMe_A | pyrrole-3-Et_A |
|  | COC(C)c1ccon1 | CCc1cc[nH]c1 |
| benzimidazole-4CN-5-OMe-7-CCisoxazole3Me_25deg |  |  |
| o1nc(C)cc1C#Cc(c(c23)[nH]cn3)cc(c2C#N)OC | isoxazole-3-CHMeOMe_B | pyrrole-3-Et_B |
|  | COC(C)c1ccon1 | CCc1cc[nH]c1 |
| benzimidazole-4CN-5-OMe-7-CCtriazoleNMe_25deg |  |  |
| n1nn(C)cc1C#Cc(c(c23)[nH]cn3)cc(c2C#N)OC | isoxazole-3-CHMeOMe_C | pyrrole-3-iPr_A |
|  | COC(C)c1ccon1 | CC(C)c1cc[nH]c1 |
|  |  |  |
| benzimidazole-5-Cl | isoxazole-3-CHMeOMe_D | pyrrole-3-iPr_B |
| n1c[nH]c(c12)ccc(Cl)c2 | COC(C)c1ccon1 | CC(C)c1cc[nH]c1 |
|  |  |  |
| benzimidazole-5-CN | isoxazole-3-CMe2CN_A | pyrrole-3-Me |
| N#Cc(c1)ccc(c12)[nH]cn2 | N#CC(C)(C)c1ccon1 | Cc1cc[nH]c1 |
|  |  |  |
| benzimidazole-5-CN-2-4-pyrimidyl_A | isoxazole-3-CMe2OMe_A | pyrrole-3-NHMe_glb_A |
| N#Cc(c1)ccc(c12)[nH]c(n2)-c3ccncn3 | COC(C)(C)c1ccon1 | CNc1cc[nH]c1 |
|  |  |  |
| benzimidazole-5-CN-2-4-pyrimidyl_B | isoxazole-3-CMe2OMe_B | pyrrole-3-NHMe_glb_B |
| N#Cc(c1)ccc(c12)[nH]c(n2)-c3ccncn3 | COC(C)(C)c1ccon1 | CNc1cc[nH]c1 |
|  |  |  |
| benzimidazole-5-CN-2-CF3 | isoxazole-3-CN | pyrrole-3-NMe2 |
| N#Cc(c1)ccc(c12)[nH]c(n2)C(F)(F)F | N#Cc1ccon1 | CN(C)c1cc[nH]c1 |
|  |  |  |
| benzimidazole-5-CN-2-Et_A | isoxazole-3-Et_A | pyrrole-3-OH_A |
| N#Cc(c1)ccc(c12)[nH]c(n2)CC | CCc1ccon1 | Oc1cc[nH]c1 |
|  |  |  |
| benzimidazole-5-CN-2-Et_B |  | pyrrole-3-OH_B |
| N#Cc(c1)ccc(c12)[nH]c(n2)CC | isoxazole-3-Et_B | Oc1cc[nH]c1 |
|  | CCc1ccon1 |  |
| benzimidazole-5-CN-2-Me |  | pyrrole-3-OMe_glb_A |
| N#Cc(c1)ccc(c12)[nH]c(n2)C | isoxazole-3-iPr_A | COc1cc[nH]c1 |
|  | CC(C)c1ccon1 |  |
| benzimidazole-5-CN-2-tBu |  | pyrrole-3-OMe_glb_B |
| N#Cc(c1)ccc(c12)[nH]c(n2)C(C)(C)C | isoxazole-3-iPr_B | COc1cc[nH]c1 |
|  | CC(C)c1ccon1 |  |
| benzimidazole-5-CN-6-CH2OMe |  | pyrrolidin-2-one |
| COCc(c(C#N)c1)cc(c12)[nH]cn2 | isoxazole-3-Me | O=C1CCCN1 |
|  | Cc1ccon1 |  |
| benzimidazole-5-CN-6-Et_A |  | pyrrolidin-2-one-N-Me |
| N#Cc(c1)c(CC)cc(c12)[nH]cn2 | isoxazole-3-NH2 | CN1CCCC1=O |
|  | Nc1ccon1 |  |
| benzimidazole-7-CCMe |  | pyrrolidin-5-one-1-Me-3-NH2_Prot_R |
| CC#Cc1cccc(c12)nc[nH]2 | isoxazole-3-NH2-4-Et-5-Me_90deg | CN1CC([NH3+])CC1=O |
|  | CCc1c(C)onc1N |  |
| benzimidazole-7-Me |  | pyrrolidin-5-one-1-Me-3-NH2_Prot_S |
| Cc1cccc(c12)nc[nH]2 | isoxazole-3-NH2-4-Me | CN1CC([NH3+])CC1=O |
|  | Cc1c(N)noc1 |  |
| benzimidazole-45-diCl |  | pyrrolidine |
| n1c[nH]c(c12)ccc(Cl)c2Cl | isoxazole-3-NH2-5-Me | C1CCNC1 |
|  | Cc1cc(N)no1 |  |
| benzimidazole-N-Et_B |  | pyrrolidine-NMe_Prot |
| CCn(cn1)c(c12)cccc2 | isoxazole-3-NH2-45-diMe | C[NH+]1CCCC1 |
|  | Cc1c(C)onc1N |  |
| benzimidazole-N-Me |  | pyrrolidine_Prot |
| n1cn(C)c(c12)cccc2 | isoxazole-3-NHMe_A | C1CC[NH2+]C1 |
|  | CNc1ccon1 |  |
| benzimidazole4-CN-2-Et-5-iPr_B |  | pyrrolidine_Prot_B |
| N#Cc1c(C(C)C)ccc(c12)[nH]c(n2)CC | isoxazole-3-NHMe_B | C1CC[NH2+]C1 |
|  | CNc1ccon1 |  |
| benzimidazolin-2-one-5-CN |  | pyrrolo-32c-pyridine-3-SOMe |
| N#Cc(c1)ccc(c12)[nH]c(=O)[nH]2 | isoxazole-3-NMe2 | CS(=O)c1c[nH]c(c12)ccnc2 |
|  | CN(C)c1ccon1 |  |
| benzimidazolone-5-CN |  | quinazolin-2-one-1-Me_dihydro_B |
| N#Cc(c1)ccc(c12)[nH]c(=O)[nH]2 | isoxazole-3-OMe_glb | C1NC(=O)N(C)c(c12)cccc2 |
|  | COc1ccon1 |  |
| benzisoxazole |  | quinazolin-2-one-1H-4-NH2 |
| c1noc(c12)cccc2 | isoxazole-3-OMe_high | c1cccc(c12)[nH]c(=O)nc2N |
|  | COc1ccon1 |  |
| benzisoxazole-3-Me |  | quinazolin-2-one-3H |
| Cc1noc(c12)cccc2 | isoxazole-5-NH2-3-CONHMe_anti | c1[nH]c(=O)nc(c12)cccc2 |
|  | CNC(=O)c1cc(N)on1 |  |
| benzisoxazole-3-NH2 |  | quinazolin-2-one-6-NHMe |
| Nc1noc(c12)cccc2 | isoxazole-23-diMe | CNc(c1)ccc(c12)[nH]c(=O)nc2 |
|  | Cc1cc(C)on1 |  |
| benzo-45-azepino-12a-indole-2-CN-6-oxo_dihydro |  | quinazolin-2-one-7-NMe2_24deg |
| N#Cc(c1)ccc(c12)n3c(c2)Cc4c(CC3=O)cccc4 | isoxazoline_A | CN(C)c(cc1)cc(c12)[nH]c(=O)nc2 |
|  | C1=NOCC1 |  |
| benzo-45-cyclohepta-12-45-furo-23c-pyridine_dihydro |  | quinazolin-4-one |
| c1cncc2oc(c3c12)Cc4c(CC3)cccc4 | isoxazoline_B | c1cccc(c12)nc[nH]c2=O |
|  | C1=NOCC1 |  |
| benzo-45-cyclohepta-12-45-pyrrolo-23c-pyridine_tetrahydro |  | quinazolin-4-one-1-Me-2-NH2 |
| c1cncc(c12)nc3n2CCc4c(C3)cccc4 | isoxazolo-54c-tetrahydropyridine-3-Me_Prot_A | OC1N=C(N)N(C)C(C=12)=CCC=C2 |
|  | Cc1noc(c12)C[NH2+]CC2 |  |
| benzo-45-cyclohepta-12-45-thieno-23c-pyridine_dihydro |  | quinazolin-4-one-1-Me-2-NH2-7-OMe |
| c1cncc2sc(c3c12)Cc4c(CC3)cccc4 | isoxazolo-54c-tetrahydropyridine-3-Me_Prot_B | OC1N=C(N)N(C)c(c12)cc(cc2)OC |
|  | Cc1noc(c12)C[NH2+]CC2 |  |
| benzo-45-imidazo-12a-azepine-3-CN_tetrahydro |  | quinazolin-4-one-1-Me-5-F |
| N#Cc(c1)ccc(c12)n3c(n2)CCCCC3 | malonitrile | OC1N=CN(C)c(c12)cccc2F |
|  | N#CCC#N |  |
| benzo-45-imidazo-12a-azepine-3-CONHMe_tetrahydro_B |  | quinazolin-4-one-2-amino-5678-tetrahydro_A |
| CNC(=O)c(c1)ccc(c12)n3c(n2)CCCCC3 | methane | C1CCCC(=C12)NC(N)=NC2O |
|  | [C] |  |
| benzo-45-imidazo-12a-azepine-3-COO_anion |  | quinazolin-4-one-2-amino-5678-tetrahydro_B |
| O=C(O)c(c1)ccc(c12)n3c(n2)CCCCC3 | methanesulfonamide | C1CCCC(=C12)NC(N)=NC2O |
|  | CS(=O)(=O)N |  |
| benzo-67-azepino-43b-indole-3-CN-11-Ac_tetrahydro |  | quinazolin-4-one-2-NH2 |
| c1cc(C#N)cc2[nH]c(c3c12)Cc4c(N(C3)C(=O)C)cccc4 | methanesulfonamide-N-diMe | c1cccc(c12)NC(N)N=C2O |
|  | CN(C)S(=O)(=O)C |  |
| benzo-67-azepino-43b-indole-3-CN-11-Me_tetrahydro |  |  |
| c1cc(C#N)cc2[nH]c(c3c12)Cc4c(N(C)C3)cccc4 | methanesulfonamide-N-Me_A | quinazolin-4-one-2-NH2-1-Me-5678tetrahydro_A |
|  | CNS(=O)(=O)C | OC1N=C(N)N(C)C(=C12)CCCC2 |
| benzo-67-oxepino-43b-indole-9-CN_dihydro |  |  |
| c1cc(C#N)cc2[nH]c(c3c12)Cc4c(OC3)cccc4 | methanesulfonamide-N-Me_B | quinazolin-4-one-3-Me |
|  | CNS(=O)(=O)C | OC1N=CN(C)C(C=12)=CCC=C2 |
| benzo-b-benzofuro-23e-azepine-3-CN-11-Me_dihydro |  |  |
| c1cc(C#N)cc2oc(c3c12)Cc4c(N(C)C3)cccc4 | methanesulfonamide_A | quinazolin-4-one-34-dihydro-3-Me-5-CN |
|  | CS(=O)(=O)N | N#Cc1cccc(c12)ncn(C)c2=O |
| benzo-b-benzofuro-23e-azepine-3-CN-11-Me_dihydro_MMFF94 |  |  |
| c1cc(C#N)cc2oc(c3c12)Cc4c(N(C)C3)cccc4 | methanethiolate | quinazoline |
|  | CS | c1ncnc(c12)cccc2 |
| benzo-b-pyrido-43-45-pyrrolo-23e-azepin-6-Ac |  |  |
| c1cccc(N(C2)C(=O)C)c1Cc(c2c34)[nH]c3cncc4 | methanol | quinazoline-2-NH2 |
|  | CO | c1nc(N)nc(c12)cccc2 |
| benzo-b-pyrido-43-45-pyrrolo-23e-azepine-6-Me_tetrahydro |  |  |
| c1cccc(N(C)C2)c1Cc(c2c34)[nH]c3cncc4 | methyl_acetate | quinazoline-2-NH2-6-CN |
|  | CC(=O)OC | N#Cc(c1)ccc(c12)nc(N)nc2 |
| benzo-d-benzo-45-imidazo-12a-azepine-3-CN-12-oxo |  |  |
| N#Cc(c1)ccc(c12)n3c(n2)Cc4c(CC3=O)cccc4 | methyl_acetate_A | quinazoline-2-NH2-7-CN |
|  | CC(=O)OC | c1cc(C#N)cc(c12)nc(N)nc2 |
| benzo-d-benzo-45-imidazo-12a-azepine-3-CN_dihydro |  |  |
| N#Cc(c1)ccc(c12)n3c(n2)Cc4c(CC3)cccc4 | methyl_acetate_B | quinazoline-4-CN |
|  | CC(=O)OC | N#Cc1ncnc(c12)cccc2 |
| benzo-d-pyrido-34-45-imidazo-12a-azepine_dihydro |  |  |
| c1cncc2[nH]c(c3c12)Cc4c(CC3)cccc4 | methyl_acetate_syn | quinazoline-4-CN-2-Et |
|  | CC(=O)OC | N#Cc1nc(CC)nc(c12)cccc2 |
| benzo-e-124-thiadiazine-dioxide-4-Me |  |  |
| CN1C=NS(=O)(=O)c(c12)cccc2 |  | quinazoline-4-CN-2-Me |
|  | methyl_carbamate | N#Cc1nc(C)nc(c12)cccc2 |
| benzo-f-51c-imidazo-14-oxazepine-511-dihydro-3-NH2_Prot | NC(=O)OC |  |
| C1NC(N)N(C=12)Cc3c(OC2)cccc3 |  | quinazoline-4-CN-7-OMe |
|  | methylamine | N#Cc1ncnc(c12)cc(cc2)OC |
| benzoate | CN |  |
| O=C(O)c1ccccc1 |  | quinazoline-6-CN |
|  | methylamine_eclipsed_Prot | N#Cc(c1)ccc(c12)ncnc2 |
| benzodiazepine-13-diMe | C[NH3+] |  |
| O=C1NC(C)C(=O)N(C)c(c12)cccc2 |  | quinazoline-45-dione-2-NH2-1-Me-78dihydro_A |
|  | methylamine_Prot | OC1N=C(N)N(C)C(C=12)CCCC2=O |
| benzodiazepine-25-dione-4-Me_A | C[NH3+] |  |
| O=C1N(C)CC(=O)Nc(c12)cccc2 |  | quinolin-2-one |
|  | methylcarbamate-OMe | c1cccc(c12)[nH]c(=O)cc2 |
| benzodioxine | COC(=O)NC |  |
| O1CCOc(c12)cccc2 |  | quinolin-2-one-1-Et |
|  | methylethanamine | c1cc(=O)n(CC)c(c12)cccc2 |
| benzodioxole | CCNC |  |
| O1COc(c12)cccc2 |  | quinolin-2-one-1-Et_up |
|  | morpholine | c1cc(=O)n(CC)c(c12)cccc2 |
| benzodioxole-4-CN | C1COCCN1 |  |
| N#Cc1cccc(c12)OCO2 |  | quinolin-2-one-1-Me |
|  | morpholine-N-Ac | c1cc(=O)n(C)c(c12)cccc2 |
| benzodioxole-4-OH | CC(=O)N1CCOCC1 |  |
| O1COc(c12)cccc2O |  | quinolin-2-one-3-Me |
|  | morpholine-N-Ac_B | Cc(c1)c(=O)[nH]c(c12)cccc2 |
| benzofuran | CC(=O)N1CCOCC1 |  |
| c1coc(c12)cccc2 |  | quinolin-2-one-NEt-5-OMe-3-COO_minus |
|  | morpholine-N-Me | O=C(O)c(c1)c(=O)n(CC)c(c12)cccc2OC |
|  | CN1CCOCC1 |  |
| benzofuran-2-Me |  | quinolin-2-one-NEt-5-OMe_A |
| Cc(c1)oc(c12)cccc2 | morpholine-N-Me_Prot | c1cc(=O)n(CC)c(c12)cccc2OC |
|  | C[NH+]1CCOCC1 |  |
| benzofuran-3-Me |  | quinolin-2-one-NMe-5-OMe-3-COO_minus |
| Cc1coc(c12)cccc2 | morpholine_Prot | O=C(O)c(c1)c(=O)n(C)c(c12)cccc2OC |
|  | C1COCC[NH2+]1 |  |
| benzofuran-3-NO2 |  | quinolin-4-one |
| c1cccc(c12)occ2[N+]([O-])=O | naphthalene | c1cccc(c12)NC=CC2O |
|  | c1cccc(c12)cccc2 |  |
| benzoisothiazole |  | quinolin-4-one-2-Et |
| c1nsc(c12)cccc2 | naphthalene-2-1-EtNH2_S_Prot | OC1C=C(CC)NC(C=12)=CC=CC2 |
|  | CC([NH3+])c(c1)ccc(c12)cccc2 |  |
| benzonitrile |  | quinolin-4-one-2-Et-6-CH2SO2NH2_A |
| N#Cc1ccccc1 | naphthalene-2-Me | OC1=CC(CC)Nc(c12)ccc(c2)COS(=O)(=O)N |
|  | Cc(c1)ccc(c12)cccc2 |  |
| benzonitrile-2-Cl |  | quinolin-4-one-2-Et-6-CH2SO2NH2_B |
| N#Cc1c(Cl)cccc1 | naphthalene-2-Me-6-OMe_A | OC1=CC(CC)Nc(c12)ccc(c2)COS(=O)(=O)N |
|  | Cc(c1)ccc(c12)cc(cc2)OC |  |
| benzonitrile-2-Cl-3-Me |  | quinolin-4-one-2-Et-6-OMe_Ar |
| N#Cc1c(Cl)c(C)ccc1 | naphthalene-2-Me-6-OMe_B | OC1=CC(CC)Nc(c12)ccc(c2)OC |
|  | Cc(c1)ccc(c12)cc(cc2)OC |  |
| benzonitrile-2-Cl-34-diMe |  | quinolin-4-one-2-Et-6-OMe_Ar_down |
| N#Cc1c(Cl)c(C)c(C)cc1 | naphthalene-26-diMe | OC1=CC(CC)Nc(c12)ccc(c2)OC |
|  | Cc(c1)ccc(c12)cc(C)cc2 |  |
| benzonitrile-2-Cl-35-diMe |  | quinolin-4-one-2-Et-6-OSO2NH2 |
| N#Cc1c(Cl)c(C)cc(C)c1 | naphthyridine-16-45-dione-2-NH2-1-Me-78-dihydro_A | OC1=CC(CC)Nc(c12)ccc(c2)OS(=O)(=O)N |
|  | OC1C=C(N)N(C)C(C=12)CCNC2=O |  |
| benzonitrile-2-F |  | quinolin-4-one-2-Et-6-SO2Me |
| N#Cc1c(F)cccc1 | neopentane | OC1=CC(CC)Nc(c12)ccc(c2)OS(=O)(=O)C |
|  | CC(C)(C)C |  |
| benzonitrile-2-F-3-Me |  | quinolin-4-one-8-propyne |
| N#Cc1c(F)c(C)ccc1 | nitrobenzene | CC#Cc(ccc1)c(c12)NCC=C2O |
|  | [O-][N+](=O)c1ccccc1 |  |
| benzonitrile-2-F-3-Me-5-OMe_A |  | quinolin-8-one-2-OMe-67-dihydro |
| N#Cc1c(F)c(C)cc(c1)OC | nitromethane | COc(n1)ccc(c12)CCCC2=O |
|  | C[N+]([O-])=O |  |
| benzonitrile-2-F-3-Me-5-OMe_B |  | quinolin-8-one-67-dihydro_B |
| N#Cc1c(F)c(C)cc(c1)OC | norbornane | n1cccc(c12)CCCC2=O |
|  | C12CC(CC1)CC2 |  |
| benzonitrile-2-F-5-Cl |  | quinoline |
| N#Cc1c(F)ccc(Cl)c1 | octahydropyrido-12a-pyrazine-7-Me_Prot | c1ccnc(c12)cccc2 |
|  | C1CC(C)CN(C12)CC[NH2+]C2 |  |
| benzonitrile-2-F-34-diMe |  |  |
| N#Cc1c(F)c(C)c(C)cc1 | octahydropyrido-12a-pyrazine_S_Prot | quinoline-2-Me |
|  | C1[NH2+]CCN(C12)CCCC2 | c1cc(C)nc(c12)cccc2 |
| benzonitrile-2-F-35-diMe |  |  |
| N#Cc1c(F)c(C)cc(C)c1 | octahydropyrrolo-12a-pyrazine-6-oxo_Prot_A | quinoline-2-NH2 |
|  | C1CC(=O)N(C12)CC[NH2+]C2 | c1cccc(c12)nc(N)cc2 |
| benzonitrile-2-Me |  |  |
| N#Cc1c(C)cccc1 | oxadiazole | quinoline-2-NHMe-3-Me |
|  | c1nnco1 | Cc(c1)c(NC)nc(c12)cccc2 |
| benzonitrile-2-Me-4-OH_A |  |  |
| N#Cc1c(C)cc(O)cc1 | oxadiazole-2-Me | quinoline-2-OMe |
|  | Cc1nnco1 | COc(cc1)nc(c12)cccc2 |
| benzonitrile-2-NO2-3-NH2-6-OMe-4-CCMe_A |  |  |
| COc1cc(C#CC)c(N)c([N+]([O-])=O)c1C#N | oxadiazole-2-NHMe-5-Me_A | quinoline-3-Cl |
|  | Cc1nnc(o1)NC | c1cccc(c12)ncc(Cl)c2 |
| benzonitrile-2-NO2-3-NH2-6-OMe-4-CCMe_B |  |  |
| COc1cc(C#CC)c(N)c([N+]([O-])=O)c1C#N | oxadiazole-2-NHMe-5-Me_B | quinoline-3-Cl-6-NH2 |
|  | Cc1nnc(o1)NC | c1c(Cl)cnc(c12)ccc(c2)N |
| benzonitrile-3-Br |  |  |
| N#Cc1cc(Br)ccc1 | oxadiazole-124 | quinoline-3-Me |
|  | c1ncon1 | Cc(c1)cnc(c12)cccc2 |
| benzonitrile-3-Me |  |  |
| N#Cc1cc(C)ccc1 | oxadiazole-125-3-NH2-4-Me | quinoline-4-OH |
|  | Cc1c(N)non1 | c1cccc(c12)nccc2O |
| benzonitrile-3-NH2 |  |  |
| N#Cc1cc(N)ccc1 | oxadiazole-134-5-NH2-2-carboxamide | quinoline-5-CN |
|  | NC(=O)c1nnc(o1)N | N#Cc1cccc(c12)nccc2 |
| benzonitrile-4-Et |  |  |
| N#Cc1ccc(CC)cc1 |  | quinoline-5-CN-2-Et |
|  | oxazepane-14-4-Me | N#Cc1cccc(c12)nc(CC)cc2 |
| benzonitrile-4-NH2 | CN1CCCOCC1 |  |
| N#Cc1ccc(N)cc1 |  | quinoline-5-CN-3-Et |
|  | oxazole | N#Cc1cccc(c12)ncc(c2)CC |
| benzonitrile-4-OH | c1cocn1 |  |
| N#Cc1ccc(O)cc1 |  | quinoline-6-1-EtNH2_S_Prot |
|  | oxazole-2-CH2CN_glb_A | CC([NH3+])c(c1)ccc(c12)nccc2 |
| benzonitrile-23-diNH2-6-F | N#CCc1ncco1 |  |
| N#Cc1c(N)c(N)ccc1F |  | quinoline-7-Me |
|  | oxazole-2-CH2CN_glb_B | c1cc(C)cc(c12)nccc2 |
| benzonitrile-23-diNH2-6-OMe | N#CCc1ncco1 |  |
| N#Cc1c(N)c(N)ccc1OC |  | quinoline-45-dione-2-NH2-1-Me-78dihydro_A |
|  | oxazole-2-CH2CN_med | OC1C=C(N)N(C)C(C=12)CCCC2=O |
| benzonitrile-23-diNH2-6-OMe-4-CCMe | N#CCc1ncco1 |  |
| COc1cc(C#CC)c(N)c(N)c1C#N |  | quinone |
|  | oxazole-2-CH2OH_glb_A | O=C1C=CC(=O)C=C1 |
| benzonitrile-24-diCl | OCc1ncco1 |  |
| N#Cc1c(Cl)cc(Cl)cc1 |  | quinoxaline |
|  | oxazole-2-CH2OH_glb_B | n1ccnc(c12)cccc2 |
| benzonitrile-25-diCl | OCc1ncco1 |  |
| N#Cc1c(Cl)ccc(Cl)c1 |  | quinoxaline-34-dihydro-2-one-14-diMe |
|  | oxazole-2-CH2OMe_A | CN1C(=O)CN(C)c(c12)cccc2 |
| benzonitrile-26-diCl | COCc1ncco1 |  |
| N#Cc1c(Cl)cccc1Cl |  | quinuclidine |
|  | oxazole-2-CH2OMe_B | C12CCN(CC1)CC2 |
| benzonitrile-26-diCl-3-Me | COCc1ncco1 |  |
| N#Cc1c(Cl)c(C)ccc1Cl |  | quinuclidine_Prot |
|  | oxazole-2-CHMeCN_glb_A | C12CC[NH+](CC1)CC2 |
|  | N#CC(C)c1ncco1 |  |
| benzonitrile-26-diCl-34-diMe |  | ribose-15-dideoxy |
| N#Cc1c(Cl)c(C)c(C)cc1Cl | oxazole-2-CHMeCN_glb_B | CC1C(O)C(CO1)O |
|  | N#CC(C)c1ncco1 |  |
| benzothiazole |  | rimantadine_Prot |
| n1csc(c12)cccc2 | oxazole-2-CHMeOMe_glb | C[NH2+]C12CC3CC(C1)CC(C2)C3 |
|  | COC(C)c1ncco1 |  |
| benzothiazole-2-CF3-6-1-EtNH2_S_Prot |  | spiro-35-nonane-2-oxa-7-aza_Prot_A |
| FC(F)(F)c(n1)sc(c12)cc(C(C)[NH3+])cc2 | oxazole-2-CHMeOMe_med | C1OCC12CC[NH2+]CC2 |
|  | COC(C)c1ncco1 |  |
| benzothiazole-2-CN |  | spiro-35-nonane-2-oxa-7-aza_Prot_B |
| N#Cc(n1)sc(c12)cccc2 | oxazole-2-CMe2CN_glb_A | C1OCC12CC[NH2+]CC2 |
|  | N#CC(C)(C)c1ncco1 |  |
| benzothiazole-2-Me |  | spirobenzofuran-34p-piperidine_Prot_A |
| Cc(n1)sc(c12)cccc2 | oxazole-2-CMe2CN_glb_B | c1cccc(OC2)c1C23CC[NH2+]CC3 |
|  | N#CC(C)(C)c1ncco1 |  |
| benzothiazole-2-Me-6-1-EtNH2_S_Prot |  | spirobenzofuran-34p-piperidine_Prot_B |
| CC([NH3+])c(cc1)cc(c12)sc(n2)C | oxazole-2-CMe2CN_glb_C | c1cccc(OC2)c1C23CC[NH2+]CC3 |
|  | N#CC(C)(C)c1ncco1 |  |
| benzothiazole-2-MeO-6-1-EtNH2_S_Prot |  | squaramide |
| CC([NH3+])c(cc1)cc(c12)sc(n2)OC | oxazole-2-CMe2OMe_A | Nc1c(N)c(=O)c1=O |
|  | COC(C)(C)c1ncco1 |  |
| benzothiazole-2-NH2 |  | sulfamate-OMe_B |
| Nc(n1)sc(c12)cccc2 | oxazole-2-CMe2OMe_B | COS(=O)(=O)N |
|  | COC(C)(C)c1ncco1 |  |
| benzothiazole-2-tbu-6-1-EtNH2_S_Prot |  | t-butanol |
| CC(C)(C)c(n1)sc(c12)cc(C(C)[NH3+])cc2 | oxazole-2-Me | CC(C)(C)O |
|  | Cc1ncco1 |  |
| benzothiazole-4-Me |  | t-butylacetate |
| Cc1cccc(c12)scn2 | oxazole-2-NH2 | CC(=O)OC(C)(C)C |
|  | Nc1ncco1 |  |
| benzothiazole-6-1-EtNH2_S_Prot |  | t-butylamine_Prot |
| CC([NH3+])c(cc1)cc(c12)scn2 | oxazolidin-2-one | CC(C)(C)[NH3+] |
|  | O=C1NCCO1 |  |
| benzothiazole-24-diMe |  | t-butylether |
| Cc(n1)sc(c12)cccc2C | oxazolidin-2-one--N-26-diFPh_A | CC(C)(C)OC |
|  | O1CCN(C1=O)c2c(F)cccc2F |  |
| benzothiophene |  | tetrahydrofuran |
| c1csc(c12)cccc2 | oxazolidin-2-one-3-Me | C1CCOC1 |
|  | O=C1N(C)CCO1 |  |
| benzothiophene-3-Me |  | tetrahydrofuran_B |
| Cc1csc(c12)cccc2 | oxazolidin-2-one-3-Me-4-NH2-Prot_S | C1CCOC1 |
|  | O=C1N(C)C(CO1)[NH3+] |  |
| benzothiophene-5-Me |  |  |
| Cc(c1)ccc(c12)scc2 | oxetane | tetrahydropyran |
|  | C1COC1 | C1CCOCC1 |
| benzoxazine |  |  |
| N1=CCOc(c12)cccc2 | oxime_3P9H | tetrahydropyran-3-CN-4-OH-3S4S |
|  | C=NOC | N#CC1C(O)CCOC1 |
| benzoxazine-dihydro-NMe |  |  |
| CN1CCOc(c12)cccc2 | phenol | tetrahydropyran-4-Me |
|  | Oc1ccccc1 | CC1CCOCC1 |
| benzoxazole |  |  |
| n1coc(c12)cccc2 | phenol-2-CF3 | tetrahydropyrimidin-2-one |
|  | FC(F)(F)c1c(O)cccc1 | O=C1NCCCN1 |
| betalactam |  |  |
| O=C1CCN1 | phenol-2-Cl | tetrahydropyrimidin-2-one-1-Me_A |
|  | Oc1c(Cl)cccc1 | O=C1N(C)CCCN1 |
| bicyclo-221-octane-1-NH2-4-Me_Prot |  |  |
| CC12CCC([NH3+])(CC1)CC2 | phenol-2-isopropyl | tetrahydropyrimidin-2-one-1-Me_B |
|  | CC(C)c1c(O)cccc1 | O=C1N(C)CCCN1 |
| bicyclo-222-octane-2-OH-2-CN-33-diMe |  |  |
| CC(C)(C12)C(C#N)(O)C(CC1)CC2 | phenol-2-Me | tetrahydroquinolin-2-one-NEt-5-oximeOMe |
|  | Cc1c(O)cccc1 | c1cc(=O)n(CC)c(c12)CCCC\2=N\OC |
| bicyclo222octane-1-NH2_Prot |  |  |
| [NH3+]C12CCC(CC1)CC2 | phenol-3-Cl_anti | tetrahydroquinolin-2-one-NMe-5-oximeOMe-3-COO_minus |
|  | Oc1cc(Cl)ccc1 | O=C(O)c(c1)c(=O)n(C)c(c12)CCCC\2=N\OC |
| bipheny-3-F |  |  |
| c1ccc(F)cc1-c2ccccc2 | phenol-3-isopropanol | tetrahydrothiophene-1-oxide |
|  | CC(C)(O)c1cc(O)ccc1 | C1CCCS1=O |
| biphenyl |  |  |
| c1ccccc1-c2ccccc2 |  | tetramic_acid_N_Me |
|  | phenol-3-isopropyl | CN(C1)C(=O)C=C1O |
| biphenyl-2-CN_A | CC(C)c1cc(O)ccc1 |  |
| c1cccc(c1C#N)-c2ccccc2 |  | tetrazole-5-Me |
|  | phenol-3-Me | Cc1nnn[nH]1 |
| biphenyl_B | Cc1cc(O)ccc1 |  |
| c1ccccc1-c2ccccc2 |  | tetrazole-5-NH2-1-Me |
|  | phenol-3-SO2Me | Cn1nnnc1N |
| but-2-ene-2-Me | Oc1cc(S(=O)(=O)C)ccc1 |  |
| CC(C)=CC |  | tetrazole-NMe |
|  | phenol-4-Me | Cn1cnnn1 |
| but-2-ene_cis | Cc1ccc(O)cc1 |  |
| C/C=C\C |  | thiadiazine-dioxide-2-Me |
|  | phenol-24-diMe | CN1C=CC=NS1(=O)=O |
| but-2-ene_trans | Cc1c(O)ccc(C)c1 |  |
| C/C=C/C |  | thiadiazine-dioxide-3-NH2-2-Me |
|  | phenol-26-diMe | CN1C(N)=CC=NS1(=O)=O |
| but-2-yne | Cc1c(O)c(C)ccc1 |  |
| CC#CC |  | thiadiazine-dioxide-5-NH2-2-Me |
|  | phenol-35-diMe | CN1C=CC(N)=NS1(=O)=O |
| butane-2-Me_glb_A | Cc1cc(C)cc(c1)O |  |
| CC(C)CC |  | thiadiazine-dioxide-35-diNH2-2-Me |
|  | phthalazine | CN1C(N)=CC(N)=NS1(=O)=O |
| butane-2-Me_glb_B | c1nncc(c12)cccc2 |  |
| CC(C)CC |  | thiadiazole |
|  | phthalimide-N-Me | c1csnn1 |
| butane-2-Me_high | O=C1N(C)C(=O)c(c12)cccc2 |  |
| CC(C)CC |  | thiadiazole-4-Me |
|  | pinane | Cc1csnn1 |
|  | CC(C)(C12)C(C1)CCC2C |  |
| butane-2233-tetraMe |  | thiatriazine-dioxide-3-NH2-2-Me |
| CC(C)(C)C(C)(C)C | piperazin-2-one | CN1C(N)=NC=NS1(=O)=O |
|  | O=C1CNCCN1 |  |
| butane_glb |  | thiazole |
| CCCC | piperazin-2-one-1-Me | c1cscn1 |
|  | CN1CCNCC1=O |  |
| butane_med_A |  | thiazole-2-CN |
| CCCC | piperazin-2-one-1-Me-4-Et_A | N#Cc1nccs1 |
|  | O=C1CN(CC)CCN1C |  |
| butane_med_B |  | thiazole-2-Et_A |
| CCCC | piperazin-2-one-1-Me-4-Et_B | CCc1nccs1 |
|  | O=C1CN(CC)CCN1C |  |
| caprolactam-N-Me |  | thiazole-2-Et_B |
| CN1CCCCCC1=O | piperazin-2-one-1-Me-4-Et_C | CCc1nccs1 |
|  | O=C1CN(CC)CCN1C |  |
| carbazole-9-Me |  | thiazole-2-iPr_glb |
| c1cccc(c1c23)n(C)c2cccc3 | piperazin-2-one-1-Me-4-Et_D | CC(C)c1nccs1 |
|  | O=C1CN(CC)CCN1C |  |
| catechol |  | thiazole-2-iPr_med |
| Oc1c(O)cccc1 | piperazin-2-one-1-Me_Prot_A | CC(C)c1nccs1 |
|  | CN1CC[NH2+]CC1=O |  |
| chroman-22-diMe |  | thiazole-2-Me |
| C1CC(C)(C)Oc(c12)cccc2 | piperazin-2-one-1-Me_Prot_B | Cc1nccs1 |
|  | CN1CC[NH2+]CC1=O |  |
| cinnoline |  | thiazole-2-NH2 |
| c1cnnc(c12)cccc2 | piperazin-2-one-4-Et_A | Nc1nccs1 |
|  | O=C1CN(CC)CCN1 |  |
| cinnoline-5-CN-3-Et |  | thiazole-2-NH2-4-Me |
| N#Cc1cccc(c12)nnc(c2)CC | piperazin-2-one-4-Et_B | Cc1csc(n1)N |
|  | O=C1CN(CC)CCN1 |  |
| cresol-m |  | thiazole-2-NH2-5-Me |
| Cc1cc(O)ccc1 | piperazin-2-one-4-Et_C | Cc1cnc(s1)N |
|  | O=C1CN(CC)CCN1 |  |
| cresol-o |  | thiazole-2-NHMe_glb |
| Cc1c(O)cccc1 | piperazin-2-one-4-Et_D | CNc1nccs1 |
|  | O=C1CN(CC)CCN1 |  |
| cresol-p |  | thiazole-2-NHMe_high |
| Cc1ccc(O)cc1 | piperazin-2-one-4-Me | CNc1nccs1 |
|  | O=C1CN(C)CCN1 |  |
| cyclobutane |  | thiazole-2-NMe2 |
| C1CCC1 | piperazin-2-one-4-Me_B | CN(C)c1nccs1 |
|  | O=C1CN(C)CCN1 |  |
| cyclohexan-1-OH-1-Ph_A |  |  |
| c1ccccc1C2(O)CCCCC2 | piperazin-2-one-14-diMe | thiazole-2-OMe_glb |
|  | CN1CCN(C)CC1=O | COc1nccs1 |
| cyclohexane |  |  |
| C1CCCCC1 | piperazin-2-one-N-Ms_Prot_A | thiazole-2-OMe_high |
|  | CS(=O)(=O)N1CC[NH2+]CC1=O | COc1nccs1 |
| cyclohexane-1-CN-1-Me |  |  |
| N#CC1(C)CCCCC1 | piperazin-2-one-N-Ms_Prot_B | thiazole-2-SMe-4-Me |
|  | CS(=O)(=O)N1CC[NH2+]CC1=O | Cc1csc(n1)SC |
| cyclohexane-1-CN-1-Ph_A |  |  |
| C1CCCCC1(C#N)c2ccccc2 | piperazin-2-one_Prot_A | thiazole-24-diMe |
|  | O=C1C[NH2+]CCN1 | Cc1csc(n1)C |
| cyclohexane-1-CN-1-Ph_B |  |  |
| C1CCCCC1(C#N)c2ccccc2 | piperazin-2-one_Prot_B | thiazolium-N-Me |
|  | O=C1C[NH2+]CCN1 | CN1C=CSC1 |
| cyclohexane-1-CN-axial |  |  |
| N#CC1CCCCC1 | piperazine | thietane |
|  | C1CNCCN1 | C1CSC1 |
| cyclohexane-1-OH_A |  |  |
| OC1CCCCC1 | piperazine-4-1-Me-pyrazol-4-yl-sulfonyl_Prot_A | thioanisole |
|  | Cn(c1)ncc1S(=O)(=O)N2CC[NH2+]CC2 | CSc1ccccc1 |
| cyclohexane-1-OH_axial_A |  |  |
| OC1CCCCC1 | piperazine-4-1-Me-pyrazol-4-yl-sulfonyl_Prot_B | thioether |
|  | Cn(c1)ncc1S(=O)(=O)N2CC[NH2+]CC2 | CSC |
| cyclohexane-1-OH_B |  |  |
| OC1CCCCC1 | piperazine-4-Ac_Prot_A | thiohydantoin-3-Me |
|  | CC(=O)N1CC[NH2+]CC1 | CN1C(=S)NCC1=O |
| cyclohexane-1-OH_C |  |  |
| OC1CCCCC1 |  | thiol |
|  | piperazine-4-Ac_Prot_B | CS |
| cyclohexane-14-diMe | CC(=O)N1CC[NH2+]CC1 |  |
| CC1CCC(C)CC1 |  | thiomorpholin-3-one-N-Me |
|  | piperazine-4-Me-1-amidino-N-Me_anti_Prot | CN1CCSCC1=O |
| cyclohexane-CN | CNC(=[NH2+])N1CCN(C)CC1 |  |
| N#CC1CCCCC1 |  | thiomorpholine |
|  | piperazine-4-Me-1-amidino-N-Me_syn_Prot | C1CSCCN1 |
| cyclohexane-Me | CNC(=[NH2+])N1CCN(C)CC1 |  |
| CC1CCCCC1 |  | thiomorpholine-N-Me-11-dioxide |
|  | piperazine-4-Me-1-amidino-N1-Me-N2-Me_anti_Prot | C1CN(C)CCS1(=O)=O |
| cyclohexanone | CN/C(=[NH+]\C)N1CCN(C)CC1 |  |
| O=C1CCCCC1 |  | thiophene |
|  | piperazine-4-Me-1-amidino-N1-Me-N2-Me_syn_Prot | c1ccsc1 |
| cyclohexylamine-spiroC6_Prot | CN/C(=[NH+]\C)N1CCN(C)CC1 |  |
| C1CCCCC12CCC([NH3+])CC2 |  | thiophene-2-Cl |
|  | piperazine-4-Me-1-amidino_Prot | Clc1cccs1 |
| cyclohexylamine_Prot | NC(=[NH2+])N1CCN(C)CC1 |  |
| [NH3+]C1CCCCC1 |  | thiophene-2-CN |
|  | piperazine-4-Me-1-carboximidamide_Prot | N#Cc1cccs1 |
| cyclooctylamine_Prot_A | NC(=[NH2+])N1CCN(C)CC1 |  |
| [NH3+]C1CCCCCCC1 |  | thiophene-2-CONH2 |
|  | piperazine-4-Ms_Prot_A | NC(=O)c1cccs1 |
| cyclooctylamine_Prot_B | CS(=O)(=O)N1CC[NH2+]CC1 |  |
| [NH3+]C1CCCCCCC1 |  | thiophene-2-Et_glb_A |
|  | piperazine-4-Ms_Prot_B | CCc1cccs1 |
| cyclopentane | CS(=O)(=O)N1CC[NH2+]CC1 |  |
| C1CCCC1 |  | thiophene-2-Et_glb_B |
|  | piperazine-4-pyrrol-1yl-sulfonyl_Prot | CCc1cccs1 |
|  | c1cccn1S(=O)(=O)N2CC[NH2+]CC2 |  |
| cyclopentene |  | thiophene-2-Et_glb_C |
| C1=CCCC1 | piperazine-4-SO2NH2_Prot_A | CCc1cccs1 |
|  | NS(=O)(=O)N1CC[NH2+]CC1 |  |
| cyclopropane |  | thiophene-2-iPr_glb |
| C1CC1 | piperazine-4-SO2NH2_Prot_B | CC(C)c1cccs1 |
|  | NS(=O)(=O)N1CC[NH2+]CC1 |  |
| cyclopropane-Me |  | thiophene-2-iPr_med |
| CC1CC1 | piperazine-N-Me | CC(C)c1cccs1 |
|  | CN1CCNCC1 |  |
| decahydroisoquinoline-6-Me_Prot |  | thiophene-2-Me |
| CC(C1)CCC(C12)C[NH2+]CC2 | piperazine-N-Me-N-Ac | Cc1cccs1 |
|  | CC(=O)N1CCN(C)CC1 |  |
| decahydroisoquinoline_Prot_A |  | thiophene-2-NHMe_A |
| C1C[NH2+]CC(C12)CCCC2 | piperazine-N-Me_Prot | CNc1cccs1 |
|  | CN1CC[NH2+]CC1 |  |
| diazepan-4-Ac_Prot_A |  | thiophene-2-NHMe_B |
| CC(=O)N1CCC[NH2+]CC1 | piperazine-NN-diAc | CNc1cccs1 |
|  | CC(=O)N1CCN(C(=O)C)CC1 |  |
| diazepan-4-Ac_Prot_B |  | thiophene-2-NMe2 |
| CC(=O)N1CCC[NH2+]CC1 | piperazine-NN-diMe | CN(C)c1cccs1 |
|  | CN1CCN(C)CC1 |  |
| difluoroethane |  | thiophene-2-OH_A |
| FC(F)C | piperazine-NN-diMe_Prot | Oc1cccs1 |
|  | CN1CC[NH+](C)CC1 |  |
| difluoropropane-22 |  | thiophene-2-OH_B |
| CC(F)(F)C | piperidin-2-one-N-Me | Oc1cccs1 |
|  | CN1CCCCC1=O |  |
| dihydro-16-pyridin-6-one-NMe-3-NH2-Prot |  | thiophene-2-OMe_glb |
| Cn(c1)c(=O)ccc1[NH3+] | piperidin-2-one_A | COc1cccs1 |
|  | O=C1CCCCN1 |  |
| dihydro-23-imidazol-2-one-1diMe-4-NH2_Prot |  | thiophene-2-OMe_high |
| Cn1c(=O)n(C)cc1[NH3+] | piperidin-2-one_B | COc1cccs1 |
|  | O=C1CCCCN1 |  |
| dihydroisoquinoline-18-dione-3-NH2_A |  | thiophene-3-Me |
| O=c1[nH]c(N)cc(c12)CCCC2=O | piperidine | Cc1ccsc1 |
|  | C1CCNCC1 |  |
| dihydroisoquinoline-18-dione-3-NH2_B |  | thiophene-3-NHAc |
| O=c1[nH]c(N)cc(c12)CCCC2=O | piperidine-4-CN-N-Ac_A | CC(=O)Nc1ccsc1 |
|  | N#CC1CCN(C(=O)C)CC1 |  |
| dihydrooxazole |  | thiophene-3-OH_A |
| C1=NCCO1 | piperidine-4-CN-N-Ac_B | Oc1ccsc1 |
|  | N#CC1CCN(C(=O)C)CC1 |  |
| dihydroquinazoline-45-dione-2-NH2-1-Me_A |  |  |
| OC1N=C(N)N(C)C(C=12)CCCC2=O | piperidine-4-morpholino_Prot | thiophene-3-OH_B |
|  | C1C[NH2+]CCC1N2CCOCC2 | Oc1ccsc1 |
| dihydroquinazoline-45-dione-2-NH2-1-Me_B |  |  |
| OC1N=C(N)N(C)C(=C12)CCCC2=O | piperidine-4-OH-4-Me_Prot | thiophene-11-dioxide |
|  | CC1(O)CC[NH2+]CC1 | C1=CC=CS1(=O)=O |
| dihydroquinazoline-45-dione-2-NH2_A |  |  |
| O=c1[nH]c(N)nc(c12)CCCC2=O | piperidine-4-OH-4-Ph_Prot_A | thiopyran |
|  | c1ccccc1C2(O)CC[NH2+]CC2 | C1CCSCC1 |
| dihydroquinazoline-45-dione-2-NH2_B |  |  |
| O=c1[nH]c(N)nc(c12)CCCC2=O | piperidine-4-OH-4-Ph_Prot_B | thiopyran-1-oxide |
|  | c1ccccc1C2(O)CC[NH2+]CC2 | C1CCCCS1=O |
| dihydroquinoline-45-dione-2-NH2-1-Me_A |  |  |
| OC1C=C(N)N(C)C(=C12)CCCC2=O | piperidine-4-piperidin-4-yl-N-Ac_180_Prot_A | threo |
|  | CC(=O)N(CC1)CCC1C2CC[NH2+]CC2 | OC1C(O)COC1O |
| dihydroquinoline-45-dione-2-NH2-1-Me_B |  |  |
| OC1C=C(N)N(C)C(=C12)CCCC2=O | piperidine-4-piperidin-4-yl-N-Ac_180_Prot_B | thymine |
|  | CC(=O)N(CC1)CCC1C2CC[NH2+]CC2 | Cc1cc(=O)[nH]c(=O)[nH]1 |
| dimethylamine |  |  |
| CNC | piperidine-4-piperidin-4-yl-N-Ac_minus60_Prot_A | toluene |
|  | CC(=O)N(CC1)CCC1C2CC[NH2+]CC2 | Cc1ccccc1 |
| dimethylamine_Prot |  |  |
| C[NH2+]C | piperidine-4-piperidin-4-yl-N-Ac_minus60_Prot_B | triaza-134-1H-indene-7-CCMe |
|  | CC(=O)N(CC1)CCC1C2CC[NH2+]CC2 | CC#Cc1ccnc(c12)nc[nH]2 |
| dimethylsulfone |  |  |
| CS(=O)(=O)C | piperidine-4-piperidin-4-yl-N-Ac_plus60_Prot_A | triazine-24-diNH2-1-OMe-66-diMe_Prot_A |
|  | CC(=O)N(CC1)CCC1C2CC[NH2+]CC2 | CC1(C)N(OC)C(N)=NC(N)=[NH+]1 |
| dimethylsulfoxide |  |  |
| CS(=O)C |  | triazine-135 |
|  | piperidine-4-piperidin-4-yl-N-Ac_plus60_Prot_B | c1ncncn1 |
| dioxane-13 | CC(=O)N(CC1)CCC1C2CC[NH2+]CC2 |  |
| C1COCOC1 |  | triazole-123-1-Me |
|  | piperidine-4-spiroC5_Prot | Cn1ccnn1 |
| dioxane-14 | C1CCCC12CC[NH2+]CC2 |  |
| C1COCCO1 |  | triazole-123-2H |
|  | piperidine-4-spiroC6_Prot | c1cn[nH]n1 |
| erythro | C1C[NH2+]CCC12CCCCC2 |  |
| OC1C(O)COC1O |  | triazole-123-5-Me_Prot |
|  | piperidine-N-2-pyrimidinyl-4-NH2_Prot | CC1=CNNN1 |
| ethane | n1cccnc1N(CC2)CCC2[NH3+] |  |
| CC |  | triazole-124 |
|  | piperidine-N-Ac-4-NH2_Prot_A | c1nnc[nH]1 |
| ethane-1-CN | CC(=O)N(CC1)CCC1[NH3+] |  |
| N#CCC |  | triazole-124-3-Me-4H |
|  | piperidine-N-Ac-4-NH2_Prot_B | Cc1nnc[nH]1 |
| ethane-OEt | CC(=O)N(CC1)CCC1[NH3+] |  |
| CCOCC |  | triazole-124-3-NH2_4H |
|  | piperidine-N-Ac-33-diMe_A | Nc1nnc[nH]1 |
| ethane-OMe | CC(=O)N(C1)CCCC1(C)C |  |
| CCOC |  | triazole-124-3-NHMe-4-Me_Anti |
|  | piperidine-N-Ac-33-diMe_B | CNc1nncn1C |
| ethanol | CC(=O)N(C1)CCCC1(C)C |  |
| OCC |  | triazole-124-3-NHMe-4-Me_Syn |
|  | piperidine-N-Me | CNc1nncn1C |
| ethanol_60deg_A | CN1CCCCC1 |  |
| OCC |  | triazole-124-34-diMe |
|  | piperidine-N-Me-4-NH2_Prot | Cc1nncn1C |
|  | C1C[NH+](C)CCC1N |  |
| ethanol_60deg_B |  | triazole-124-NMe |
| OCC | piperidine-N-Me-4-OH-4-Me_Prot | Cn1cnnc1 |
|  | CC1(O)CC[NH+](C)CC1 |  |
| ether |  | triazole-124_1H |
| COC | piperidine_Prot | c1nc[nH]n1 |
|  | C1CC[NH2+]CC1 |  |
| ethyl-Cl |  | triazole-124_4H |
| ClCC | piperidinium-N-diMe | c1nnc[nH]1 |
|  | C[N+]1(C)CCCCC1 |  |
| ethylamine |  | triazolo-43a-pyrimidine-57-diMe |
| NCC | piperidinyl-2-pyrimidyl-4-NH2_Prot | Cc(n1)cc(C)n(c12)cnn2 |
|  | n1cccnc1N(CC2)CCC2[NH3+] |  |
| ethylamine_Prot |  | triazolo-124-43a-tetrahydropyrazine-3-Me_Prot_A |
| [NH3+]CC | propamide | n1nc(C)n(c12)CC[NH2+]C2 |
|  | NC(=O)CC |  |
| ethylene |  | triazolo-124-43a-tetrahydropyrazine-3-Me_Prot_B |
| C=C | propamide-N-diMe-2-Me_glb_A | n1nc(C)n(c12)CC[NH2+]C2 |
|  | CN(C)C(=O)C(C)C |  |
| ethylene-11-diF |  | trifluoroacetone |
| FC(F)=C | propamide-N-diMe-2-Me_glb_B | CC(=O)C(F)(F)F |
|  | CN(C)C(=O)C(C)C |  |
| fluoroethane |  | trifluoroethane |
| FCC | propamide-N-diMe_glb | FC(F)(F)C |
|  | CN(C)C(=O)CC |  |
| formaldehyde |  | trimethylamine |
| C=O | propamide-N-Me-2-Me_glb_A | CN(C)C |
|  | CNC(=O)C(C)C |  |
| formamide |  | trimethylamine_oxide |
| O=CN | propamide-N-Me-2-Me_glb_B | C[N+]([O-])(C)C |
|  | CNC(=O)C(C)C |  |
| formimidamide-N-carbamimide_B |  | trimethylamine_Prot |
| [H]\N=C(N)\N/C=N\[H] | propamide-N-Me-2-Me_glb_C | C[NH+](C)C |
|  | CNC(=O)C(C)C |  |
| formimidamide-N-carbamimide_Prot_A |  | trimethylphosphineoxide |
| NC(=[NH2+])NC=N | propamide-N-Me_glb | CP(=O)(C)C |
|  | CNC(=O)CC |  |
| formimidamide-N-carbamimide_Prot_B |  | tropolone-3-Me |
| NC(=[NH2+])NC=N | propamide-N-Me_med_A | Cc1c(O)c(=O)cccc1 |
|  | CNC(=O)CC |  |
| formimidamide-N-carbamimide_syn |  | uracil-N-Me |
| [H]\N=C(N)\NC=N | propamide-N-Me_med_B | [nH]1c(=O)n(C)ccc1=O |
|  | CNC(=O)CC |  |
| formimidamide-N-carbamimide_syn_B |  |  |
| [H]\N=C(N)\N/C=N\[H] | propane | urea |
|  | CCC | NC(=O)N |
| formimidamide-N-carbamimide_syn_Prot |  |  |
| NC(=[NH2+])NC=N | propane-2-OMe_A | urea-N-Me |
|  | CC(C)OC | NC(=O)NC |
| furan |  |  |
| c1ccoc1 | propane-2-OMe_B | urea-N-Me-N'-Ph |
|  | CC(C)OC | CNC(=O)Nc1ccccc1 |
| furan-2-Me |  |  |
| Cc1ccco1 | propene | urea-N-Me-N-3-pyridyl-6-CF3 |
|  | CC=C | CNC(=O)Nc(cn1)ccc1C(F)(F)F |
| furan-2-one-3-OH-4-OMe_B |  |  |
| COC(=C1O)COC1=O | propene-1-Cl_Z | urea-N-Me-N-5-pyrimidinyl-2-CF3 |
|  | C/C=C\Cl | CNC(=O)Nc(cn1)cnc1C(F)(F)F |
| furan-2-one-4-Me |  |  |
| CC1=CC(=O)OC1 | propene-1-CN_Z | urea-N-Me_syn |
|  | N#C/C=C\C | NC(=O)NC |
| furan-3-Me |  |  |
| Cc1ccoc1 | propene-2-Me | urea-NN'-diMe |
|  | CC(=C)C | CNC(=O)NC |
| furanone |  |  |
| O=C1C=CCO1 | propionitrile-2-N-Me_glb_A | urea-NN'-diPh |
|  | N#CC(C)NC | c1ccccc1NC(=O)Nc2ccccc2 |
| furanone-dihydro-2-NH2_Prot_A |  |  |
| O=C1C([NH3+])CCO1 | propionitrile-2-N-Me_glb_B | vinyl_Cl |
|  | N#CC(C)NC | ClC=C |
| furanone-dihydro-2-NH2_Prot_B |  |  |
| O=C1C([NH3+])CCO1 |  | water |
|  | propionitrile-2-N-Me_med_A | [O] |
| furanone-dihydro-3-NH2-3-CCH_Prot_A | N#CC(C)NC |  |
| C#CC1([NH3+])C(=O)OCC1 |  | xylene_meta |
|  | propionitrile-2-N-Me_med_B | Cc1cc(C)ccc1 |
| furanone-dihydro-3-NH2_Prot_A | N#CC(C)NC |  |
| O=C1C([NH3+])CCO1 |  | xylene_ortho |
|  | propionitrile-2-NAc-2-Me_glb_A | Cc1c(C)cccc1 |
| furanone-dihydro-3-NH2_Prot_B | N#CC(C)(C)NC(=O)C |  |
| O=C1C([NH3+])CCO1 |  | xylene_para |
|  |  | Cc1ccc(C)cc1 |
