# Supplementary material for: Fragment-based design of small molecule PCSK9 inhibitors using simulated annealing of chemical potential simulations
Source: PLoS One. 2019 Dec 5;14(12):e0225780. doi: 10.1371/journal.pone.0225780 (PMC6894869; doi:10.1371/journal.pone.0225780)
Supplement: S3 Table — (DOCX) [file pone.0225780.s003.docx]

**Supporting Information**

**Designing Small Molecule PCSK9 Inhibitors Guided by Simulated Annealing of Chemical Potential Simulations**

*Frank Guarnieri^1,2^, John L. Kulp Jr.^3^, John L. Kulp III^3,4^, Ian S. Cloudsdale^3^

^1^Center for Drug Discovery, Northeastern University, Boston, MA 02115 USA

^2^PAKA Pulmonary Pharmaceuticals, Acton, MA 01720 USA

^3^Conifer Point Pharmaceuticals, Doylestown, PA 18902 USA

^4^Department of Chemistry, Baruch S. Blumberg Institute, Doylestown, PA 18902 USA

*Corresponding author

Email: [frankguarnieri@yahoo.com](mailto:frankguarnieri@yahoo.com)

**Contents**

1. S1 Table. List of standard AMBER charges and custom derived charges for PCSK9-LDLR
2. S2 Table. List of fragments run on PCSK9
3. S3 Table. List of standard AMBER charges and custom charges for the CN-benzimidazole fragment bound to PCSK9
4. S1 Fig. Ball-and-stick representation of the connected path of interpenetrating atoms.
5. S2 Fig. Examples of π-π stacking.
6. S3 Fig. GAMESS input parameters
7. S4 Fig. Synthetic schemes for fragments and compounds

**S3 Table.** List of standard AMBER charges and custom charges for the CN-benzimidazole fragment bound to PCSK9.

|  | **PCSK9 Chain A** | **Amino Acid Dielectric 1** | **Amino Acid Amber Charge** |
| --- | --- | --- | --- |
| Res | 3GCW_ALA220_A | ALA-ref | ALA-Amber |
| Totq | -0.074 | 0.000 | 0.000 |
| BBq | -0.280 | -0.222 | -0.114 |
| N | -0.663 | -0.539 | -0.416 |
| CA | 0.083 | 0.124 | 0.034 |
| C | 0.500 | 0.593 | 0.597 |
| O | -0.536 | -0.569 | -0.568 |
| CB | -0.315 | -0.061 | -0.182 |
| HA | 0.104 | 0.116 | 0.082 |
| HB1 | 0.112 | 0.014 | 0.060 |
| HB2 | 0.112 | 0.014 | 0.060 |
| HB3 | 0.112 | 0.014 | 0.060 |
| H | 0.418 | 0.293 | 0.272 |
|  |  |  |  |
| Res | 3GCW_ARG215_A | ARG-ref | ARG-Amber |
| Totq | 1.036 | 1.000 | 1.000 |
| BBq | -0.084 | 0.017 | 0.072 |
| N | -0.331 | -0.359 | -0.348 |
| CA | 0.159 | 0.119 | -0.264 |
| C | 0.347 | 0.652 | 0.734 |
| O | -0.365 | -0.535 | -0.589 |
| CB | -0.244 | -0.230 | -0.001 |
| CG | 0.070 | -0.252 | 0.039 |
| CD | -0.085 | 0.514 | 0.049 |
| NE | -0.467 | -0.741 | -0.529 |
| CZ | 0.739 | 0.749 | 0.808 |
| NH1 | -0.820 | -0.876 | -0.863 |
| NH2 | -0.820 | -0.876 | -0.863 |
| H | 0.265 | 0.259 | 0.275 |
| HA | -0.004 | -0.052 | 0.156 |
| HB2 | 0.108 | 0.104 | 0.033 |
| HB3 | 0.108 | 0.104 | 0.033 |
| HG2 | 0.035 | 0.112 | 0.029 |
| HG3 | 0.035 | 0.112 | 0.029 |
| HD2 | 0.099 | 0.006 | 0.069 |
| HD3 | 0.099 | 0.006 | 0.069 |
| HE | 0.349 | 0.385 | 0.346 |
| HH11 | 0.439 | 0.450 | 0.448 |
| HH12 | 0.439 | 0.450 | 0.448 |
| HH21 | 0.439 | 0.450 | 0.448 |
| HH22 | 0.439 | 0.450 | 0.448 |
|  |  |  |  |
| Res | 3GCW_ARG218_A | ARG-ref | ARG-Amber |
| Totq | 0.787 | 1.000 | 1.000 |
| BBq | -0.193 | 0.017 | 0.072 |
| N | -0.345 | -0.359 | -0.348 |
| CA | -0.101 | 0.119 | -0.264 |
| C | 0.318 | 0.652 | 0.734 |
| O | -0.433 | -0.535 | -0.589 |
| CB | 0.097 | -0.230 | -0.001 |
| CG | 0.067 | -0.252 | 0.039 |
| CD | -0.319 | 0.514 | 0.049 |
| NE | -0.122 | -0.741 | -0.529 |
| CZ | 0.402 | 0.749 | 0.808 |
| NH1 | -0.674 | -0.876 | -0.863 |
| NH2 | -0.674 | -0.876 | -0.863 |
| H | 0.268 | 0.259 | 0.275 |
| HA | 0.138 | -0.052 | 0.156 |
| HB2 | 0.023 | 0.104 | 0.033 |
| HB3 | 0.023 | 0.104 | 0.033 |
| HG2 | 0.033 | 0.112 | 0.029 |
| HG3 | 0.033 | 0.112 | 0.029 |
| HD2 | 0.091 | 0.006 | 0.069 |
| HD3 | 0.091 | 0.006 | 0.069 |
| HE | 0.308 | 0.385 | 0.346 |
| HH11 | 0.391 | 0.450 | 0.448 |
| HH12 | 0.391 | 0.450 | 0.448 |
| HH21 | 0.391 | 0.450 | 0.448 |
| HH22 | 0.391 | 0.450 | 0.448 |
|  |  |  |  |
| Res | 3GCW_ASP374_A | ASP-ref | ASP-Amber |
| Totq | -0.606 | -1.000 | -1.000 |
| BBq | 0.066 | -0.309 | -0.268 |
| N | 0.009 | -0.461 | -0.516 |
| CA | 0.020 | 0.029 | 0.038 |
| C | 0.472 | 0.453 | 0.537 |
| O | -0.516 | -0.509 | -0.582 |
| CB | -0.093 | -0.116 | -0.030 |
| CG | 0.705 | 0.743 | 0.799 |
| OD1 | -0.706 | -0.706 | -0.801 |
| OD2 | -0.706 | -0.706 | -0.801 |
| H | 0.100 | 0.207 | 0.294 |
| HA | 0.080 | 0.081 | 0.088 |
| HB2 | 0.014 | -0.008 | -0.012 |
| HB3 | 0.014 | -0.008 | -0.012 |
|  |  |  |  |
| Res | 3GCW_GLN219_A | GLN-ref | GLN-Amber |
| Totq | 0.192 | 0.000 | 0.000 |
| BBq | -0.025 | -0.252 | -0.114 |
| N | -0.047 | -0.616 | -0.416 |
| CA | 0.015 | 0.169 | -0.003 |
| C | 0.244 | 0.553 | 0.597 |
| O | -0.286 | -0.507 | -0.568 |
| CB | -0.113 | 0.119 | -0.004 |
| CG | -0.042 | -0.423 | -0.065 |
| CD | 0.625 | 0.611 | 0.695 |
| OE1 | -0.524 | -0.543 | -0.609 |
| NE2 | -0.864 | -0.796 | -0.941 |
| H | 0.064 | 0.318 | 0.272 |
| HA | 0.157 | 0.093 | 0.085 |
| HB2 | 0.027 | 0.007 | 0.017 |
| HB3 | 0.027 | 0.007 | 0.017 |
| HG2 | 0.042 | 0.091 | 0.035 |
| HG3 | 0.042 | 0.091 | 0.035 |
| HE21 | 0.412 | 0.414 | 0.425 |
| HE22 | 0.412 | 0.414 | 0.425 |
|  |  |  |  |
| Res | 3GCW_LYS222_A | LYS-ref | LYS-Amber |
| Totq | 0.873 | 1.000 | 1.000 |
| BBq | 0.127 | 0.098 | 0.072 |
| N | -0.130 | -0.375 | -0.348 |
| CA | -0.330 | -0.286 | -0.240 |
| C | 0.513 | 0.655 | 0.734 |
| O | -0.463 | -0.525 | -0.589 |
| CB | 0.000 | -0.082 | -0.009 |
| CG | 0.043 | 0.375 | 0.019 |
| CD | -0.097 | 0.143 | -0.048 |
| CE | 0.058 | -0.039 | -0.014 |
| NZ | -0.341 | -0.364 | -0.385 |
| H | 0.207 | 0.343 | 0.275 |
| HA | 0.240 | 0.166 | 0.143 |
| HB2 | 0.030 | 0.044 | 0.036 |
| HB3 | 0.030 | 0.044 | 0.036 |
| HG2 | -0.020 | -0.130 | 0.010 |
| HG3 | -0.020 | -0.130 | 0.010 |
| HD2 | 0.028 | -0.019 | 0.062 |
| HD3 | 0.028 | -0.019 | 0.062 |
| HE2 | 0.070 | 0.106 | 0.114 |
| HE3 | 0.070 | 0.106 | 0.114 |
| HZ1 | 0.319 | 0.329 | 0.340 |
| HZ2 | 0.319 | 0.329 | 0.340 |
| HZ3 | 0.319 | 0.329 | 0.340 |
|  |  |  |  |
| Res | 3GCW_SER221_A | SER-ref | SER-Amber |
| Totq | -0.018 | 0.000 | 0.000 |
| BBq | -0.157 | 0.036 | -0.114 |
| N | -0.259 | -0.429 | -0.416 |
| CA | 0.034 | -0.154 | -0.025 |
| C | 0.299 | 0.614 | 0.597 |
| O | -0.395 | -0.489 | -0.568 |
| CB | -0.012 | 0.285 | 0.212 |
| OG | -0.618 | -0.725 | -0.655 |
| H | 0.198 | 0.340 | 0.272 |
| HA | 0.204 | 0.099 | 0.084 |
| HB2 | 0.044 | 0.002 | 0.035 |
| HB3 | 0.044 | 0.002 | 0.035 |
| HG | 0.442 | 0.453 | 0.427 |
|  |  |  |  |
| Res | 3GCW_SER225_A | SER-ref | SER-Amber |
| Totq | -0.008 | 0.000 | 0.000 |
| BBq | -0.060 | 0.036 | -0.114 |
| N | -0.308 | -0.429 | -0.416 |
| CA | 0.005 | -0.154 | -0.025 |
| C | 0.553 | 0.614 | 0.597 |
| O | -0.495 | -0.489 | -0.568 |
| CB | 0.259 | 0.285 | 0.212 |
| OG | -0.696 | -0.725 | -0.655 |
| H | 0.190 | 0.340 | 0.272 |
| HA | 0.086 | 0.099 | 0.084 |
| HB2 | 0.009 | 0.002 | 0.035 |
| HB3 | 0.009 | 0.002 | 0.035 |
| HG | 0.380 | 0.453 | 0.427 |
|  |  |  |  |
| Res | 3GCW_SER372_A | SER-ref | SER-Amber |
| Totq | -0.048 | 0.000 | 0.000 |
| BBq | -0.206 | 0.036 | -0.114 |
| N | -0.475 | -0.429 | -0.416 |
| CA | 0.064 | -0.154 | -0.025 |
| C | 0.480 | 0.614 | 0.597 |
| O | -0.536 | -0.489 | -0.568 |
| CB | 0.175 | 0.285 | 0.212 |
| OG | -0.615 | -0.725 | -0.655 |
| H | 0.325 | 0.340 | 0.272 |
| HA | 0.082 | 0.099 | 0.084 |
| HB2 | 0.005 | 0.002 | 0.035 |
| HB3 | 0.005 | 0.002 | 0.035 |
| HG | 0.443 | 0.453 | 0.427 |
|  |  |  |  |
| Res | 3GCW_SER373_A | SER-ref | SER-Amber |
| Totq | -0.126 | 0.000 | 0.000 |
| BBq | -0.227 | 0.036 | -0.114 |
| N | -0.311 | -0.429 | -0.416 |
| CA | -0.089 | -0.154 | -0.025 |
| C | 0.262 | 0.614 | 0.597 |
| O | -0.471 | -0.489 | -0.568 |
| CB | 0.086 | 0.285 | 0.212 |
| OG | -0.620 | -0.725 | -0.655 |
| H | 0.293 | 0.340 | 0.272 |
| HA | 0.148 | 0.099 | 0.084 |
| HB2 | 0.065 | 0.002 | 0.035 |
| HB3 | 0.065 | 0.002 | 0.035 |
| HG | 0.446 | 0.453 | 0.427 |
|  |  |  |  |
| Res | 3GCW_VAL380_A | VAL-ref | VAL-Amber |
| Totq | 0.001 | 0.000 | 0.000 |
| BBq | -0.114 | 0.372 | -0.114 |
| N | -0.348 | -0.167 | -0.416 |
| CA | -0.025 | -0.920 | -0.087 |
| C | 0.466 | 0.772 | 0.597 |
| O | -0.469 | -0.512 | -0.568 |
| CB | 0.365 | 0.533 | 0.298 |
| CG1 | -0.376 | -0.489 | -0.319 |
| CG2 | -0.376 | -0.489 | -0.319 |
| H | 0.237 | 0.279 | 0.272 |
| HA | 0.060 | 0.323 | 0.097 |
| HB | -0.058 | 0.013 | -0.030 |
| HG11 | 0.087 | 0.109 | 0.079 |
| HG12 | 0.087 | 0.109 | 0.079 |
| HG13 | 0.087 | 0.109 | 0.079 |
| HG21 | 0.087 | 0.109 | 0.079 |
| HG22 | 0.087 | 0.109 | 0.079 |
| HG23 | 0.087 | 0.109 | 0.079 |
|  |  |  |  |
| Res | 3GCW_Y221_Q | ref | Amber |
| Totq | -0.010 | none | none |
| BBq | 0.000 | none | none |
| C1 | -0.234 | none | none |
| C2 | -0.137 | none | none |
| C3 | -0.188 | none | none |
| C4 | 0.180 | none | none |
| C5 | 0.154 | none | none |
| C6 | -0.096 | none | none |
| H1 | 0.188 | none | none |
| H2 | 0.172 | none | none |
| N1 | -0.080 | none | none |
| H3 | 0.235 | none | none |
| C7 | 0.169 | none | none |
| N2 | -0.553 | none | none |
| H4 | 0.098 | none | none |
| H5 | 0.216 | none | none |
| C8 | 0.445 | none | none |
| N3 | -0.578 | none | none |
