# Supplementary material for: Fragment-based design of small molecule PCSK9 inhibitors using simulated annealing of chemical potential simulations
Source: PLoS One. 2019 Dec 5;14(12):e0225780. doi: 10.1371/journal.pone.0225780 (PMC6894869; doi:10.1371/journal.pone.0225780)

**Supporting Information**

**Designing Small Molecule PCSK9 Inhibitors Guided by Simulated Annealing of Chemical Potential Simulations**

*Frank Guarnieri^1,2^, John L. Kulp Jr.^3^, John L. Kulp III^3,4^, Ian S. Cloudsdale^3^

^1^Center for Drug Discovery, Northeastern University, Boston, MA 02115 USA

^2^PAKA Pulmonary Pharmaceuticals, Acton, MA 01720 USA

^3^Conifer Point Pharmaceuticals, Doylestown, PA 18902 USA

^4^Department of Chemistry, Baruch S. Blumberg Institute, Doylestown, PA 18902 USA

*Corresponding author

Email: [frankguarnieri@yahoo.com](mailto:frankguarnieri@yahoo.com)

**Contents**

1. S1 Table. List of standard AMBER charges and custom derived charges for PCSK9-LDLR
2. S2 Table. List of fragments run on PCSK9
3. S3 Table. List of standard AMBER charges and custom charges for the CN-benzimidazole fragment bound to PCSK9
4. S1 Fig. Ball-and-stick representation of the connected path of interpenetrating atoms.
5. S2 Fig. Examples of π-π stacking.
6. S3 Fig. GAMESS input parameters
7. S4 Fig. Synthetic schemes for fragments and compounds

**S1 Fig.** The connected path of interpenetrating atoms is shown. The complete path cannot be shown in 1 image, because of its complicated 3D curvature and putting all atoms in VDW space fill makes it impossible to see. The image in the upper left shows interpenetrating atoms in small spheres with the green double-headed arrows indicating the partners. The upper left image shows in VWD the interpenetrating atoms across a small disulfide ring. The lower left image shows more of the atoms in VDW space fill with the R194-D310 marked by the double-headed arrow. The upper right image shows part of the connected path from the LDLR Ca^2+^ ion and D310 into the PCSK9 T377 through the disulfide ring, D374 and back to LDLR Y306. The complete list of residues is given in Table S1.


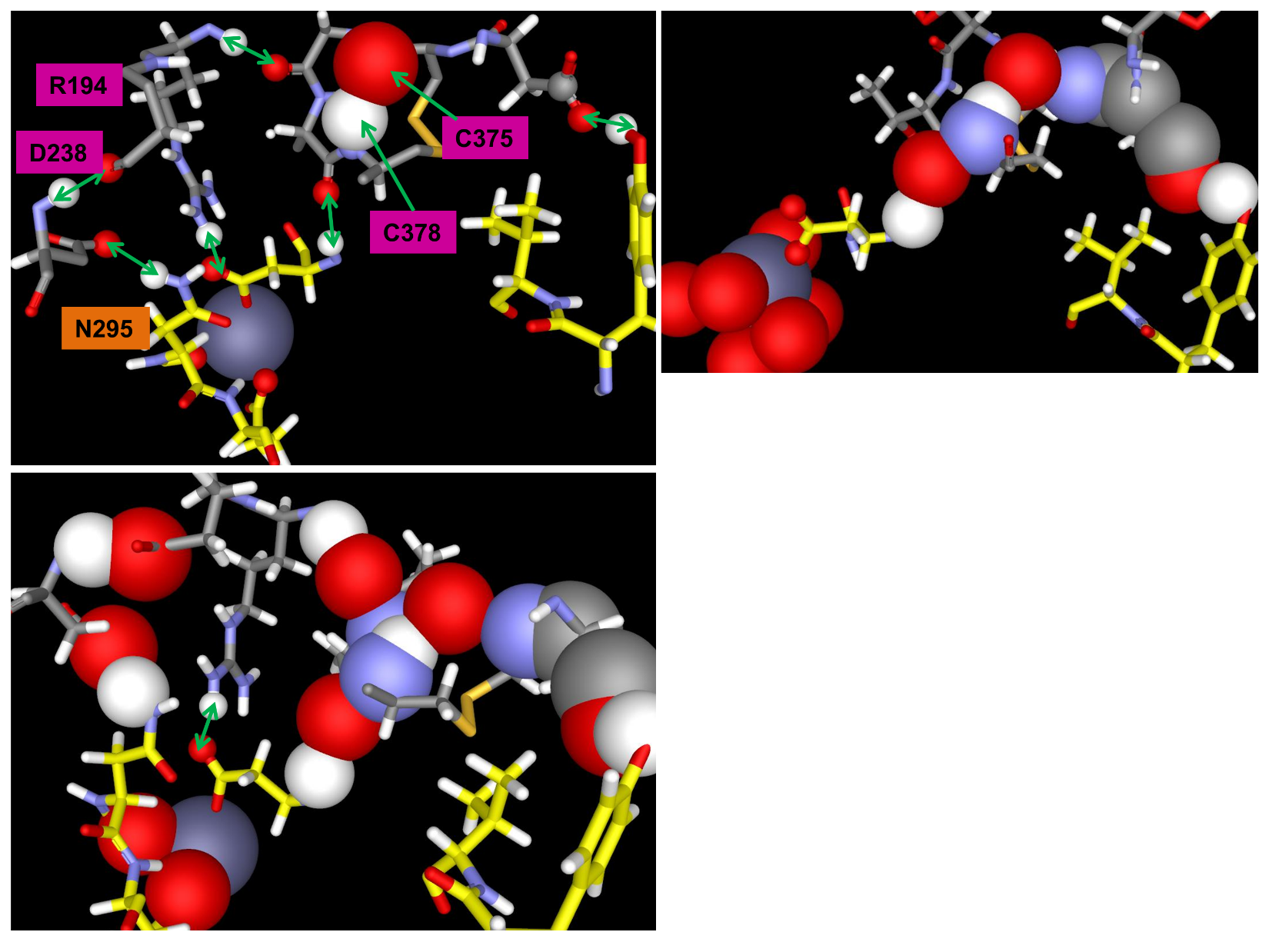

Supplement: S1 Fig — (DOCX) [file pone.0225780.s004.docx]
