# Supplementary material for: Fragment-based design of small molecule PCSK9 inhibitors using simulated annealing of chemical potential simulations
Source: PLoS One. 2019 Dec 5;14(12):e0225780. doi: 10.1371/journal.pone.0225780 (PMC6894869; doi:10.1371/journal.pone.0225780)
Supplement: S2 Fig — (DOCX) [file pone.0225780.s005.docx]

**Supporting Information**

**Designing Small Molecule PCSK9 Inhibitors Guided by Simulated Annealing of Chemical Potential Simulations**

*Frank Guarnieri^1,2^, John L. Kulp Jr.^3^, John L. Kulp III^3,4^, Ian S. Cloudsdale^3^

^1^Center for Drug Discovery, Northeastern University, Boston, MA 02115 USA

^2^PAKA Pulmonary Pharmaceuticals, Acton, MA 01720 USA

^3^Conifer Point Pharmaceuticals, Doylestown, PA 18902 USA

^4^Department of Chemistry, Baruch S. Blumberg Institute, Doylestown, PA 18902 USA

*Corresponding author

Email: [frankguarnieri@yahoo.com](mailto:frankguarnieri@yahoo.com)

**Contents**

1. S1 Table. List of standard AMBER charges and custom derived charges for PCSK9-LDLR
2. S2 Table. List of fragments run on PCSK9
3. S3 Table. List of standard AMBER charges and custom charges for the CN-benzimidazole fragment bound to PCSK9
4. S1 Fig. Ball-and-stick representation of the connected path of interpenetrating atoms.
5. S2 Fig. Examples of π-π stacking.
6. S3 Fig. GAMESS input parameters
7. S4 Fig. Synthetic schemes for fragments and compounds

**S2 Fig.** π-π** Stacking.** N,N’-dimethyl-2,9-diazaperopyrenium (A) is a planar anticancer DNA intercalating agent. The π-π stacking sequesters some of the molecules decreasing efficacy and increasing toxicity. The out-of-plane di-isopropyl-benzyl derivative (B) causes a steric clash that prevents π-π stacking – it has increased potency and decreased toxicity. Pentoxifylline (C) was used as an additive to doxorubicin to prevent π-π stacking self-aggregation resulting in lower toxicity. Three molecules (D) of VUF9432 bind in a triplet π-stack in an acetylcholine binding protein discovered with a fragment screening assay (PDB ID 4BFQ).

**
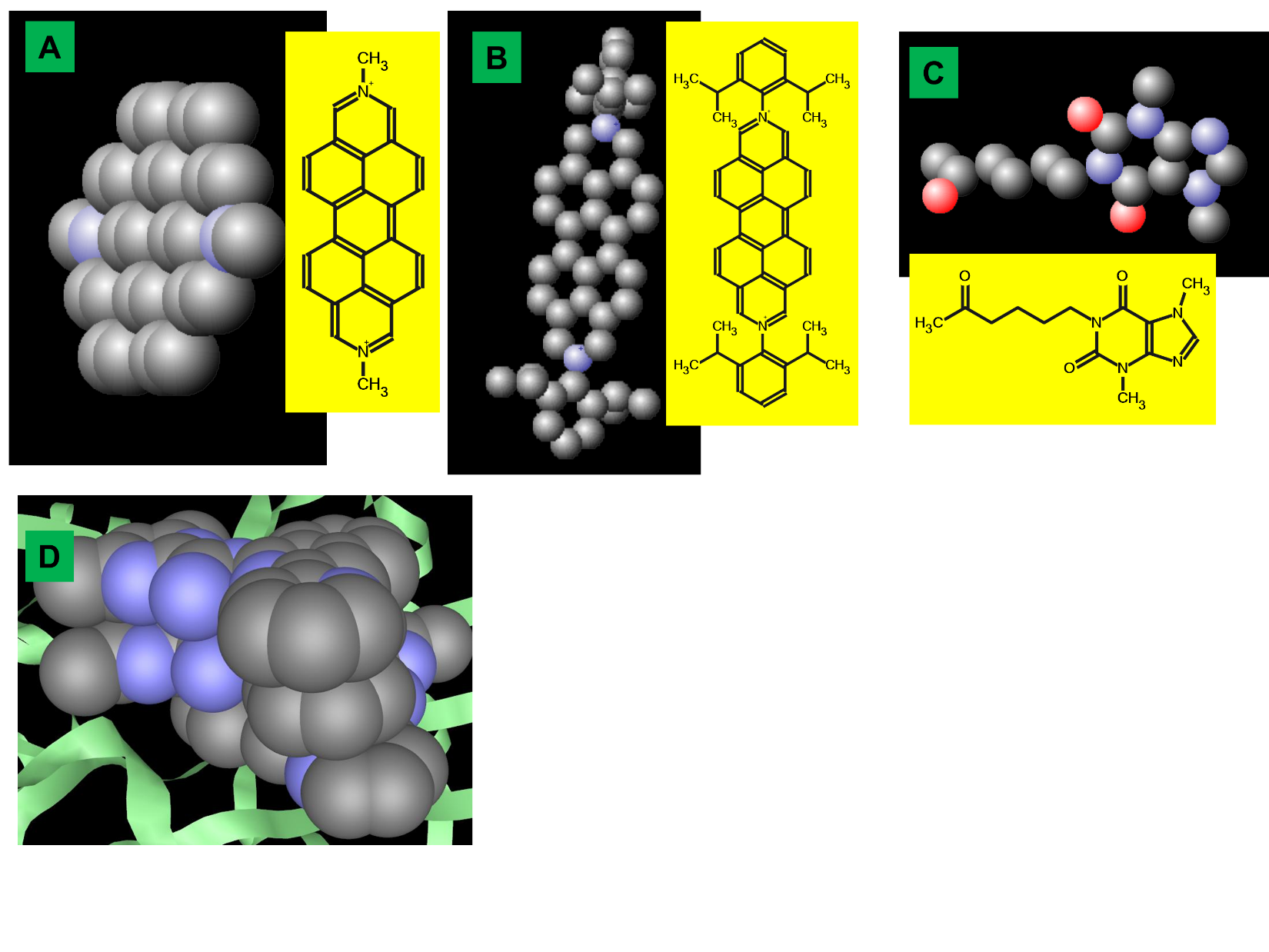
**
