# Supplementary material for: Fragment-based design of small molecule PCSK9 inhibitors using simulated annealing of chemical potential simulations
Source: PLoS One. 2019 Dec 5;14(12):e0225780. doi: 10.1371/journal.pone.0225780 (PMC6894869; doi:10.1371/journal.pone.0225780)
Supplement: S3 Fig — (DOCX) [file pone.0225780.s006.docx]

**Supporting Information**

**Designing Small Molecule PCSK9 Inhibitors Guided by Simulated Annealing of Chemical Potential Simulations**

*Frank Guarnieri^1,2^, John L. Kulp Jr.^3^, John L. Kulp III^3,4^, Ian S. Cloudsdale^3^

^1^Center for Drug Discovery, Northeastern University, Boston, MA 02115 USA

^2^PAKA Pulmonary Pharmaceuticals, Acton, MA 01720 USA

^3^Conifer Point Pharmaceuticals, Doylestown, PA 18902 USA

^4^Department of Chemistry, Baruch S. Blumberg Institute, Doylestown, PA 18902 USA

*Corresponding author

Email: [frankguarnieri@yahoo.com](mailto:frankguarnieri@yahoo.com)

**Contents**

1. S1 Table. List of standard AMBER charges and custom derived charges for PCSK9-LDLR
2. S2 Table. List of fragments run on PCSK9
3. S3 Table. List of standard AMBER charges and custom charges for the CN-benzimidazole fragment bound to PCSK9
4. S1 Fig. Ball-and-stick representation of the connected path of interpenetrating atoms.
5. S2 Fig. Examples of π-π stacking.
6. S3 Fig. GAMESS input parameters
7. S4 Fig. Synthetic schemes for fragments and compounds

**S3 Fig.** GAMESS input parameters for the custom charge calculation

**GAMESS input parameters Pass 1:**

! BioLeap GAMESS input - job: pcsk9_residues for jlkjr

$CONTRL SCFTYP=RHF MAXIT=200 RUNTYP=ENERGY EXETYP=RUN COORD=UNIQUE

UNITS=ANGS MOLPLT=.TRUE. NPRINT=-2 DFTTYP=B3LYP $END

$SYSTEM MWORDS=40 TIMLIM 6000 $END

$SCF DIRSCF=.T. $END

$BASIS GBASIS=MINI $END

$STATPT NSTEP=100 $END

$ELPOT IEPOT=1 WHERE=PDC OUTPUT=PAPER $END

$PDC PTSEL=CONNOLLY CONSTR=CHARGE PTDENS=0.21 $END

$GUESS GUESS=HUCKEL $END

$DATA

*molecule data*

$END

**GAMESS input parameters Pass 2:**

! BioLeap GAMESS input - job: pcsk9_residues for jlkjr

$CONTRL SCFTYP=RHF MAXIT=200 RUNTYP=ENERGY EXETYP=RUN COORD=UNIQUE

UNITS=ANGS MOLPLT=.TRUE. NPRINT=-2 DFTTYP=B3LYP $END

$SYSTEM MWORDS=40 TIMLIM 6000 $END

$SCF DIRSCF=.T. $END

$BASIS GBASIS=N21 NGAUSS=3 $END

$STATPT NSTEP=100 $END

$ELPOT IEPOT=1 WHERE=PDC OUTPUT=PAPER $END

$PDC PTSEL=CONNOLLY CONSTR=CHARGE PTDENS=0.21 $END

$GUESS GUESS=RDMINI $END

$DATA

*molecule data*

$END
