# Supplementary material for: Fragment-based design of small molecule PCSK9 inhibitors using simulated annealing of chemical potential simulations
Source: PLoS One. 2019 Dec 5;14(12):e0225780. doi: 10.1371/journal.pone.0225780 (PMC6894869; doi:10.1371/journal.pone.0225780)
Supplement: S4 Fig — (DOCX) [file pone.0225780.s007.docx]

**Supporting Information**

**Designing Small Molecule PCSK9 Inhibitors Guided by Simulated Annealing of Chemical Potential Simulations**

*Frank Guarnieri^1,2^, John L. Kulp Jr.^3^, John L. Kulp III^3,4^, Ian S. Cloudsdale^3^

^1^Center for Drug Discovery, Northeastern University, Boston, MA 02115 USA

^2^PAKA Pulmonary Pharmaceuticals, Acton, MA 01720 USA

^3^Conifer Point Pharmaceuticals, Doylestown, PA 18902 USA

^4^Department of Chemistry, Baruch S. Blumberg Institute, Doylestown, PA 18902 USA

*Corresponding author

Email: [frankguarnieri@yahoo.com](mailto:frankguarnieri@yahoo.com)

**Contents**

1. S1 Table. List of standard AMBER charges and custom derived charges for PCSK9-LDLR
2. S2 Table. List of fragments run on PCSK9
3. S3 Table. List of standard AMBER charges and custom charges for the CN-benzimidazole fragment bound to PCSK9
4. S1 Fig. Ball-and-stick representation of the connected path of interpenetrating atoms.
5. S2 Fig. Examples of π-π stacking.
6. S3 Fig. GAMESS input parameters
7. S4 Fig. Synthetic schemes for fragments and compounds

**S4 Fig.** Synthetic schemes for fragments and compounds

## Preparation of BLD_03493

A solution of 2g (10.87 mmol) of 2,6-difluoro-3-nitrobenzonitrile in methanol (10 mL) was added to a saturated solution of ammonia in methanol (20 mL) at O^o^C. The reaction was stirred for 30 minutes, then evaporated. The crude mixture was separated by flash chromatography to yield a mixture of **2a & 2b** (434 mg, 21% yield). To a solution of mixture of **2a & 2b** (434 mg) in THF (5 mL) was added Zn powder (1.9 g, 29.2 mmol). HCl solution (3.6 mL, 4 M, 14.58 mmol) was added dropwise at 0^o^C and the solution was stirred at RT for 1 h (95%). The crude mixture after extraction, was dissolved in 2/1 AcCN/H2O (9 mL) and treated with BrCN (232 mg, 2.187 mmol). The product 2-amino-6-methoxy-1H-benzo[d]imidazole-7-carbonitrile (BLD_03493) was obtained by flash chromatography (85 mg., 21% yield). Confirmed by LC-MS and 1H NMR.

## Preparation 8-bromobenzimidazole intermediates A1 and A2

Preparation of Acetylene intermediates B1, B2 or B3

**Preparation of Acetylene intermediate B4**
